# Palaeolithic polyhedrons, spheroids and bolas over time and space

**DOI:** 10.1371/journal.pone.0272135

**Published:** 2022-07-28

**Authors:** Julia Cabanès, Antony Borel, Javier Baena Preysler, Antoine Lourdeau, Marie-Hélène Moncel

**Affiliations:** 1 Département Homme et Environnement, Histoire Naturelle de l’Homme Préhistorique, HNHP‑UMR 7194 (MNHN, CNRS, UPVD), Alliance Sorbonne Université, Muséum national d’histoire naturelle, Paris, France; 2 Institute of Archaeological Sciences, Eötvös Loránd University, Budapest, Hungary; 3 Department of Prehistory and Archaeology, Universidad Autónoma de Madrid, Ciudad Universitaria de Cantoblanco, Madrid, Spain; Griffith University, AUSTRALIA

## Abstract

Polyhedrons, spheroids and bolas (PSBs) are present in lithic series from the Lower Palaeolithic onwards and are found in several regions of the world. Nevertheless, very little is known about them. We propose here to summarise, illustrate and discuss the current state of our knowledge about these artefacts. Based on the available data in the literature and on our observations of several collections, we set up a database comprising 169 Palaeolithic assemblages with PSBs. Thanks to the statistical analysis of these data, we aim to highlight potential relationships between PSB characteristics (e.g., quantity, raw material) and assemblage composition and context, according to regions and chrono-cultural attributions. We also aim to discuss the question of artefacts from possible independent local histories, especially in Northwest Europe, where these objects are scarce. Our study concludes that hard stones (stones with high resistance to a physical constraint) available locally were generally selected to produce PSBs. Soft sedimentary rocks are suitable for their manufacture, and were selected too, whereas siliceous materials were left aside. We hypothesise that the scarcity of PSBs in Northwest Europe could result from a combination of cultural and environmental factors: it could be part of a regional tradition, influenced by the abundance of siliceous materials in the environment. In this region where the lithic production is widely made of flint, even though other materials were available, objects made from hard stones are scarce, resulting in a toolkit with only rare PSBs and cleavers. Was flint too brittle for the functions of PSBs? Raw materials of PSBs are often similar to those of heavy-duty tools in assemblages, which could provide other clues about their functions (e.g., tasks requiring a resistance to shocks). It is possible that their raw materials partly conditioned their final shape. PSBs can comprise a wide variety of artefacts, that for some could have change of status (e.g., from cores to percussive tools), diffused, adapted but also reinvented over two million years.

## Introduction

Palaeolithic polyhedrons, spheroids and bolas (PSBs) are enigmatic spherical stone objects as their functions and manufacture modes are still poorly known. They are present in archaeological assemblages from the Oldowan onwards, and are among the oldest manufactured stone items. The earliest occurrences were discovered in East and North Africa, dated at 2.0–1.9 Ma at Ewass Oldupa, Tanzania [1]. These rounded morphologies lasted for nearly two million years as they are found until the Neolithic (e.g., [2]) and even historical periods (e.g., [3]). Different hypotheses have been put forward as to their possible functions (e.g., exhausted cores, hammerstones or projectiles) [4–13]. Interestingly, PSBs seem quite common in African and Asian assemblages whereas they are less documented in European sites. They may be a means of identifying modes of adaptation of populations to varied and new environments (mineral and vegetal), inducing the loss of certain elements of the ‘tool kit’ or a reorientation of manufacturing methods, depending on needs and available materials.

Rounded artefacts are well documented for more recent periods (e.g., [3, 14, 15]), but only a few studies have attempted to analyse variations in the frequencies and characteristics of PSBs across regions, time and chrono-cultural attributions during the Palaeolithic. In particular, the frequency of PSBs was considered by Mary Leakey [5] as a diagnostic marker of cultural variability of the Developed Oldowan. Willoughby [16] shares this opinion and highlights a difference in hominin investment to produce PSBs between the Oldowan and the Acheulian: PSBs became more rounded after the Acheulian, with more polyhedrons during the Oldowan [6, 17–19].

This paper presents and analyses a large database of 169 Palaeolithic assemblages with PSBs from the Oldowan, Core-and-Flake-type industries (Mode 1-type), Acheulian, Middle Stone Age and Middle Palaeolithic sites, across Africa, Eurasia and the Levant. Thanks to the statistical study of these data, we aim to highlight potential relationships between the presence and quantities of PSBs in sites, the context and composition of assemblages and regional or chrono-cultural variabilities. This will contribute to the debate on the presence of ‘apparently identical objects’ in archaeological records distant in time and space, issued from independent local histories and made by different hominin species. Particularly, we aim to investigate the possible reason(s) (environmental, functional, cultural factors?) for their scarcity in Europe and in the later periods of the Palaeolithic. The results will also allow us to discuss potential selection of materials for the manufacture of these objects (e.g., types of raw material, their properties, comparison with the raw materials of the other items in associated assemblages), which can give clues about their possible functions. Finally, in some cases, we hypothesise a relationship between the degree of roundedness of PSBs and their raw materials, and discuss the question of intentional cubical to rounded final morphologies.

## State of knowledge on PSBs

### A large variety of artefacts and definitions

PSBs include a wide range of objects in terms of morphology, size and weight, ranging from cubical sharp-edged items to smooth perfectly-rounded artefacts. This variety of forms, combined with the multiplicity of (generally) undemonstrated theories about their functions, led to the emergence of multiple definitions. In the literature, their classification varies depending on the archaeological contexts and authors. They are sometimes classified as multipolar or multifacial cores (e.g., [20, 21]), modified battered pieces (e.g., [21]), tools (e.g., [8]), or all of these categories (e.g., [5]). In the past, PSBs were described as *hâches celtiques* by Boucher de Perthes [22] when he discovered these shaped rounded stones in the Somme Valley (France), generally pecked, made from sedimentary rocks (flint, chalk and sandstone) or granite. Then, depending on the authors, a same PSB can be classified as a bola, spheroid, polyhedron or stone ball, flexible terms incorporating a more or less wide range of morphologies depending on studies. For example, Clark [23] only considered the perfectly rounded items as “stone balls” while Gruet [24] described a 75 cm high pile of “stone balls” in limestone at the El Guettar site (Tunisia), including a great variety of PSBs with cubical to more rounded morphologies, sometimes without clear human modifications. Indeed, some authors include manuports (natural spherical stone without human modification, selected and transported by hominins) in their PSB classification. This is the case of Kleindienst [25] who distinguished three categories of PSBs: (1) missiles: manuports, (2) polyhedrons: facetted items, with visible negatives of removals or (3) bolas: nearly perfectly rounded objects without visible facets, smoothed by battering.

PSBs also used to be named according to their putative functions. At Festons (France), Pittard and de Saint-Périer [26] mentioned spherical pieces (350g to 600g) as *casseurs d’os*, presuming that they were used for bison and horse bone breakage to access marrow. In addition to these items, they also described approximately 15 limestone and quartz stones rounded by crushing. They called them *bolas* (with diameters of 57 mm to 63 mm, 240 g to 250 g) and suggested that they were used to capture animals, in reference to South American ethnographic stone or metal balls attached to ropes, composed of one to three pieces. *Bolas* were used by Amerindians and *gauchos* from Argentina, Uruguay and south Brazil as projectiles mainly to capture animals, and were also functional for combat. This hypothesis of PSBs used as projectiles convinced several authors (e.g., [5, 26–30] and is still popular today. Louis Leakey [27] made PSBs widely known by encouraging this missile theory. According to him, the discovery of rounded items by groups of three in Olorgesailie assemblages (Kenya), combined with his experimental work, corroborated this theory [31]. This hypothesis has long been based on actualism with comparisons to the South American *bolas*. Indeed, these ethnographic artefacts gave their name to the prehistoric objects, and recent quantitative studies (volumetric, ballistic and mass distribution) provide objective data that could support the idea of some Palaeolithic PSBs (and manuports) being missiles [29, 30].

In her pioneering classification of the Oldowan industries, Mary Leakey [5] categorised PSBs typologically based on direct observation of their morphological similarity to a sphere and to the smoothness of their surfaces: (1) polyhedrons: “angular tools with three or more working edges, usually intersecting. The edges project considerably when fresh, but, when extensively used, sometimes become so reduced that the specimens resemble subspheroids”, (2) spheroids: “include some stone balls, smoothly rounded over the whole exterior. Faceted specimens in which the projecting ridges remain or have been only partly removed are more numerous”, and (3) sub-spheroids: “similar to the spheroids but less symmetrical and more angular”. This classification is still used by some authors while criticised by others. It has for instance been revised by Sahnouni et al. [9], who also discerned these three types of PSBs: polyhedrons, objects with a minimum of three flaked faces mostly displaying obtuse core angles; sub-spheroids, with more obtuse angles than polyhedrons, widely flaked on three or more faces; and finally spheroids, artefacts flaked over much, if not all of their surfaces with very obtuse angles and a relatively spherical shape.

### Rounded shape: A predetermined morphology?

In the literature, two main contrasting interpretations of PSBs are put forward. 1) The first considers spheroid morphologies as forms of exhausted cores, by-products of a debitage process. These pieces would subsequently have been used in battering activities (e.g., [7, 9]). The different types of PSBs would be phases of a debitage reduction process with no preconceived techno-typological purposes. To investigate this first hypothesis, Sahnouni, Schick and Toth led functional and experimental replicative studies [9] on the limestone PSBs from Ain Hanech (Algeria). They applied a hypothetico-deductive approach. Their aim was not to reproduce the original production operative chain of these PSBs, but rather to reduce the volume of pebbles and cobbles as much as possible and generate ‘usable’ flakes [9]. Their conclusion was that spheroids can be exhausted cores resulting from intense debitage, subsequently used as hammerstones. Cobble blanks are big enough at Ain Hanech to generate potentially functional or transformable flakes. However, as De Weyer highlights, this study raises some issues: the flakes produced are in a soft raw material and 2 cm long on average, which seems quite small for the manufacture of tools or to be used in a task. Furthermore, the large majority of flakes used at Ain Hanech are in flint, potentially implying that limestone was not the most suitable material for producing flaked tools [32]. Nevertheless, the conclusion of Sahnouni et al. is in keeping with the earliest experiments of Toth [33, 34] on the PSBs of Koobi Fora (Kenya). The authors conclude that intensive debitage (in order to produce usable flakes) naturally leads to a polyhedral core shape, and this becomes increasingly spherical throughout the reduction process. Moderate reduction would produce unifacial or bifacial choppers [9]. More particularly, Schick and Toth [7] suggested that exhausted quartz cores would have been systematically used as battering instruments, and would have been accidentally increasingly rounded as a result of recurrent percussive activities.

**Fig 1:**
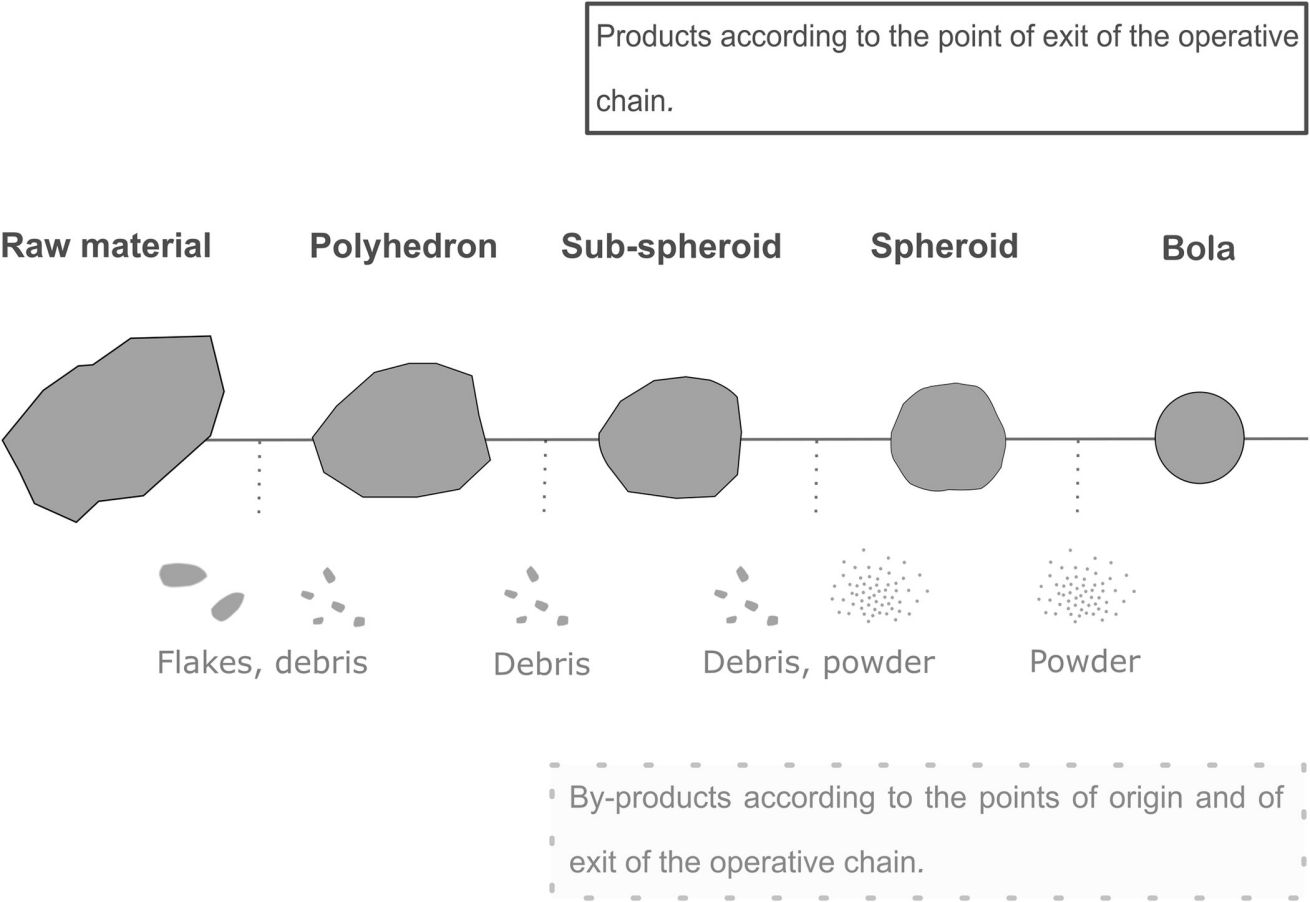
Hypothesis of the linear operative chain of PSB manufacture (based on Texier & Roche 1995).

2) The second hypothesis proposed by Texier and Roche [8] groups polyhedrons, sub-spheroids, spheroids and bolas as three segments of the same preconceived shaping operative chain (Fig 1), from the angular polyhedron shaped in a more spherical form (sub-spheroids, spheroid), and finally pecked into a perfectly smoothed rounded stone (*bola*). The aim of the process would be to purposefully obtain a spherical artefact according to reasoned organised final shaping, in order to use it for a predefined (unknown) function [8, 35]. Since PSBs can display a totally battered surface, Texier and Roche [36] exclude the possibility that they were simple hammerstones, since the latter generally only comprise one or two battered faces. This is contradicted by the conclusions of Assaf and Baena [37] and Assaf et al. [12], who proved that the spheroids from Qesem, Israel, could have been used as hammers for bone breakage. Assaf and Baena [37] also suggest that the large removals on the surface of these pieces were not due to use, but rather the result of an intentional shaping process to obtain the desired objects. Contrary to Sahnouni and Toth’s conclusions [9, 33], experiments by Texier and Roche [8], based on the archaeological material from Isenya (Kenya), show that removal organisation is different from the volume management of a simple core, suggesting a global view of the volume. They highlight that a rounded volume tends to reduce the possibilities of extracting flakes. After the analysis of more than 500 PSBs from Isenya, Texier and Roche [8] noted that hominins selected blanks for the production of PSBs, choosing pieces with a natural spherical shape and thus reducing the first phase of the operative chain, the most productive in flakes. Once again, this would contradict the theory of the by-product core. Another argument of Texier and Roche is that at Isenya, only a few flakes derive from the operative chain of polyhedron production, and these flakes are neither usable nor transformable by shaping or retouch (poor quality raw material and inadequate flake morphology). However, this last statement does not contradict Sahnouni’s experimental conclusions [9] since Sahnouni did not aim to reduce blocks in order to obtain a spherical morphology but aimed to produce flakes. Thus, both models can be demonstrated but are not compatible with all sites. Knapping experiments of Toth [33] and Texier and Roche [8] concluded that intentionally obtaining PSBs is a complex process. Texier and Roche [8] highlight that the difficulty lies in removal organisation, which totally differentiates this reduction process from bifacial shaping and from debitage.

Intermediary scenarios have been proposed between these two opposed interpretations. Mora and de la Torre [19] base their analyses on Schick and Toth’s study of quartz blocks possibly rounded by percussive activities [7]. Like Jones [18], they concluded that the PSBs from Olduvai were rounded during battering activities. In contrast with the main assumptions, some authors also argued that polyhedrons could be purely coincidental or the consequence of a knapper’s lack of experience [33, 38].

Willoughby [16] underlines that PSBs appear at Olduvai at the same time as anvils, suggesting that these PSBs could be hammers combined with anvils in pounding activities. In this case, PSBs would not be deliberately rounded but would have acquired this spherical shape by repeated use as hammers [17]. She also interprets the pecked cupulas on some anvils as the result of the preparation of the anvil before use. On the contrary, Texier and Roche [8] suggest that these cupulas could result from use. Based on this potential correlation between PSBs and anvils at Olduvai [16], Texier and Roche [8] argue that producing PSBs with a hammer on a hard surface would allow for better control of the percussion process. This would result in bipolar percussion, generating multiple battered zones that would round the PSB. This point of view coincides with innovative studies led by Clark in 1955 [23], who proposed that PSBs were worked on an anvil until they obtained their spherical shape. Clark [23] suggests that battering ridges would be a deliberate part of the manufacture process to remove protruding edges. However, he does not exclude the possibility that PSBs could have been hammerstones involved in nut crushing or knapping, although his main idea was that PSBs were missiles.

### Polyhedrons, spheroids and bolas: Three stages of a same reduction process?

Both main hypotheses about the nature of PSBs (predetermined tools vs. exhausted cores) agree that polyhedrons, spheroids and bolas are stages of the same technological trajectory. Texier and Roche [8, 36] demonstrate this for the assemblages of Isenya, as does Sahnouni at Ain Hanech [9, 39]. Nevertheless, this linear reduction between PSBs has been contested in several cases with morphometric assertions (as polyhedrons are smaller than spheroids in some assemblages) and because in some sites, polyhedrons, spheroids and bolas are respectively made from different raw materials, which according to Jones [18], are clues that polyhedrons and spheroids do not belong to the same sequence. Indeed, this difference in raw materials can be observed in several sites, such as at Isenya where bolas are made from quartz, whereas polyhedrons and spheroids are mostly in nephelinic phonolite (less frequently in quartzite and quartz). This is also the case in Beds I and II of Olduvai, where polyhedrons are made of lavas whereas spheroids and bolas are almost all in quartz [18]. Based on diacritic schemes, De Weyer [11] also argues that at the site of Ounjougou (Mali, Oldowan cultural facies attribution), polyhedrons, spheroids and bolas stem from different operative chains.

### Functional assumptions

The functional interpretation of PSBs has given rise to theories and debates for decades. These pieces often present macro-traces on their protruding ridges, generally described as crushing, pecking or battering traces (e.g., [4, 40–43]. As discussed above, a popular theory considers PSBs as missiles or *bolas* [5, 26–30]. Authors presenting PSBs as hammerstones also abound (e.g., [44]), some arguing that they were rounded without predetermination in battering activities [18], or exhausted cores secondarily reused as hammers for tool manufacture [7, 9]. According to the recent experimental and use-wear study of Assaf et al. [12] and Assaf and Baena [37], the spheroids from Qesem Cave were used as hammers in bone breakage to access marrow. Isaac [45] shares this hypothesis of bone breaker tools. Others assume that some PSBs were involved in pounding activities [23], or used as club heads [28]. For her part, Willoughby [6, 17] does not exclude any of these scenarios, but is more sceptical about the use of PSBs as throwing weapons since some of them are quite heavy (several kilograms). She also proposed that PSBs could have been vegetal processors [16]. Thus, in the same way as manufacture modes, the debate about the functions of PSBs still remains open and no single scenario can be extended to all sites.

This uncertainty surrounding PSBs is partly due to the scarcity of functional studies, since they are barely mentioned in the literature and even more rarely described or illustrated. The lack of homogeneity regarding their classification and denominations can lead to misunderstandings when illustrations are lacking. Recent interest in these objects and a few focused studies have emerged over the past few years [10–13, 29, 30]. For instance, Titton et al. [13] carried out a study of the five PSBs from the Oldowan site of Barranco León (Spain), combining diacritical and 3D geometric morphometric analysis with an evaluation of the raw material (limestone) and of percussive use-wear (types and localisation). De Weyer [11, 32] proposed an analysis of the PSBs from Ounjougou with technological, techno-functional and morphometric approaches, comparing these pieces with PSBs from other sites such as Olduvai Gorge and Ain Hanech. The most extensive and significant works on PSBs may be attributed to Willoughby (e.g., [6, 17]) for her techno-functional analysis, to Sahnouni, Schick and Toth for their experimental and morpho-technical studies (e.g., [9, 33, 46]), and to Texier and Roche for their experimental and technological approaches [8, 36, 47].

## Material and methods

The data presented here were gathered from the literature and from discussions with the authors of published studies. When possible, we also added data by direct observation of the archaeological material. We report here 169 Palaeolithic assemblages with PSBs.

When available, the age of each assemblage and dating method were noted, as well as its chrono-cultural attribution (Oldowan, Core-and-Flake-type industry, Acheulian, MSA, Middle Palaeolithic), in order to compare PSBs across time, regions and cultural attributions. Oldowan and “Core-and-flake” industries share similar features, but the term “Oldowan” is more used to describe the African series. Eurasian series show specific features related to the environments and adaptation of the hominins to these territories (e.g., raw materials, landscapes). Thus, in our opinion, it is better to use “Core-and-flake” term to describe human behaviours outside of Africa, and show common or different strategies between the African and Eurasian series.

**Fig 2:**
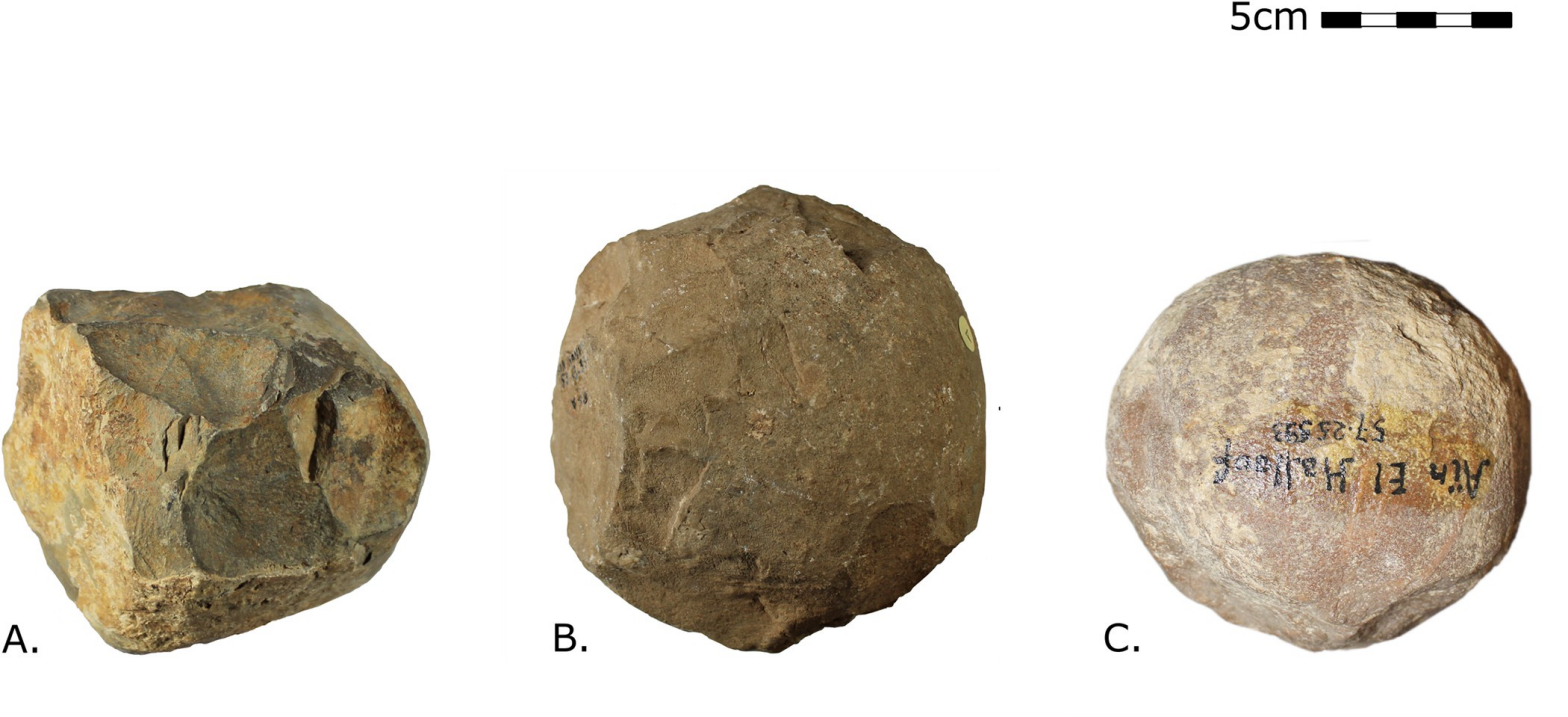
Illustration of the three typological categories of PSBs used in this study. (A) Polyhedron in limestone from Ain Hanech (Algeria), N° inv. 52.1.13, curated at the Institut de Paléontologie Humaine of Paris. (B) Spheroid in limestone from Ain Hanech (Algeria), N° inv. 53.3.3 curated at the Institut de Paléontologie Humaine of Paris. (C) Bola in limestone from Aïn el Hallouf (Morocco), N° inv. 57.25.593 curated at the Musée de l’Homme. Photos: Julia Cabanès.

The quantities of PSBs (respectively: number of polyhedrons, spheroids, bolas and total number of PSBs) per assemblage were documented when possible. Since precise counts of PSBs are sometimes lacking, the presence of PSBs is also reported as “Present”, “Absent”, “Uncertain”, “No Data”. The “Uncertain” value was attributed when we were unable to classify the PSBs from an assemblage in one category, according to the definition in the literature or due to the lack of illustrations. For the sake of consistency, and since the same term can have diverse meanings for different authors, we reclassified all the PSBs of each assemblage of the corpus according to a same definition of polyhedron, spheroid and bola. Our classification throughout this study is based on that defined and illustrated in previous studies (e.g., by Mary Leakey [5] or Texier and Roche [47]). Yet, we kept the original appellation given to each PSB in a comment box in the database. In the literature, little is known about the manufacture and use modes of these items, and it is still difficult to generalise typo-technological or functional concepts to different categories of PSBs. Thus, as it is the case in most of the studies about PSBs, our definitions are mainly based on a concept of morphological classification according to the roundness of the pieces. We did not used a “sub-spheroid” category, since such proximity with the classes of polyhedrons and spheroids may increase the subjectivity of the classification. We considered a “bola” morphological class, as did for instance Texier and Roche [47]. Only anthropically manufactured artefacts are considered, excluding naturally rounded stones with or without modification by use. We used the following definitions:

- Polyhedrons: angular item with at least three working edges, often displaying a cubical shape, with protruding ridges generated by removal scars (Fig 2A).
- Spheroids: spherical object with visible ridges and facets, that could have been partly pecked but not over the whole surface (Fig 2B).
- Bolas: perfectly spherical items where angularities have been removed by pecking or any other anthropical process, resulting in a perfectly smoothed rounded stone ball (Fig 2C).

Raw materials were also documented to discuss potential correlations between materials and types of PSBs in time and space, and to evaluate raw material management of the different types of PSBs. The presence of PSBs in each raw material was reported using the categories “Present”, “Absent”, “Uncertain”, “No Data” and, for each assemblage, the frequencies of PSBs in each raw material were recorded. The value “Uncertain” was assigned when there were several possibilities of attribution of raw materials according to the data. For instance, when the only information was that all the lithics of the assemblage were in quartz and quartzite, we attributed “Uncertain” in both “Quartz” and “Quartzite” categories, and “No” in all the other classes of materials. Raw materials and proportions of PSBs in assemblages were compared to proportions of the associated cores, hammerstones, light-duty tools (LDT, including flaked tools, flakes, fragments and debris) and heavy-duty tools (HDT, including cobble tools, handaxes, cleavers and all categories of shaped objects). PSB blank types were also noted (pebble, cobble, block, block fragment, slab, bipolar core, flake, nodule, debris and No Data). PSB raw material types were compared to those of other artefacts in the assemblage (HDTs, LDTs, cores and hammerstones) to illustrate potential similarities.

The context and environment of assemblages is reported by several variables: type of environment (open, mosaic, wetland, woodland and tropical forest), climate (cold, arid, semi-arid, temperate, continental, humid, sub-tropical and No Data), and presence of water sources nearby (Presence, Absence, No Data). The occurrence of different species of large mammals was documented (for *Cervidae*, *Equidae*, *Bovidae*, *Proboscidea*, *Suidae*, other: Presence, Absence, No Data), as well as for remains of anthropically broken bones (Presence, Absence, No Data). The type of occupation and activities described in published papers are also examined. Some categories may seem redundant (e.g., habitat, camp site, base camp, living site and residential) and were thus grouped together. Some values can also be considered as sub-categories of others (e.g., butchery, scavenging and bone processing). The data considered are long, short, seasonal, recurrent occupations; hunting, kill site, butchery, scavenging, bone processing, plant processing, wood processing, knapping, living site and No Data.

The spatial location of PSBs in the assemblage is described when known, along with that of the other directly associated artefacts. The other types of lithics associated with PSBs were also listed for each assemblage to assess whether PSBs were recurrently related to specific objects. The main types of debitage associated with the assemblages were documented (unipolar, bipolar, bipolar on anvil, discoid, proto-Levallois, Levallois-like, Levallois, opportunistic, Kombewa, hierarchical, laminar, Quina, No Data). This parameter was generally not, or only partially or disparately available in the literature, and cannot always be taken into consideration here.

Descriptive statistics were used to compare the quantities and raw materials of PSBs (respectively: for PSBs in general, polyhedrons, spheroids and bolas) in assemblages between regions and chrono-cultural attributions. Statistical tests were also performed. As the data did not follow a normal distribution, non-parametric Kruskall-Wallis tests and Kruskall-Wallis Effect Size tests were used. The results are available in S1 Table. If the p-value was lower than the selected alpha value of 0.05, a pairwise comparison was performed with a Dunn test. A Bonferroni correction for multiple testing was applied to the p-values. These tests excluded assemblages for which no quantitative PSB data were available. For analyses of quantities according to chrono-cultural attributions, assemblages of unknown chrono-cultural attribution were not considered. Since data are missing for other parameters for many assemblages, they were not statistically processed, but the number of assemblages were compared in order to describe the main tendencies. Statistical tests and charts were computed in R v4.1.038 [48] via RStudio v1.4.1106. The libraries used were: xlsx [49], readxl [50], writexl [51], FactoMineR [52], tidyverse [53], ggpubr [54], rstatix [55], FSA [56], plyr [57].

This study attempts to highlight correlations between composition and context of assemblages that are variable, distant in time and space, and to interpret it at the light of the diversity of their context (e.g., chronological, geographical, environmental). For instance, the raw materials selected by hominins to manufacture PSBs will depend on the geological composition of the environment, that varies from one site to another. In the paper, we detail specifically which raw materials are selected to produce PSBs, and in supplementary material, we list for each site the raw materials of associated LDTs and HDTs. When possible, we also distinguished raw materials preferably selected as blank for cores and hammerstones. Then, we could say for each assemblage to which one of these group PSBs were the closer, in terms of raw material. We could also say which material was preferentially selected when available, and which ones were systematically avoided to produce PSBs, even though selected to produce other objects of the assemblage. This allowed to limit the bias due to the geological composition of the environment, since we compared specifically raw materials of objects from a same assemblage. When possible, we also recorded the availability of raw materials in the environment of sites, particularly for siliceous materials. In a first step, we describe results as they are (how many polyhedron, spheroids and bolas in each raw material), and secondly, we synthesise and interpret it keeping in mind the potential bias, classifying raw materials in categories according to their common properties (e.g., hard, brittle, igneous, siliceous).

We included in the study all the sites that yielded PSBs that we found in the literature, at the expanse of obtaining categories with the same size. The fact that more or less sites yielded PSBs according to cultures and regions is precisely interesting, and this discrepancy is kept in mind when discussing the results. All the statistical tests performed are adapted to the comparison of samples of different size.

Some sites yielded more PSBs than others, what could influence the results regarding the quantities of PSBs in each raw material. In order to limit this phenomenon, we always considered three parameters: the quantity of PSBs in a raw material out of the total number of PSBs of the sample, the percentage it represents, and the number of assemblages that yielded at least one PSB in the raw material considered. The few sites that particularly raised this problem of overrepresentation are mentioned and discussed in the study. This phenomenon is fully considered and discussed, and thus does not bias the study.

Generally, the earlier the site has been excavated, the lower is the frequency of LDTs in assemblages and the more HDTs are represented. This could suggest a bias of collect in the earliest excavations, where the largest and/or the most “aesthetic” objects were collected, at the expense of flakes and debris for instance. However, this tendency is not verified for the PSBs frequency, that does not fluctuate with the date of excavation.

Detailed information could not be collected for all assemblages, as data availability is disparate depending on sites. We found data on Asian sites to be particularly scarce compared to the number of PSBs actually found in that part of the world. For instance, the numerous bolas discovered in Southeast Asia, such as in Sangiran in Central Java, or the abundant PSBs from Chinese and South Korean sites (e.g., [58]), are nearly absent from the available literature. This may be due to a lack of precise archaeological context for these discoveries or to the necessity for deeper investigations into more local literature and languages which currently not accessible to us. This should be the focus of future research and is the reason why most Asian sites were excluded from our statistical analyses.

## Results

The 169 assemblages of the corpus are listed in S2–S6 Tables with detailed information. Data regarding PSBs were compiled from the articles listed in S1 Text and by personal observations.

### Quantities of PSBs in assemblages

**Fig 3:**
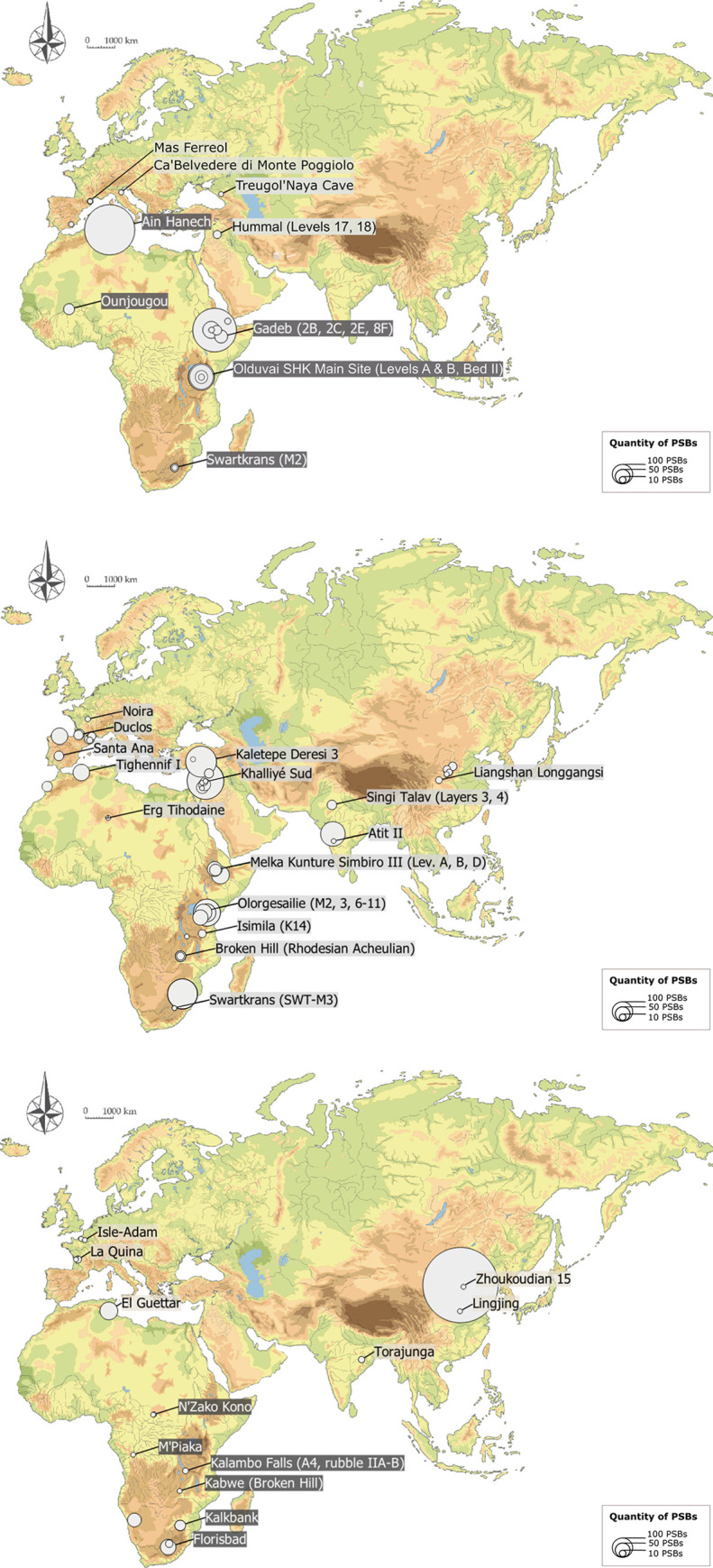
Distribution and quantities of PSBs in Africa, Eurasia and the Levant according to chrono-cultural attributions. (A) In the Oldowan (dark-grey site labels) and Core and Flake (light-grey site labels). (B) In the Acheulian. (C) In the MSA (dark-grey site labels) and Middle Palaeolithic (light-grey site labels).

**Fig 4:**
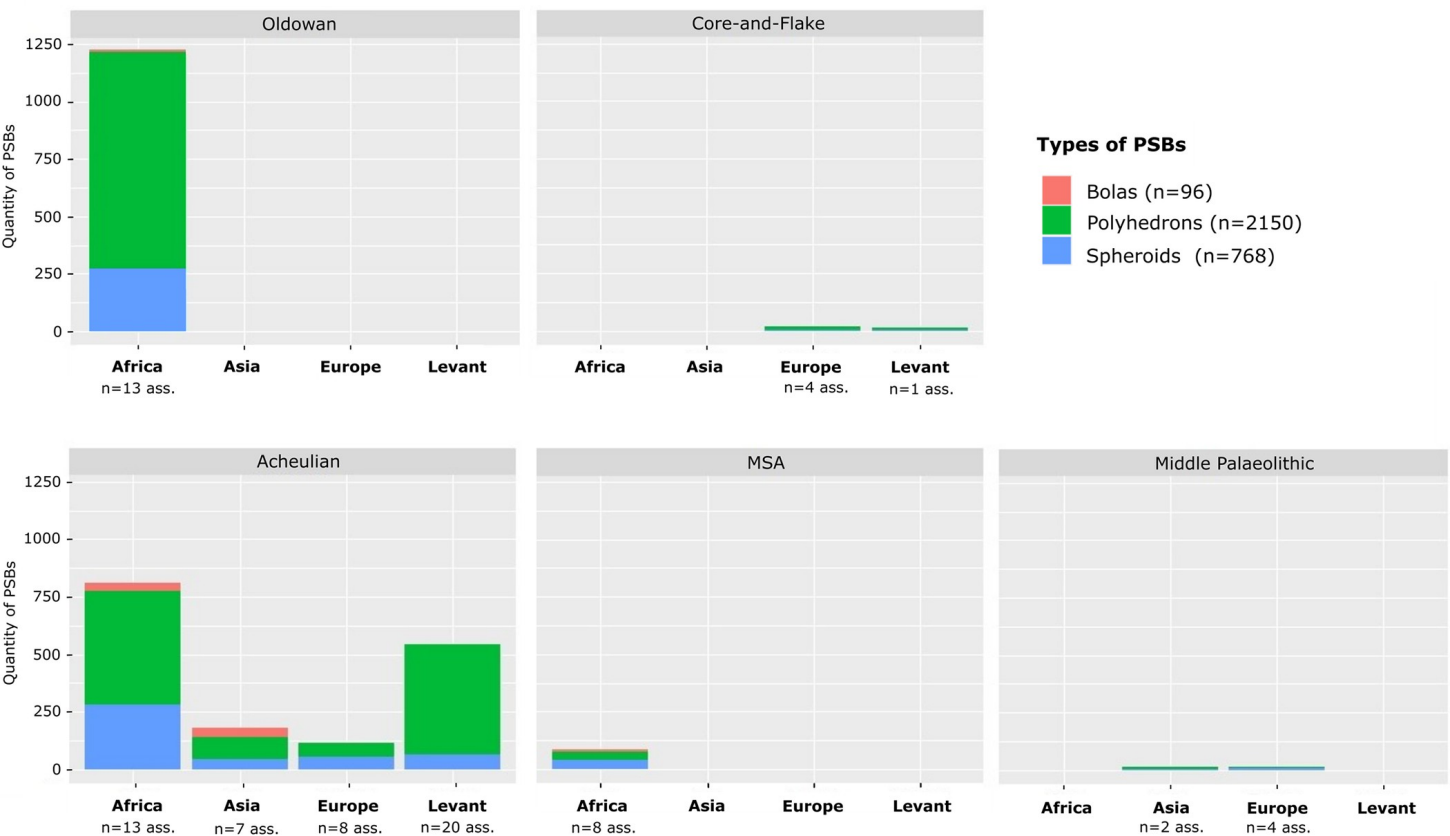
Quantities of polyhedrons, spheroids and bolas in assemblages according to chrono-cultural attributions and regions. Ass.: Assemblages.

A synthesis of the Dunn test results regarding quantities of PSBs according to regions and chrono-cultural attributions is presented in Table 1. In a nutshell, there are significantly fewer PSBs in European assemblages, especially compared to East and North Africa and to the Levant (for PSBs in general, but also respectively regarding polyhedrons, spheroids and bolas) (Table 1, Figs 3 and 4). PSBs are also scarce in Central Africa, which can partly be due to the paucity of documented prehistoric sites in this region, but not as much as for West Africa or Southeast Asia.

**Table 1:** Statistically significant differences in quantities of PSBs according to regions and chrono-cultural attribution.

Criteria	PSBs	Polyhedrons		Spheroids	Bolas
		All assemblages	Assemblages with polyhedrons	All assemblages	All assemblages
**Large geographical areas** (Africa, Asia, Europe, Levant)	• ***Europe*** vs. **Africa** (p = 0.0015)	• ***Asia*** vs. **Africa** (p = 0.0295)	• ***Europe*** vs. **Africa** (p < 0.001)	• ***Europe*** vs. **Africa** (p = 0.0027)	• ***Levant*** vs. **Africa** (p = 0.0101)
	• ***Europe*** vs. **Levant** (p = 0.0237)	• ***Asia*—Levant** (p = 0.0056)	• ***Europe*** vs. **Levant** (p = 0.0188)	• ***Europe*** vs. **Asia** (p = 0.0826)	• ***Europe*** vs. **Africa** (p = 0.0515)
**Sub-geographical regions** (East Africa, West Africa, Central Africa, North Africa, South Africa, East Asia, South Asia, South-East Asia, Europe, Levant)	• ***Europe*** vs. **East Africa** (p = 0.0421) • ***Europe*** vs. **North Africa** (p = 0.0341)	• ***East Asia*** vs. **East Africa** (p = 0.0199) • ***East Asia*** vs. **North Africa** (p = 0.0035) • ***East Asia*** vs. **Levant** (p = 0.0038)	• ***Europe*** vs. **East Africa** (p < 0.001)		• ***East Africa*** vs. **South Africa** (p = 0.0357) • ***East Asia*** vs. **South Africa** (p = 0.0223) • ***Europe*** vs. **South Africa** (p = 0.0012) • ***Levant*** vs. **South Africa** (p < 0.001)
**Chrono-cultural attributions** (Oldowan, Core-and-Flake-type, Acheulian, MSA, Middle Palaeolithic)		• ***MSA*** vs. **Oldowan** (p = 0.0673)	• ***Core-and-Flake*** vs. **Oldowan** (p = 0.0278)		• ***Acheulian*** vs. **MSA** (p = 0.0575) • ***Core-and-Flake*** vs. **MSA** (p = 0.0679) • ***Oldowan* vs. MSA** (p = 0.0797)

In italics: the sample with less PSBs of the pair. p: p-values of Dunn test with Bonferroni correction.

**Fig 5:**
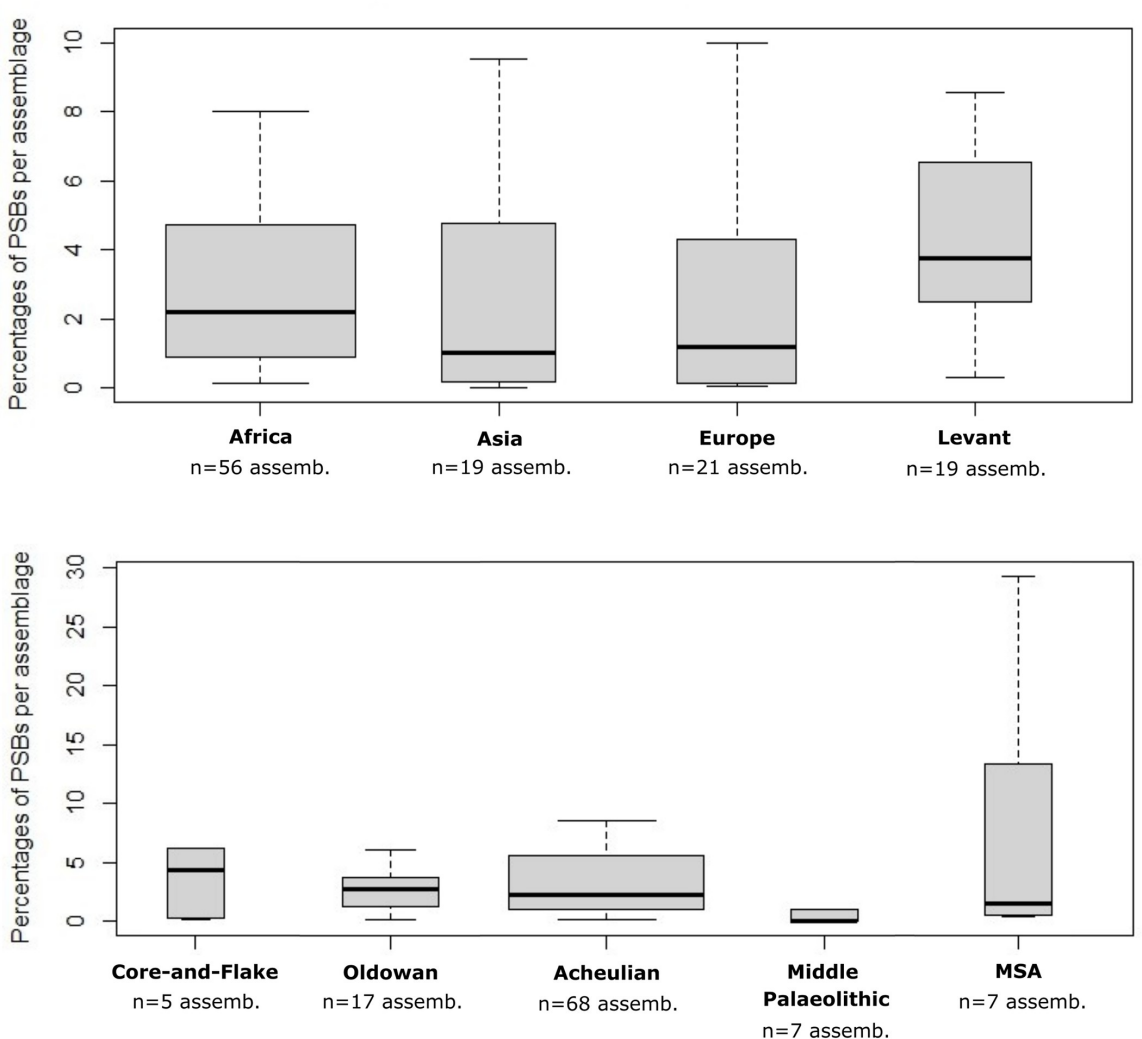
Proportions of PSBs in lithic assemblages according to regions and chrono-cultural attributions. The width of each column is proportional to the number of assemblages considered. Assemb.: assemblages. Outliers not displayed in the construction of the graph.

**Fig 6:**
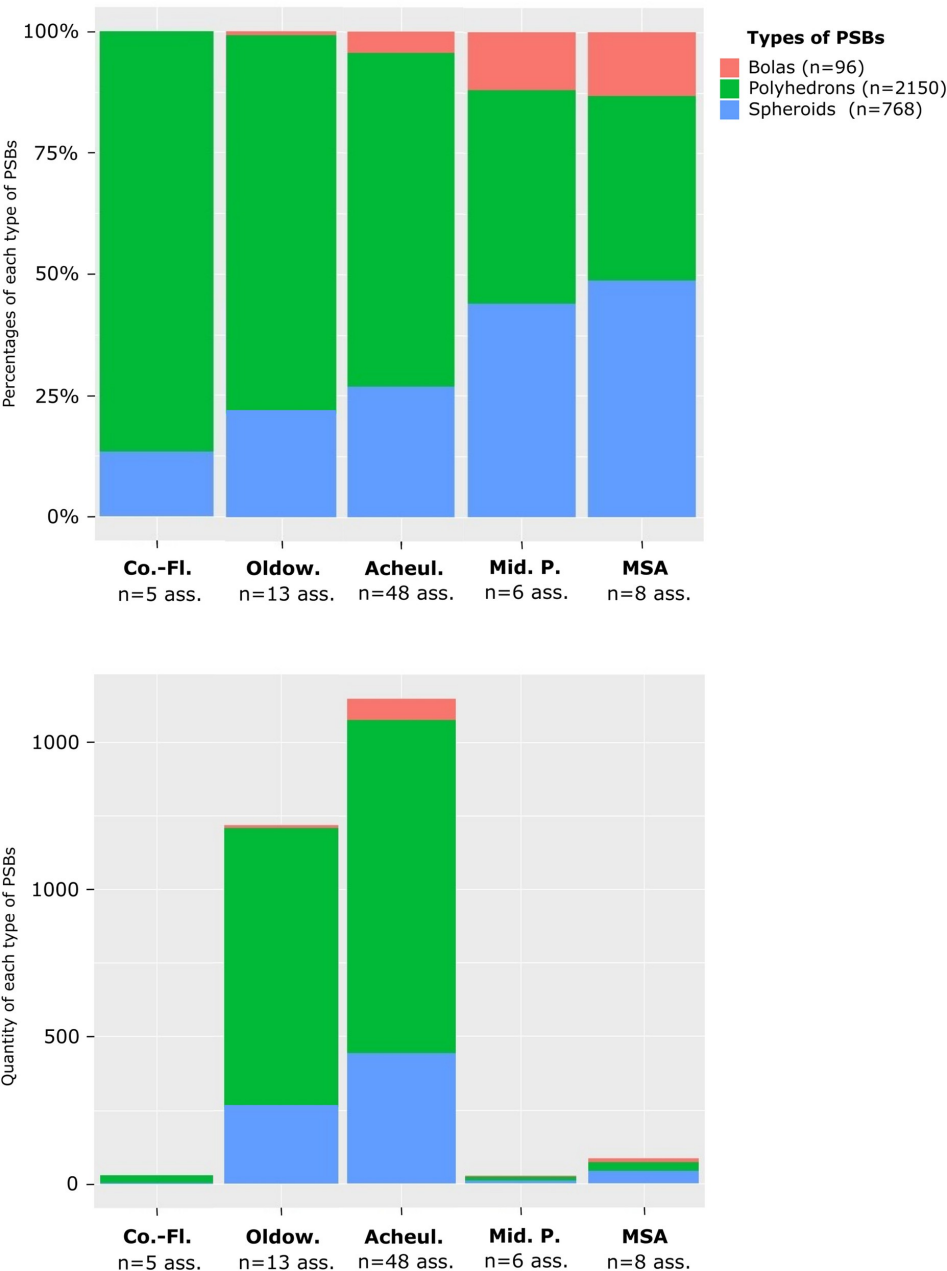
Frequencies and quantities of polyhedrons, spheroids and bolas in assemblages according to chrono-cultural attributions. Oldow.: Oldowan. Co.-Fl.: Core-and-Flake. Acheul.: Acheulian. Mid. P.: Middle Palaeolithic. Ass.: assemblages.

PSB morphologies appeared in Africa during the Oldowan, where they are frequent. Meanwhile, they are scarce in Eurasia and the Levant at slightly later Core-and-Flake-type sites (Tables 1 and 2, Fig 3). The production of these morphologies became more common outside Africa during the Acheulian (diffusion out of Africa? Technological convergence?), and tend to be scarcer again in the MSA and the Middle Palaeolithic (Table 1, Fig 3). Graphically, the frequencies of these objects in lithic assemblages are lower in European assemblages, Middle Palaeolithic, MSA and Core-and-Flake-type sites (Fig 5). In Asia, although our data are partial, we note that there are less Middle Palaeolithic sites with PSBs (n = 5 assemblages) than Early Palaeolithic sites (n = 10 assemblages). However, one Asian series attributed to the Middle Palaeolithic, Xujiayao (China) yielded more than a thousand PSBs, which strongly influences the mean for this period (Table 2), when PSBs are generally scarce. In the Levant, we did not identify any Middle Palaeolithic site with PSBs (Fig 4). Polyhedrons are way more common than more rounded forms (Fig 4), and are the only category of PSBs for which analyses of quantities provided significant results when only considering sites that yielded the type of PSB of interest (here, considering only assemblages with polyhedrons) (Table 1). Fig 6 shows a higher frequency of polyhedrons in early chrono-cultural complexes (Oldowan and Acheulian) than in later ones (Middle Palaeolithic and MSA), and on the contrary, higher frequencies of spheroids and bolas in more recent chrono-cultural complexes. This is a tendency at the scale of the cultural techno-complexes, not at the scale of the site: polyhedral and spherical pieces are present in all the techno-cultural complexes discussed here, but in various proportions, that seem to evolve with time. Over time, PSBs seem to become scarcer and more rounded (Fig 6). This could suggest a standardisation of these objects over time.

**Table 2:** Statistics regarding quantities of PSBs per site, according to regions and chrono-cultural attributions.

Chrono-cultural Attribution	Mean	Standard Deviation	Max.	Min.	Median	PSBs (N =)	Sites (N =)
**Oldowan**	61.4	122.15	457	1	12	1228	20
**Core-and-Flake**	4.29	3.90	12	1	3	30	7
**Acheulian**	21.27	40.46	257	1	9	1702	80
**MSA**	13.1	16.04	46	1	4	131	10
**Mid. Paleo.**	129.11	354.45	1073	1	4	1162	9
**Region**	**Mean**	**Standard Deviation**	**Max.**	**Min.**	**Median**	**PSBs (N =)**	**Sites (N =)**
**Central Africa**	3	1.73	4	1	4	9	3
**East Africa**	31.3	61.08	345	1	8	1252	40
**North Africa**	100.83	175.95	457	4	36.5	605	6
**South Africa**	27.35	46.26	177	1	11	465	17
**West Africa**	22	*NA*	22	22	22	22	1
**East Asia**	71.81	267.02	1073	1	3	1149	16
**South Asia**	28.6	49.53	117	1	9	143	5
**Europe**	6.82	10.49	53	1	3	191	28
**Levant**	21.92	50.72	257	2	10	570	26

### Types of raw materials of PSBs

PSBs made on hard stones represent the vast majority of the PSBs of the corpus (Table 3). The hardness of a stone is based on its response to a physical constraint: on one hand, the ability to withstand moderate shocks by transfer of energy between grains or inside the matrix, and on the other hand, the ability to facilitate the breakage or the burst. The silicifications (e.g., chert, flint, millstone) will fragment more easily, contrary to more resistant raw materials. This is more related to the way the silica crystallised than to the content of silica of the stone, with on one hand amorphous silica or chalcedony for flint and chert, and on the other hand, when there is some, a siliceous cement consolidating crystals or minerals. There is also a part of qualitative appreciation based on the knapping experience. Many PSBs are also made from quartz. All the following counts of assemblages are minimum since data may be partial:

- Limestone (mostly hard types): 32.38% of PSBs (n = 600/1853), in n = 18/150 assemblages.
- Quartzite: 18.24% of PSBs (n = 338/1853), in n = 41/150 assemblages.
- Quartz: 11.87% of PSBs (n = 220/1853), in n = 62/151 assemblages.
- Phonolite: 10.90% of PSBs (n = 202/1853), in n = 4/150 assemblages (remark: 3 of these 4 assemblages are from the site of Isenya, Kenya).
- Andesite: 9.93% of PSBs (n = 184/1853), in n = 15/150 assemblages (remark: 13 of these 15 assemblages are from Kaletepe Deresi 3 site, Turkey).
- Basalt: 7.39% of PSSBs (n = 137/1853), in n = 32/150 assemblages.

**Table 3:** Minimum counts of assemblages with at least one PSB in the raw material of interest, and PSB frequencies according to raw materials and regions.

	Africa		Asia		Europe		Levant	
Raw materials	Number assemb.	Freq. PSBs	Number assemb.	Freq. PSBs	Number assemb.	Freq. PSBs	Number assemb.	Freq. PSBs
Limestone	4	**38.27**	1	*NA*	4	**10.06**	**9**	**23.11**
Sandstone	7	2.70	4	6.56	2	0.59	0	0
Chert	1	0.07	0	0	0	0	2	0
Flint	2	2.70	0	0	5	4.14	3	1.59
Breccia	1	0.07	0	0	0	0	0	0
Ironstone	1	0.29	1	*NA*	1	*NA*	1	*NA*
Basalt	**25**	**8.75**	3	1.64	1	**7.69**	3	1.20
Andesite	0	0	1	*NA*	0	0	**14**	**73.31**
Phonolite	4	**14.72**	0	0	0	0	0	0
Diabase	4	4.08	0	0	0	0	0	0
Nephelinite	1	0.15	0	0	0	0	0	0
Obsidian	1	*NA*	0	0	0	0	0	0
Trachyte	1	*NA*	0	0	0	0	0	0
Granite	1	0.51	1	4.92	0	0	0	0
Igneous und.	**15**	0.15	1	3.28	0	0	0	0
Quartz	**37**	**12.17**	**11**	**22.95**	**13**	**21.89**	1	0.80
Dolomite	0	0	1	*NA*	0	0	0	0
Quartzite	**21**	**15.16**	**11**	**59.02**	**8**	**55.62**	0	0
Silicified und.	0	0	2	1.64	0	0	0	0
Cataclasite	1	0.22	0	0	0	0	0	0
**TOTAL PSBs**	-	1372	-	61	-	169	-	251
**TOTAL assemb.**	71	35	23	10	29	23	27	21

Number assemb.: counts of assemblages with at least one PSB in the raw material of interest per region. Freq. PSBs: percentages of PSBs in assemblages according to regions. TOTAL assemb.: total number of assemblages considered per region (for example in Africa: qualitative data about the presence/absence of PSBs in the different raw material were available for 71 assemblages, and quantitative data were available for 35 of these assemblages). Igneous und.: unspecified igneous rock. Siliceous und.: unspecified siliceous rocks. Raw materials in red: sedimentary rocks; in blue: igneous rocks; in light green: mineral rocks; in dark green: metamorphic rock. Remark: the total sum of each column “Number assemb.” is higher than the associated “TOTAL assemb.” because some assemblages yielded PSBs in various raw materials, and are thus considered in several lines. For example, the total sum of the column for Africa is 127, but it represents 71 assemblages.

**Fig 7:**
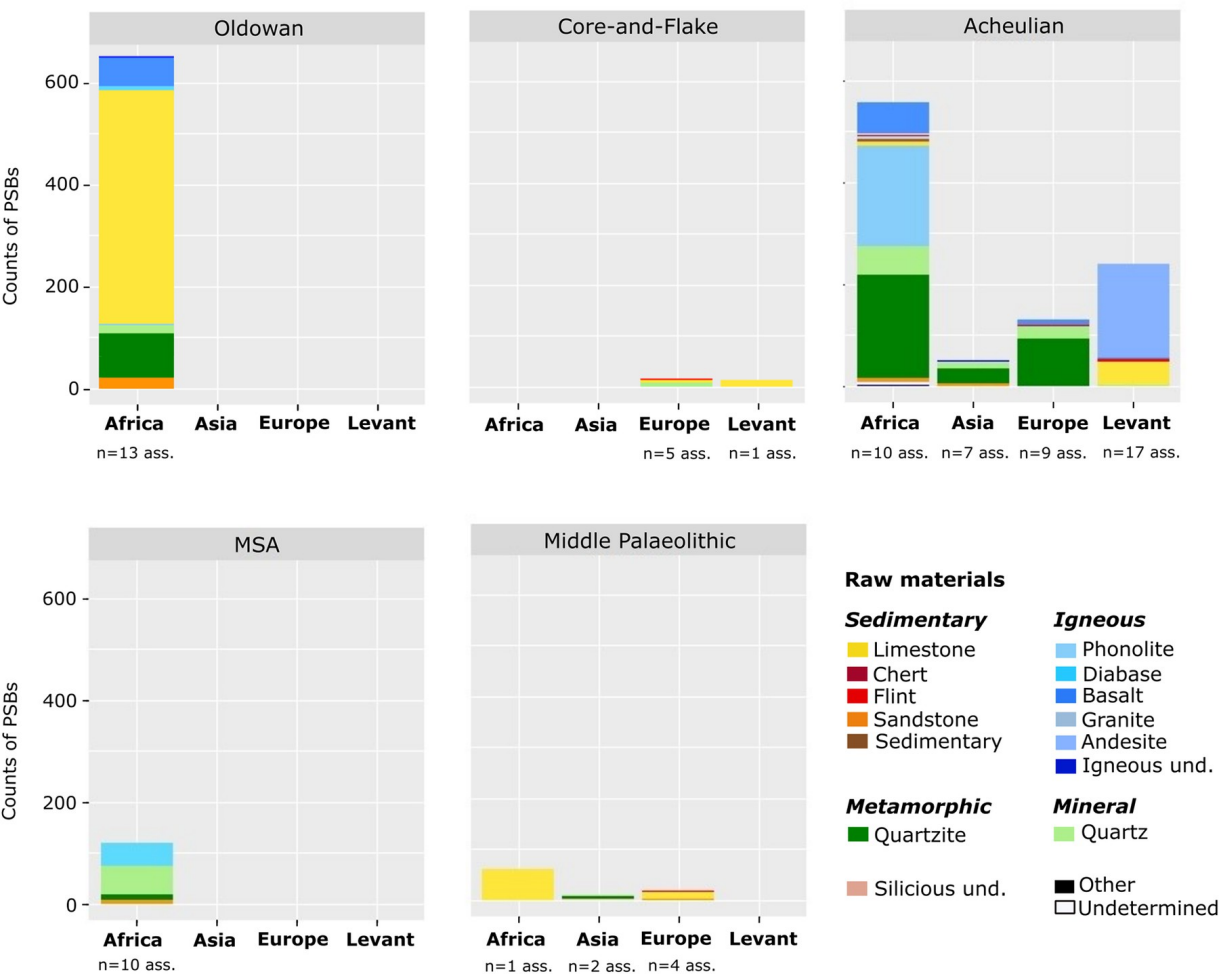
Counts of PSBs in each raw material per assemblage, per region and chrono-cultural attribution. For more details about counts: see Tables 3 and 4. Igneous und.: unspecified igneous rock. Siliceous und.: unspecified siliceous rocks. Sedimentary: breccia and ironstone. Ass.: assemblages.

**Fig 8:**
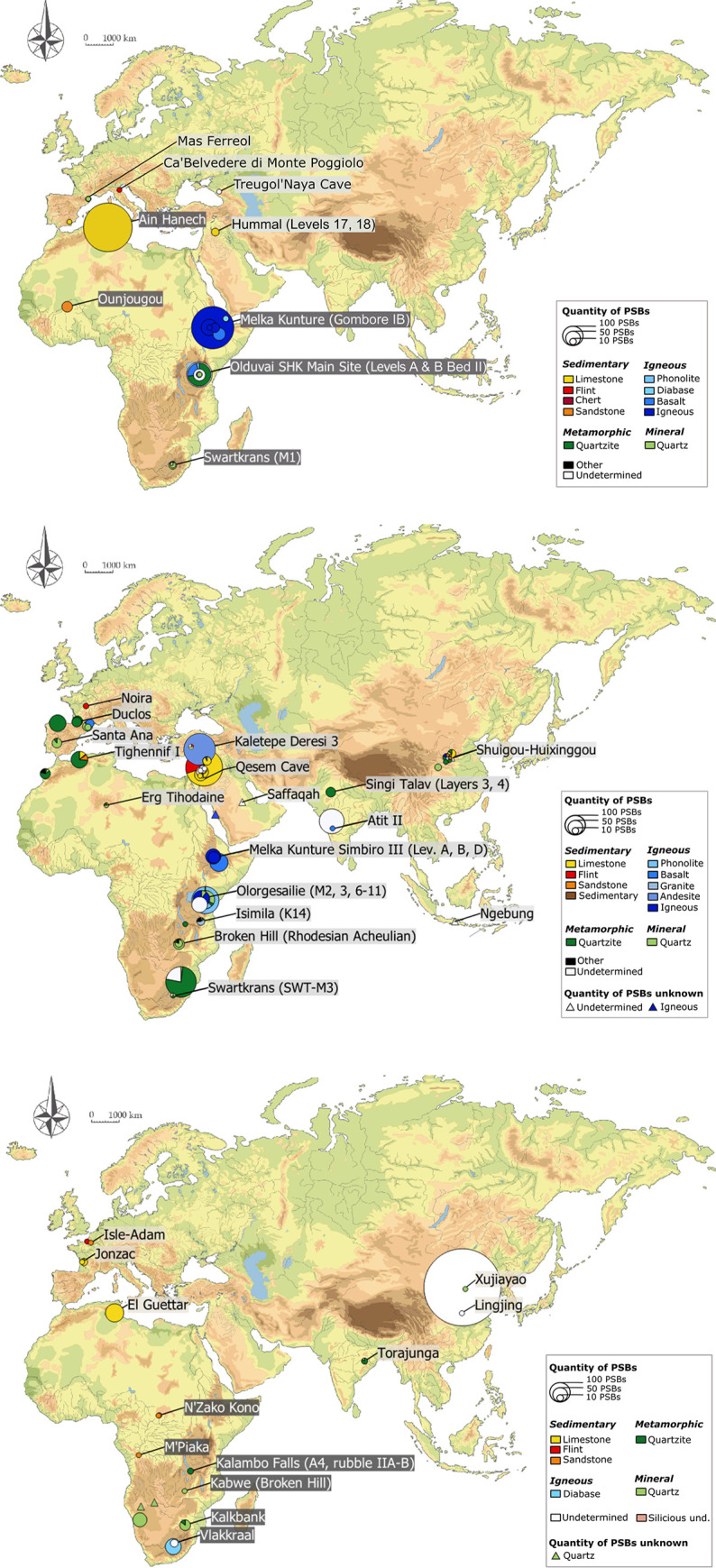
Raw materials of PSBs in Africa, the Levant and Eurasia. (A) Oldowan (dark-grey site labels) and Core-and-Flake-type industry (light-grey site labels). (B) Acheulian. (C) MSA (dark-grey site labels) and Middle Palaeolithic (light-grey site labels). Siliceous und.: unspecified siliceous rock. Igneous: mainly igneous rocks for which quantities were unspecified in the literature, including mostly basalt. Sedimentary: breccia and ironstone. For a few assemblages, the proportions represented are higher than in tables for igneous and sedimentary rocks: raw materials were in a few cases grouped here in more general categories to show quantities on the map. For example: Melka Kunturé Garba IV yielded PSBs in volcanic rocks including basalt, but maybe also trachyte or tuff according to d’Andrea et al. [80]. Since we have the total numbers of PSBs in this assemblage, but not the exact proportions for each raw material, this assemblage is not considered in “Freq. PSB” of Tables 3 and 4. However for the map, raw materials of these PSBs were grouped under “Igneous” in order to show information in the figure. The list of these changes and approximations is available in S2 Text.

PSBs are rarely made of siliceous materials such as flint or chert (Table 3, Figs 7 and 8). Among the 169 assemblages, only three assemblages yielded PSBs in chert: 1 polyhedron at Swartkrans SWT-M2 in South-Africa (n = 1/12 PSB of this assemblage) [59], and both ‘Ubeidiya III-20 and III-22 present polyhedrons in chert and fine-grained limestone (while spheroids are in limestone) [6, 60–62]. At least ten assemblages yielded PSBs in flint (n = 48/1853 PSBs are in flint), including five European assemblages, three from the Levant and two from Africa. More precisely, sites with only flint PSBs (n = 3 assemblages, minimum) are exclusively located in Europe, and they comprise only few PSBs (one to four PSBs), and the rest of these assemblages are totally (or nearly) made of siliceous materials. This could mean that siliceous rocks were not selected or not suitable for the production of PSBs. Furthermore, siliceous stones are predominant in Northwest European basins, where PSBs are rare. Could this scarcity of PSBs be linked to a Northwest European tradition? Furthermore, can this tradition be partly correlated to this abundance of siliceous materials, which could somehow have influenced lithic production? In this case, this availability of siliceous materials would have impacted technological traditions, resulting in a toolkit without PSBs. In some sites, PSBs are the only non-flint items of the series, as at Qesem Cave [12]. In total, out of the 33 assemblages (some of which are part of a same site, at a different period) of the corpus that yielded lithic artefacts in flint, we observe:

- Eighteen assemblages with no PSBs in flint. In five of these 18 assemblages, PSBs are the only limestone pieces among series of artefacts almost exclusively made of flint, as at El Guettar, Tunisia [24], Revadim Quarry, Israel [63], Evron Quarry, Israel [64], or in US 22 of Jonzac, France [65]. At La Quina, France, PSBs are the only Turonian limestone pieces in an assemblage made mostly of Coniacian flint and quartz, with a few objects in chalcedony. The only flakes in limestone here are in Coniacian limestone and PSBs are not concerned. In the 13/18 other assemblages, PSBs are not made from siliceous materials in assemblages including flint artefacts. For instance, at Ain Hanech, PSBs are all made on limestone whereas the rest of the lithics are mostly in very good quality black flint and limestone [39, 46]. At North of Bridge Acheulian (NBA), a locality near Gesher Benot Ya’aqov site, Israel, six spheroids are made in limestone and three in basalt (the raw material of the three pieces described as “sub-spheroids” is not given), and the rest of the lithic assemblage is in basalt and flint [66]. At Latamné, Syria, spheroids (n = 14) are in limestone except one in basalt, the only basalt piece of the assemblage. Spheroids are among the rare limestone objects (n = 74 items in limestone in the whole assemblage) out of 1759 pieces in flint [67, 68]. At Barranco León, Spain, PSBs are in limestone while the rest of the lithic assemblage is in flint and limestone [13]. The Caune de l’Arago, France (Units D, E, G, H1-3) and Zhoukoudian 15, China, yielded PSBs nearly exclusively made in quartz in series with pieces in flint [69, 70]. At Duclos, France, and Bañugues, Spain, PSBs are in quartzite, as are most of the rest of the assemblages, that also include pieces in flint [71, 72].
- Two assemblages yielded a very small minority of PSBs in flint: Qesem Cave, Israel, with one spheroid in flint (different from the flint of the rest of the assemblage) and the 28 other PSBs are the only pieces made from hard limestone, among tens of thousands of artefacts in flint [12]. According to Assaf et al. [12], these PSBs were not manufactured in the cave but rather recycled by hominins who found it already shaped in the vicinity of the site, and brought it to the cave to be used in bone breakage to extract the marrow [12]. Tighennif I, Algeria, also yielded only one polyhedron in flint associated with 52 PSBs in other raw materials (39 in quartzite, six in sandstone and seven in limestone) [73].
- Four assemblages yielded PSBs in flint and in other raw materials in approximately the same proportions: Sterkfontein Member 5, South Africa [17], Joubb Jannine II, Lebanon [74] and Festons, France [26, 75], where the flint PSBs were the least rounded pieces of the assemblages; and Sablière Rambour, France [76], where on the contrary, polyhedrons in flint are more regular than the PSBs in sandstone.
- Three European sites where PSBs are only made on flint, and in very low quantities: Ca’Belvedere di Monte Poggiolo, Italy, with two polyhedrons and 93.24% of the assemblage made of local flint [13, 77]; stratum C of La Noira, France, with one polyhedron and the rest of the assemblage mainly in millstone (siliceous stone) and some pieces in flint, chert, quartz, sandstone and quartzite [78]; and finally Tourville, France, with four PSBs in an assemblage in local flint [79].
- Finally, five assemblages where there are items in flint but the raw material of PSBs is not given.

As we can see on the maps (Fig 8) there are similarities in PSB raw materials between relatively geographically close assemblages. In particular, we note that quartzite is mostly used in the region of the Gibraltar Strait during the Acheulian, on both the North African (n = 53/77, 68.83% of North African Acheulian PSBs, 3/3 assemblages) and Southwest European sides (Fig 8). PSBs in limestone are also very common in North Africa (n = 525/601, 87.35% of North African PSBs, n = 4/6 assemblages) and the Levant (n = 58/251, 23.11% of the Levantine PSBs, n = 9/27 assemblages), and very prevalent in Southwest Europe as well (Fig 8). PSBs in quartz are widely dominant in South Africa (n = 132/273, 48.35% of South African PSBs, n = 15/20 assemblages), and PSBs made of igneous rocks are the most common in East Africa (n = 339/467, 72.59% of East African PSBs, n = 35/43 assemblages) (Fig 8). In Central and West Africa, PSBs are rare but nearly exclusively made of sandstone (n = 29/31, 93.55% of Central and West African PSBs, n = 4/4 assemblages).

PSBs in limestone are dominant in Core-and-Flake assemblages (n = 17/27, 62.96% of Core-and-Flake PSBs, n = 2/7 assemblages) in Europe and the Levant. In terms of quantity, 71.07% of the Oldowan PSBs (n = 457/643 Oldowan PSBs) of the corpus are in limestone, but they only come from one North African assemblage, Ain Hanech. They are also quite numerous in Acheulian sites from the Levant (n = 46/239, 19.25% of Acheulian Levantine PSBs, n = 8/26 assemblages). Finally, limestone PSBs are also predominant in Middle Palaeolithic assemblages in Europe (n = 12/18, 66.67% of Middle Palaeolithic European PSBs, n = 2/4 assemblages) and at El Guettar, Tunisia.

PSBs in quartz are present in Acheulian assemblages from Africa (n = 57/419, 13.60% of African Acheulian PSBs, n = 20/33 assemblages) and Asia (n = 11/50, 22% of PSBs the Asian Acheulian PSBs, n = 4/9 assemblages), as well as in MSA sites (n = 55/120, 45.83% of PSBs in the MSA sites, n = 6/13 assemblages), especially in South Africa. They are also quite present at the Oldowan in terms of the number of assemblages that yielded at least one PSB in quartz (n = 8/21 sites).

Quartzite is also one of the main raw materials used to manufacture PSBs in the Oldowan assemblages of the corpus (n = 86/653, 13.17% of Oldowan PSBs, n = 7/21 assemblages), however it largely comes from assemblages from Olduvai Gorge. PSBs in quartzite are also quite common in the Acheulian sites from Africa (n = 68/419, 16.23% of Acheulian African PSBs, n = 9/33 assemblages), Europe (n = 93/131, 70.99% of Acheulian European PSBs, n = 8/13 assemblages) and Asia (n = 29/50, 58% of Acheulian Asian PSBs, n = 6/9 assemblages). They are also present in MSA assemblages (n = 12/120, 10.00% of MSA PSBs, n = 4/13 assemblages) and Asian Middle Palaeolithic sites (n = 6/9, 66.67% of Middle Palaeolithic Asian PSBs, n = 2/4 assemblages).

Finally, hard igneous rocks (particularly basalt) are often used for the production of PSBs, especially in Africa during the Oldowan (n = 71/653, 10.87% of Oldowan PSBs, n = 18/21 assemblages) and the Acheulian (n = 269/419, 64.20% of Acheulian African assemblages, n = 23/33 assemblages). This is also the case for Levantine Acheulian sites (n = 187/239, 78.24% of the Levantine Acheulian PSBs, n = 17/26 assemblages), but this high proportion is due to Kaletepe Deresi 3 site, which yielded 13 assemblages with PSBs all made of andesite. According to our data, PSBs in igneous rocks are nearly absent during the Middle Palaeolithic and MSA (Table 4). Indeed, as we saw above, over both of these recent periods, PSBs were scarcer and tended to be more rounded, compared to sites from earliest techno-complexes.

**Table 4:** Minimum numbers of assemblages with at least one PSB in the raw material of interest, and frequencies of PSBs according to raw materials and chrono-cultural attributions.

Raw Material	Oldowan		Core-and-Flake		Acheulian		MSA		Middle Palaeolithic	
	Number assemb.	Freq. PSBs	Number assemb.	Freq. PSBs	Number assemb.	Freq. PSBs	Number assemb.	Freq. PSBs	Number assemb.	Freq. PSBs
Limestone	1	**69.98**	2	**62.96**	**11**	**6.44**	0	0	**3**	**82.76**
Sandstone	1	3.37	0	0	6	1.43	**3**	**9.46**	1	1.15
Chert	1	0.15	0	0	2	*NA*	0	0	0	0
Flint	0	0	1	7.41	5	0.72	0	0	1	4.60
Breccia	0	0	0	0	1	0.12	0	0	0	0
Ironstone	0	0	0	0	1	0.48	0	0	0	0
Basalte	**10**	**8.73**	0	0	**20**	**9.42**	0	0	0	0
Andesite	0	0	0	0	14	**21.93**	0	0	0	0
Phonolite	1	0.61	0	0	3	**23.60**	0	0	0	0
Diabase	2	1.23	0	0	0	0	1	38.33	0	0
Nephelinite	1	0.31	0	0	0	0	0	0	0	0
Granite	0	0	0	0	2	1.19	0	0	0	0
Igneous und.	**5**	*NA*	0	0	**13**	0.48	0	0	0	0
Quartz	**8**	2.45	**4**	**29.63**	**28**	**11.20**	**6**	**45.84**	2	3.45
Dolomite	0	0	0	0	0	**0**	0	0	1	*NA*
Quartzite	6	**13.17**	0	0	**22**	**22.65**	**4**	**10.00**	1	6.90
Siliceous und.	0	0	0	0	1	*NA*	0	0	1	1.15
Cataclasite	0	0	0	0	1	0.36	0	0	0	0
**TOTAL PSBs**	-	643	-	27	-	839	-	120	-	87
**TOTAL assem.**	21	13	7	6	80	49	13	9	9	7

Number assemb.: counts of assemblages with at least one PSB in the raw material of interest per chrono-cultural attributions. Freq. PSBs: percentages of PSBs in assemblages according to chrono-cultural attributions. TOTAL assemb.: total number of assemblages considered per chrono-cultural attributions (for example for the Oldowan: qualitative data about the presence/absence of PSBs in the different raw material were available for 21 assemblages, and quantitative data were available for 13 of these assemblages). Igneous und.: unspecified igneous rock. Siliceous und.: unspecified siliceous rocks. Raw materials in red: sedimentary rocks; in blue: igneous rocks; in light green: mineral rocks; in dark green: metamorphic rock. Remark: the total sum of each column “Number assemb.” is higher than the associated “TOTAL assem.” because some assemblages yielded PSBs in various raw materials, and thu s are considered in several lines. For example, the total sum of the column for the Oldowan is 36, but it represents 21 assemblages.

**Fig 9:**
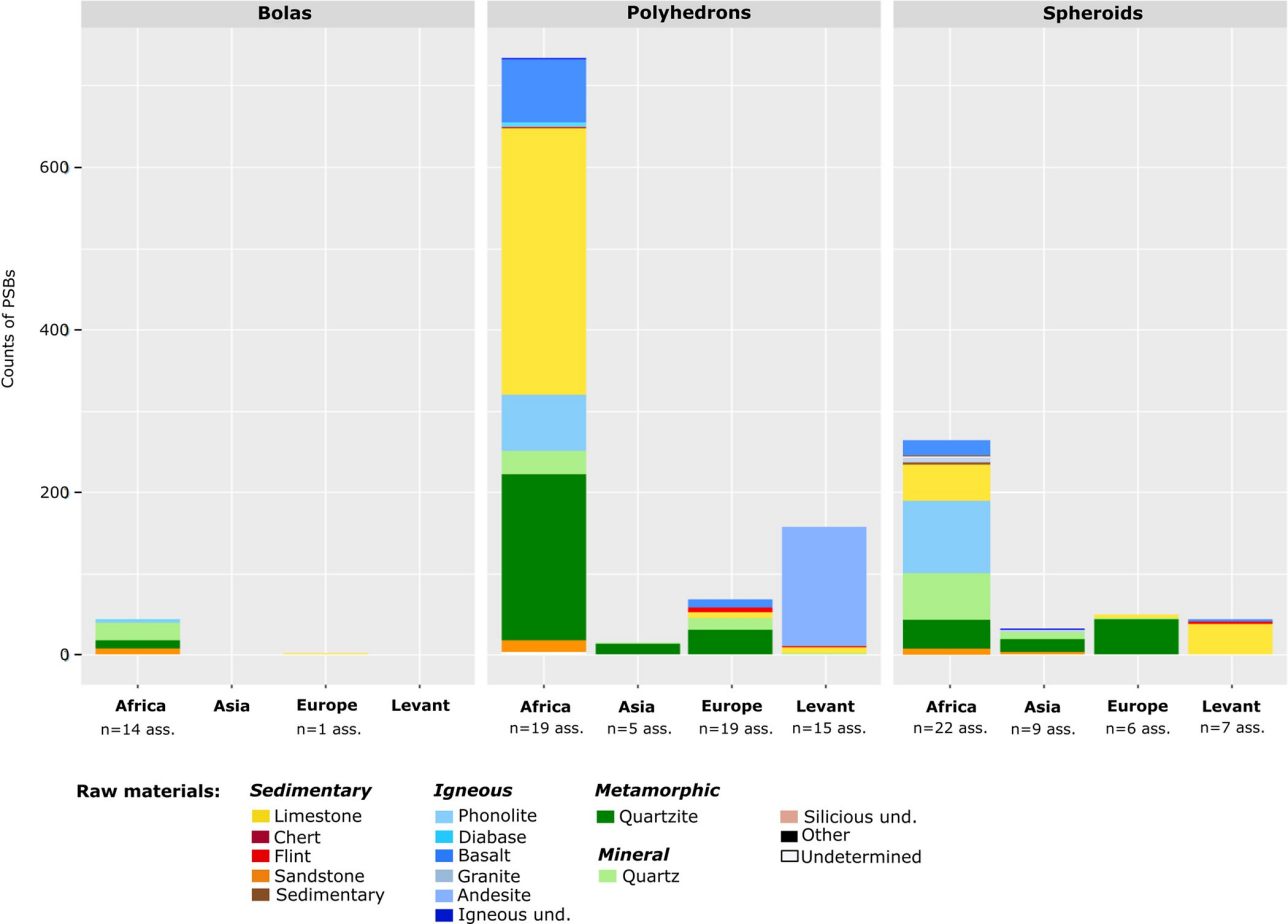
Counts of polyhedrons, spheroids and bolas per raw material in assemblages, in each region. Igneous und.: unspecified igneous rock. Siliceous und.: unspecified siliceous rocks. Sedimentary: breccia and ironstone.

**Fig 10:**
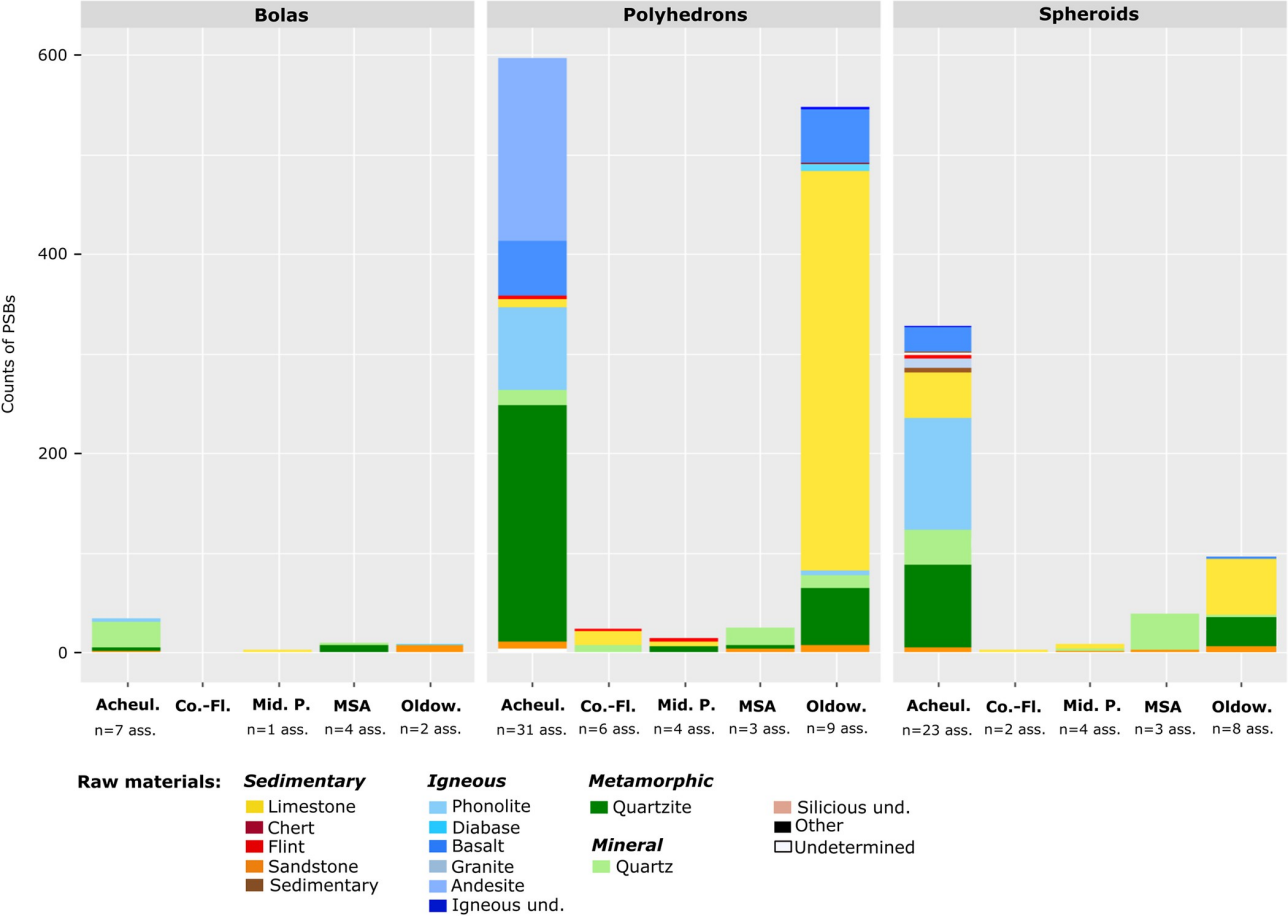
Counts of polyhedrons, spheroids and bolas in each raw material per assemblage, according to chrono-cultural attribution. Igneous und.: unspecified igneous rock. Siliceous und.: unspecified siliceous rocks. Sedimentary: breccia and ironstone. Oldow.: Oldowan. Co.-Fl.: Core-and-Flake. Acheul.: Acheulian. Mid. P.: Middle Palaeolithic. Ass.: assemblages.

Polyhedrons, spheroids and bolas can respectively be made of different raw materials (Figs 9 and 10). On graphs (Figs 9 and 10), the height of columns depends more on the amount of available data in the literature than on the real quantity of PSBs. Polyhedrons tend to be made in igneous rocks more frequently than spheroids and bolas. On the contrary, spheroids and particularly bolas are more often made in quartz than polyhedrons (Figs 9 and 10). Furthermore, in a same assemblage, spheroids and/or bolas can be in quartz while polyhedrons are in other raw materials. Thus, generally, the more rounded the PSBs, the more they tend to be made on quartz, especially in African and Asian assemblages.

Generally, sources of the raw materials used to make PSBs are local, in the same way as the other raw materials of the lithic assemblage. For instance, the tools from Erg Tihodaïne are made on local stones, from 0 to 5 km away [81]. At Isenya, most PSBs are made from local phonolite of Kapiti, and the other materials (quartz, quartzite, nephelinite) are available less than 7 km away [82, 83]. There are however some exceptions. For instance, at Olorgesailie (Kenya), PSBs are mostly in basalt or other types of lava, whereas rare large spheroids are in quartz imported from northern Tanzania, 43.48 km away [84]. This special attention for quartz could point to the hypothesis of a special selection of quartz for the production of PSBs at this site.

In some cases, PSB raw materials are different from the rest of the lithic assemblage, suggesting a selection by hominins of specific stones for these pieces. For instance, we can mention the Clay and SC Units of Olduvai HWK-EE, Tanzania, where items described as “sub-spheroids” are the only PSBs exclusively in quartzite [85], Levels A and B of Olduvai SHK Main Site where spheroids are the only type of objects made exclusively from quartzite [21], Vlakkraal Thermal Springs, South Africa, where PSBs are the only pieces not made from indurated shale [86], El Guettar, Tunisia, with a large majority of lithics in flint but PSBs in limestone [24], Dingcun, China, where 94.6% of the assemblage is made from fine-grained hornfels while PSBs are mainly in limestone and in quartz, green sandstone and quartzite [42], or many Levantine sites where PSBs are the only items in hard limestone among more fragile flint lithics. When PSBs are not the only objects of the series in a specific raw material, they are often made from the same stones used to make the other HDTs of the assemblage (more likely than LDTs or cores), still in different proportions. According to our data, PSBs are more rarely made from the same material as hammers. This may be due to a bias in the available information, as hammerstone raw materials are rarely mentioned. Even when PSBs are made from the same materials as other artefacts in the assemblage, they can sometimes be distinguished by the special morphology and size of the often-ovoid blank, suggesting selection by hominins (e.g., [13, 72]).

Information is often not specified in the literature for PSB blank type, (n = 88/169 assemblages without this information). When it is mentioned and possible to assess (for pieces with cortical patches), they are widely made from pebbles and cobbles (n = 58/79 assemblages). Most of the assemblages with PSBs made on slabs (n = 13/15 assemblages) are from Kaletepe Deresi 3 site, Turkey, attributed to the Acheulian chrono-complex. Few sites yielded PSBs made on blocks, mostly in Africa (n = 8/30 assemblages), and very rarely on big flakes (n = 2 assemblages).

### Functions of sites with PSBs

Occupation types and the activities carried out on sites could be completely or partially documented for 57 assemblages of the corpus. Butchery activities are the most frequent (n = 33/57 assemblages minimum), but this is also the case in sites without PSBs. Butchery activities are also more frequently recorded as they are easier to identify at first sight (e.g., presence of intentionally broken and fragmented bones, remains of carcasses). Use-wear analyses have not been performed for many sites, perhaps explaining why wood and plant processing are rarely documented in the sites of the corpus. Most of the sites are also described as habitats or settlements of various durations. At least 12 sites indicate knapping activities and three point to hunting.

### Environment and climate

Assemblages with PSBs do not reveal a specific general environmental context. They are located near a water source, generally with an arid to semi-arid climate. In most cases, the landscape was open with sparse vegetation, or forested, sometimes tropical humid, rarely swampy. However, these parameters may be too general to draw any conclusions.

### Composition of assemblages with PSBs

Generally, as for most Palaeolithic sites, assemblages with PSBs yielded big mammal remains. The absence of such remains is generally due to taphonomic processes, for instance high soil acidity that dissolved organic materials, as is the case at Ounjougou, Mali [11], or in the majority of sites from Rajasthan, India. When large faunal remains are present in the corpus, they are mainly intentionally anthropically broken bones, except in the Levant, possibly to extract marrow (Table 5). For example, at Isenya (level V and VI), Kenya, Roche et al. [82] suggest that bone breakage was probably anthropical, with broken long bone epiphyses and diaphysis, as is common in East African sites. Pante and de la Torre [87] also record intentional bone breakage for the extraction of marrow at the East African site Olduvai HWE-EE (SC and Clay Unit, Bed II). This is the case in European sites as well, for instance the Caune de l’Arago (D Unit) [88], Tourville [79] and Jonzac, France. The latter site may possibly have been a workshop for the primary treatment of carcasses [89]. At La Quina and Festons [26] in France, *in situ* fracturing of reindeer carcasses to extract marrow was also reported [90, 91]. Such occurrences can also be attested in China with for example, Hsuchiyao site [92], layer 11 and the lower part of layer 10 of the kill-butchery-site of Lingjing, [93], or the horse-kill site of Xujiayao [94], and finally, in the Levant with the example of Qesem Cave [12].

**Table 5:** Percentages and minimum numbers of assemblages with PSBs and intentionally broken bones, according to regions.

Region	No				Yes		*NA*		Total	N assemblage
	*Absent*		*Other*		N	%	N	%		
	N	%	N	%						
**Africa**	4	5.40%	2	2.70%	12	16.22%	*56*	*75*.*68%*	100.00%	74
**Asia**	2	5.71%	0	0%	3	8.57%	*30*	*85*.*71%*	100.00%	35
**Europe**	3	10%	2	6.67%	6	20.00%	*19*	*63*.*33%*	100.00%	30
**Levant**	3	10%	5	16.67%	1	3.33%	*21*	*70*.*00%*	100.00%	30
**TOTAL**	12	7.18%	9	5.39%	22	13.17%	*126*	*74*.*25%*	-	169

N: Number of assemblages per region. %: Percentage of assemblages per region. No: absence of intentionally broken bones. Absent: absence of bones. Other: bones are present but broken by carnivores or any other non-anthropic agents. Yes: presence of intentionally broken bones. *NA*: no information about the presence or absence of intentionally broken bones.

At *in-situ* assemblages, PSBs can be spatially associated with intentionally broken bones, for instance at the Oldowan site of Barogali, Republic of Djibouti, where an elephant carcass was discovered in a wetland surrounded by PSBs and other lithic artefacts [95]. Barogali yielded one bola and six polyhedrons, all in diabase. The bola was found near the epiphysis of a big bone with the polyhedrons, associated with choppers, hammerstones and intentionally broken bones (ribs, epiphysis, vertebrae, big bones from limbs). At the Oldowan hippo butchery site of Gadeb 8F, Tanzania, according to Assefa et al. [96]: “scapula, long bone and rib fragments lay together with two sub-spheroids and a spheroid which had been used to break up the bones to get at the marrow”. In Ethiopia, Melka Kunturé Gombore II (Locality 2) yielded a polyhedron and two bolas discovered among bone fragments and near a basalt cleaver [97].

Types of debitage are not exhaustive in our database (n = 79/169 assemblages with no data at all). The rare data show that debitage is discoid (n = 55/90 assemblages), bipolar (n = 30/90 assemblages), unipolar (n = 28/90 assemblages), and bipolar on anvil (n = 18/90 assemblages). The available data do not provide evidence of PSB association with specific types of debitage. The series reflect the technological trends observed over time and space, regardless of the presence of PSBs.

Other types of artefacts are obviously discovered in assemblages with PSBs, as in most prehistoric lithic series: cores, flakes, debris, cobble tools (especially in East and South Africa, East and Southeast Asia, Europe and the Levant, but not so much in the MSA and the Middle Palaeolithic). PSBs are thus not isolated tools but are part of a large tool kit composed of heavy and light-duty tools. Scrapers, the most common flake tools, are also often present in East Africa, East Asia, Europe and the Levant, in Acheulian and Middle Palaeolithic periods. Hammerstones were often found associated with PSBs in the Acheulian, in European and East Asian sites, as well as in Africa in the Oldowan. Regarding LDTs, denticulates, notches and knives are also common in Acheulian assemblages with PSBs, in East Africa and the Levant. Points, notches and burins are present in Middle Palaeolithic sites. Unsurprisingly, bifacial industry (bifaces, handaxes, cleavers), as well as core-scrapers and picks are present in a large majority of Acheulian sites with PSBs, particularly in East Africa, followed by East Asia and the Levant. The presence of HDTs in assemblages with PSBs is less frequent in Europe. Interestingly, as PSBs, cleavers (in particular cleavers on flake) are very scarce in Northwest Europe, but present in the Southwest (e.g., Iberic Peninsula, Aquitaine Basin, Mediterranean Basin) [98, 99], where there is a higher diversity of raw materials and less siliceous rocks. European cleavers are generally made from hard materials (mostly quartzite), and are nearly absent in regions where populations preferred flint raw materials, as also underlined by Capdevielle [100]. This is a common point between cleavers and PSBs: they are lacking in Northwest Europe, where the tradition of production is mostly directed toward flint. Anvils are nearly exclusively mentioned in assemblages from East Africa (n = 13/43 East African assemblages, all from Olduvai and Melka Kunturé). Of the fifteen assemblages with anvils, only one, la Quina, also yielded bolas (n = 3 bolas). This apparent lack of anvils can also be related to recording methods in the field or data availability. Nonetheless, it contrasts with the hypothesis that PSBs were worked on an anvil, although this is still possible (especially for sites with anvils). Manuports are also very rarely mentioned.

## Discussion

This extensive survey and statistical review of Palaeolithic sites with PSBs provides insights into their distribution and context over time and space. These objects could represent a wide range of items displaying a final cubical to rounded shape, with a different history according to regions and periods. They tend to become more standardised with time. The first occurrences appear in Africa in the Oldowan, where they are relatively common, especially in East Africa, but also at Ain Hanech in North Africa, while they are scarce in Core-and-Flake sites in Europe and the Levant. Their production becomes more common outside Africa during the Acheulian, possibly with the diffusion of this chrono-cultural complex and/or technological convergences from human groups occupying Eurasia, before decreasing in the MSA and Middle Palaeolithic. Possible regional traditions, local evolution and reinvention of rounded morphologies can be assumed, particularly in Northwest Europe where they are very scarce. Siliceous raw materials have been excluded for the manufacture of these pieces. Siliceous rocks are abundant in Northwest Europe with a lithic production predominantly in flint, even if other materials were also available in the vicinity of sites. In this region, we also note the virtual absence of cleavers, widely made of quartzite and other hard stones, as are polyhedrons and spheroids. Thus, is it possible that a regional trend, influenced by the geological component of some European areas rich in siliceous material, resulted in a tool kit without PSBs nor cleavers? Indeed, a local evolution and/or adaptation could have led to the disappearance of these objects preferentially produced on hard raw materials. Their function could also have been transferred into another type of tool. It is indeed strange and original to observe in Northwest Europe the lack of typical African cleavers (made on a large flake) and few PSBs. Most of the cleavers are present in Europe in the South, for instance Spain and South of France. They are also present in Levantine assemblages. In the Northwest, hominins used mainly flint, because occupations are located in sedimentary basins rich in flint. In the South, the raw materials used are more diverse (flint but also quartz, quartzite for instance) due to the diversity of geological formations. Flint nodules can be shaped without producing large flakes, and we observe in the series bifacial tools with a transversal cutting edge. Are they similar to cleavers on flake? Or a different type? In the South, was it easier or necessary to produce large flakes to make a cleaver? For the PSBs, is there a relationship between main flint use and few PSBs, because flint was less suitable for the type of activities made by these tools?

The other parameters of site context studied here (functions of sites, environment, climate, types of debitage recognised on sites) did not allow to draw any conclusion. This is mainly due to the paucity of documentation available in the literature. It is true that butchery activities (including bone breakage to extract the marrow) are quite common in sites of the corpus, but it is the case for most of Palaeolithic sites regardless of the presence of PSBs. It is still an interesting avenue to be pursued with use-wear analysis on PSBs.

Raw materials seem to have been selected for the manufacture of PSBs, both in terms of stone type (hard, sometimes softer) and shape (mostly ovoid). There materials are predominantly local. The hardness of most of the raw materials could perhaps partly be explained by a functional purpose, for instance actions requiring a resistant object. Soft sedimentary rocks, such as some types of limestone, allowed for better management of the manufacture process [37] and were also selected. They could also allow striking platform angles to be slightly more obtuse. We also note similarities in PSB raw materials between sites located near each other, which is probably due to the local availability of these hard materials. It is also possible that this was the result of local traditions and/or adaptations of populations to new geological environments, with a choice of the most suitable materials. Furthermore, PSB raw materials are often similar to the stones used to make HDTs in the series, which can perhaps provide other clues about their possible functions (e.g., activities requiring a certain resistance to a shock, or to any physical constraint).

Proportionally, more rounded PSBs are more often made of quartz than angular ones. A first hypothesis is that hominins purposefully selected quartz to produce rounded items, maybe because of its tendency to become rounded by pecking or when battered. However, producing a PSB in quartz by hard percussion is quite risky and more complex than with many other raw materials. It seems unlikely that a PSB in quartz can be shaped by hard percussion without pre-conceptualisation. Finally, since this high frequency of PSBs in quartz (often milky white, translucent) occurs mainly during the MSA and Middle Palaeolithic, we cannot totally exclude the hypothesis that it could also be an aesthetic and/or symbolic choice. These non-functional arguments can also be advanced for the high proportions of spheroids and bolas during the MSA and Middle Palaeolithic, since PSBs tend to be closest to the perfect sphere during these techno-complexes. This higher proportion of more rounded pieces, particularly in quartz, in later chrono-cultural complexes could also be attributed to better manufacturing abilities, or to higher levels of curation (for instance as part of a transported tool-kit) that would have resulted in longer life-histories and increased roundedness. Indeed, the discussion of PSBs in later (post-Acheulian) sites is a matter of some debates. According to the cultural implications of PSBs defined by Mary Leakey [5], we may not deal with the same kinds of problematics in later sites, especially regarding spherical morphologies. In post-Acheulian sites, can PSBs be linked to the continuity of a morphology in relation to a potential functional interpretation? Use-wear analysis could help going deeper into this issue.

In a few cases, polyhedrons can be smaller and made in different materials to spheroids and bolas from a same assemblage. This led Jones [18] to argue that spheroids and bolas cannot be subsequent steps of polyhedron reduction in a unique operative chain. However, another hypothesis that we propose could be that in some cases, raw material properties partly conditioned the final morphology, tending to result for instance in a more rounded piece when made on quartz (e.g., more easily rounded by intentional pecking or during battering activities, ridges less sharp) and in a more angular object when on igneous rocks, even if the initial conceptual scheme could be similar. It is possible that igneous rocks may not be as suitable as quartz or fine-grained stones to produce (intentionally or not) rounded morphologies. Limestone being the most suitable materials to manage the production of PSBs, it could more easily result into diverse final shapes, from angular to perfectly rounded. Most of raw materials (including quartz and igneous rocks) can be blanks for polyhedral to rounded objects, but it will be a more or less complex and risky process according to the properties of the stone. This raises the issue of the predetermination in the degree of roundedness of some PSBs (cubical to rounded), and of the limits given by the raw materials (e.g., degree of hardness, of brittleness, homogeneity of the stone, shape of the blank) to produce a morphology, pre-determined or not. In some cases, polyhedrons could also have been smaller than the associated spheroids and bolas because they were precisely too small to pursue the reduction operative chain. Failures into the production process of a spheroid (e.g., error of the knapper, non-homogeneity of the stone) can lead to the breakage of the piece during the manufacture, and accidentally remove a large part of the object. After that, the production process can be reoriented and pursued, but can result in a less rounded (and smaller) piece. This could also explain why in some sites, some PSBs are much smaller than others, with sometimes a group of bigger PSBs and another group of much smaller ones: failures into the manufacture process, that unintentionally reduce the volume of the piece during the production.

In disagreement with Jones, some authors interpret polyhedrons, spheroids and bolas as three steps of a same operative chain, in order to obtain a final rounded object, for a specific task [8, 35]. Indeed, it is possible that these three types of objects are the three segments of a continuous operative chain. However, each of them can also be an end product in itself (intended or not). Bolas may not necessarily be the sought-after final product. Choppers could also be part of this operative chain, before the polyhedral step. Moreover, a same process (for instance: multidirectional removals on most of the surface of a pebble, where removal scars are striking platforms for the next removals), can sometimes lead to a polyhedral or spherical shape, depending on investment in the reduction process and how the volume is managed. Hypotheses on a conceptual and/or functional difference between polyhedrons and spheroids should be tested. However, one unique concept or scheme cannot be generalised to all sites. If different operative chains and various final shapes can exist for PSBs, the global morphology of polyhedrons and spheroids remains a round object with robust ridges thanks to obtuse angles. Different functions cannot be attributed to various polyhedrons and spheroids only because their manufacture mode and/or final shape differ (slightly or not).

As pebbles and cobbles are widely selected for making PSBs, a purposeful intention by hominins to reduce the operative chain has to be considered. On the other hand, the final rounded shape can also be a natural consequence of the use of pebbles and cobbles as blanks, without predetermination. However, cubical pebbles or cobbles may be more suitable since they display a first platform, as do large fragments or large flakes, for instance.

Furthermore, we observe only the final phase of these pieces before abandonment by hominins at the sites. Thus, it is possible that their status changed during manufacture and use, for instance from a core recycled as a percussion tool, if the raw material (hard enough?) and/or the preform (compact?) were suitable. If PSBs were tools, we can also wonder what would be the advantage of using PSBs instead of a simple pebble or cobble, considering the investment involved in their manufacture process. Are the robust ridges of polyhedrons and spheroids (thanks to obtuse angles) a sought-after advantage? Is the smoothness of cobble surfaces a drawback for gripping? Are PSBs opportunistic objects, cores (recycled or not)?

At this stage of the study, all the functional hypotheses have to be considered: e.g., percussion for subsistence or knapping, pounding, mashing of vegetal material, projectile, or core. To investigate these lingering questions about the potential functions of PSBs; predetermined or opportunistic objects, items that changed of status, and why they are nearly absent in Europe, comparative analyses of PSBs from Europe and Africa are ongoing. These pieces are compared in terms of manufacture and use modes, through experiments, technological and use-wear analyses. We intend to discuss their possible diffusion with hominin dispersals out of Africa, potential reinventions and regional trends. These rounded morphologies may have different meanings from one region and chrono-cultural complex to another, even from one site to another, certainly representing a wide variety of objects with the same shape. There may have been diffusions of these pieces through time and space, with adaptations and modifications, but also reinventions of the same morphologies in different localities and periods.

## Conclusion

The scarcity of PSBs in Northwest Europe can result from a combination of cultural and environmental factors: it could be due to a regional tradition, possibly influenced by this abundance of siliceous materials in the environment. In this region where the lithic production is widely turned toward the exploitation of flint, objects generally made from hard stones are scarce, resulting in a toolkit with only rare PSBs and cleavers. It is also possible that these missing objects were not useful anymore, or that their functions were transferred into another type of object. Flint have been avoided worldwide for the production of PSBs. Is it because flint was not suitable for the function of such objects?

Generally, hominins seem to have selected their raw materials to produce PSBs: hard stones, available locally. This could be related to a functional purpose, for instance for tasks requiring tools resistant to hard shocks or any other physical constraint. This is corroborated by the fact that materials of PSBs are often similar to the ones of HDTs in the associated assemblages. Soft sedimentary materials (e.g., some types of limestone) were also selected, as they allow a good management of the production process [36].

It is possible that in some cases, the properties of PSBs raw materials partly conditioned the final shape of these objects. For instance, angular pieces are more often made on igneous rocks than rounded ones, that are more often made on quartz. This could be because quartz is more likely to become rounded by pecking than other raw materials (manufacture or use process? Intentional or not?). This raises the question of the predetermination of the final shape of PSBs, and of the constraints and limits imposed by the raw material (e.g., degree of homogeneity, brittleness, morphology of the blank) in the production of a morphology, pre-determined or not. All types of stone and blanks do not have the same potential to produce a final rounded object.

PSBs may include a large variety of items with a global rounded shape, that may have been diffused, adapted but also widely reinvented over two million years across the old world. For now, no generalities can be made from one site to another regarding these objects. There may have been some technological convergence, particularly in Europe were these objects are scarce. Ongoing technological and functional analyses of PSBs from several sites will allow us developing the outcomes raised by this study.

## Supporting information

S1 Table Quantity of PSBs according to regions and cultures: Results of Kruskall-Wallis Effect Size tests. Method calculation: The effect size for Kruskall-Wallis test is computed as the eta squared based on the H-statistic: eta^2^[H] = (H-k+1) / (n-k); where H is the value obtained in the Kruskall-Wallis test; n the total number of observations and k the number of groups. Interpretation of the eta-squared estimate: 0.01–0.06 (small effect), 0.06–0.14 (moderate effect), > = 0.14 (large effect). Effsize: Estimate of the effect size. Magnitude: Magnitude of effect size. Empty dark grey cell: No result because the p-value of the Kruskall-Wallis test was >0.05.
(PDF)

S2 Table General data about the sites of the corpus. (PDF)

S3 Table Types of site of the corpus and their potential functions. (PDF)

S4 Table Types of environment and climate of the assemblages of the corpus. (PDF)

S5 Table Fauna recorded on the assemblages of the corpus. (PDF)

S6 Table Lithic items in the assemblages of the corpus. (PDF)

S1 Text Data about PSBs: Reference list, per region. (PDF)

S2 Text List of adjustments made for the construction of some pie charts in the Fig 8. (PDF)
