# Supplementary material for: Palaeolithic polyhedrons, spheroids and bolas over time and space
Source: PLoS One. 2022 Jul 28;17(7):e0272135. doi: 10.1371/journal.pone.0272135 (PMC9333226; doi:10.1371/journal.pone.0272135)
Supplement: S1 Table — Method calculation: The effect size for Kruskall-Wallis test is computed as the eta squared based on the H-statistic: eta2[H] = (H-k+1) / (n-k); where H is the value obtained in the Kruskall-Wallis test; n the total number of observations and k the number of groups. Interpretation of the eta-squared estimate: 0.01–0.06 (small effect), 0.06–0.14 (moderate effect), > = 0.14 (large effect). Effsize: Estimate of the effect size. Magnitude: Magnitude of effect size. Empty dark grey cell: No result because the p-value of the Kruskall-Wallis test was >0.05. (PDF) [file pone.0272135.s001.pdf]

S1 Table. Quantity of PSBs according to regions and cultures: results of Kruskall-Wallis Effect Size tests.

Criteria	PSBs	Polyhedrons		Spheroids		Bolas	
	All assemblages	All assemblages	Assemblages with polyhedrons	All assemblages	Assemblages with spheroids	All assemblages	Assemblages with bolas
**Large geographical areas** (Africa, Asia, Europe, Levant)	n=142 assemblages<br>Effsize=0.0958<br>Magnitude: **moderate**	n=137 assemblages<br>Effsize=0.0662<br>Magnitude: **moderate**	n=86 assemblages<br>Effsize=0.1900<br>Magnitude: **large**	n=140 assemblages<br>Effsize=0.0859<br>Magnitude: **moderate**	n=72 assemblages<br>Effsize=0.0715<br>Magnitude: **moderate**	n=154 assemblages<br>Effsize=0.0669<br>Magnitude: **moderate**	
**Sub-geographical regions** (East Africa, West Africa, Central Africa, North Africa, South Africa, East Asia, South Asia, South-East Asia, Europe, Levant)	n=142 assemblages<br>Effsize=0.1200<br>Magniture: **moderate**	n=137 assemblages<br>Effsize=0.148<br>Magnitude: **large**	n=86 assemblages<br>Effsize=0.2030<br>Magnitude: **large**		n=72 assemblages<br>Effsize=0.0747<br>Magnitude: **moderate**	n=154 assemblages<br>Effsize=0.1710<br>Magnitude: **large**	
**Cultural attributions** (Oldowan, Core-and-Flake-type, Acheulian, MSA, Middle Palaeolithic)		n=122 assemblages<br>Effsize=0.0456<br>Magnitude: **small**	n=80 assemblages<br>Effsize=0.1130<br>Magnitude: **moderate**		n=63 assemblages<br>Effsize=0.0947<br>Magnitude: **moderate**	n=132 assemblages<br>Effsize=0.0456<br>Magnitude: **small**	

Method calculation: the effect size for Kruskall-Wallis test is computed as the eta squared based on the H-statistic: $eta^2[H]= (H-k+1) / (n-k)$; where H is the value obtained in the Kruskall-Wallis test; n the total number of observations and k the number of groups. Interpretation of the eta-squared estimate: 0.01 - 0.06 (small effect), 0.06 - 0.14 (moderate effect), >=0.14 (large effect). Effsize: estimate of the effect size. Magnitude: magnitude of effect size. Empty dark grey cell: no result because the p-value of the Kruskall-Wallis test was >0.05.
