# Supplementary material for: Palaeolithic polyhedrons, spheroids and bolas over time and space
Source: PLoS One. 2022 Jul 28;17(7):e0272135. doi: 10.1371/journal.pone.0272135 (PMC9333226; doi:10.1371/journal.pone.0272135)
Supplement: S2 Table — (PDF) [file pone.0272135.s002.pdf]

S2 Table. General data about the sites of the corpus.

Key of reading:

Names of assemblage in dark grey cells: assemblages for which we considered some objects as PSBs but most of authors who worked on the site did not (e.g., they could consider it as multifacial cores).

Light grey cells: the information in the cell is very probable but not certain, or is incomplete.

Excavation dates in bold: campaigns of excavation that yielded the PSBs considered in the study. When there is no date in bold in a cell, we do not know which campaign yielded the PSBs.

NA: no information collected.

Site	Country	Age	Comments - Date, dating methods	Cultural attribution	Excavation dates
**Ewass Oldupa (Phase II)**	Tanzania	2.015 Ma	Age of the Phase II, uppermost part of a tuff: 2.015 +/- 0.006 Ma (Deino 2012 [1], Cueva-Temprana et al. 2022 [2]).	Oldowan	NA
**Ewass Oldupa (Phase III)**	Tanzania	2.00 Ma	Age of the Phase III, located 4m under a tuff dated to ~2Ma (Deino 2012 [1], Cueva-Temprana et al. 2022 [2]).	Oldowan	NA
**Olduvai DK (Bed I)**	Tanzania	1.84 Ma	Maximum age (Mora & de la Torre 2005 [3]).	Oldowan	NA
**Olduvai FLK North (Bed I)**	Tanzania	1.76-1.6 Ma	Mora & de la Torre 2005 [3].	Oldowan	NA
**Olduvai HWK-EE (Clay Unit, Bed II)**	Tanzania	1.7-1.15 Ma	Bed II chronology, K-Ar radiometric dating (Hay, 1976 [4]).	Oldowan	**1970** (M. Leakey)
**Olduvai HWK-EE (SC Unit, Bed II)**	Tanzania	1.7-1.15 Ma	Bed II chronology, K-Ar radiometric dating (Hay, 1976 [4]).	Oldowan	**1970** (M. Leakey)
**Olduvai SHK Main Site (Level A & B Bed II)**	Tanzania	1.7-1.15 Ma	Bed II chronology, K-Ar radiometric dating (Hay, 1976 [4]).	Oldowan	1953, 1955, 1957, 2009-2011
**Olduvai BK (Level 1 to 3, Bed II)**	Tanzania	1.48 Ma	Mora & de la Torre 2005 [3]	Oldowan	1952, 1953, 1955, 1957, **2006**
**Olduvai BK (Level 4, Bed II)**	Tanzania	1.338 Ma	BK is located directly above tuff IID dated at 1.338 +/- 0.024 Ma (Domínguez-Rodrigo et al. 2013 [5]).	Oldowan	1952, 1953, 1955, 1957, **2006**
**Olduvai TK (Bed II)**	Tanzania	1.353 Ma	Tuff IID dated by $^{40}Ar/^{39}Ar$ to 1.353 +/- 0.035 Ma (Santonja et al. 2014 [6]).	Acheulian	1963, 2010-2015 (**2010-2012**)
**Melka Kunture (Karre Level K1-2)**	Ethiopia	1.7-1.6 Ma	Karre site can be correlated with the level B of Gombore I, dated by K/Ar (Piperno 2001[7].	Oldowan	**1980**
**Melka Kunture (Garba IV)**	Ethiopia	1.5-1.3 Ma	Absolute age (Piperno 2001 [7]).	Oldowan	1965-1982 & 1993-1995 (J. Chavaillon)
**Melka Kunture (Gombore IB)**	Ethiopia	1.7-1.6 Ma	Piperno 2001[7].	Oldowan	1966, 1967, 1974, 1980 (J. Chavaillon)
**Melka Kunture (Gombore II, Locality 1)**	Ethiopia	0.875 Ma	Gombore II overlies a tuff dated to 0.875 +/- 0.010 Ma (Mussi et al. forthcoming [8]).	Acheulian	1971, 1976
**Melka Kunture (Gombore II, Locality 2)**	Ethiopia	0.709 Ma	Gombore II-2 is covered by a tuff dated to 0.709 +/- 0.0140 Ma (Mussi et al. forthcoming [8]).	Acheulian	Excavations resumed in 1993 and 1995
**Melka Kunture (Gombore II, Locality 3)**	Ethiopia	0.875 Ma	Gombore II overlies a tuff dated to 0.875 +/- 0.010 Ma (Mussi et al. forthcoming [8]).	Acheulian	Gombore II sites: 1966 to 1995
**Melka Kunture (Gombore II, Locality 4)**	Ethiopia	0.875 Ma	Gombore II overlies a tuff dated to 0.875 +/- 0.010 Ma (Mussi et al. forthcoming [8]).	Acheulian	Gombore II sites: 1966 to 1995
**Melka Kunture (Gombore II, Locality 5)**	Ethiopia	0.875 Ma	Gombore II overlies a tuff dated to 0.875 +/- 0.010 Ma (Mussi et al. forthcoming [8]).	Acheulian	Gombore II sites: 1966 to 1995
**Melka Kunture (Gombore Iγ)**	Ethiopia	1.4-1.3 Ma	1.4-1.3 Ma. Final Oldowan/Lower Acheulian (Mussi et al. forthcoming [8]).	Oldowan	1974, 1976, 1978
**Melka Kunture (Simbiro III, level A)**	Ethiopia	0.878 Ma	Minimum age. Tuff overlying the sequence dated to 0.878 +/- 0.014 Ma (Beaudet et al. 2015 [9], Morgan et al. 2012 [10]).	Acheulian	Simbiro III sites: 1974, 1975, 1976, 2005

**Melka Kunture (Simbiro III, level B)**	Ethiopia	0.878 Ma	Minimum age. Tuff overlying the upper level A dated to 0.878 +/- 0.014 Ma. Levels B and C are slightly earlier (Beaudet et al. 2015 [9], Morgan et al. 2012 [10]).	Acheulian	Simbiro III sites: 1974, 1975, 1976, 2005
**Melka Kunture (Simbiro III, level D)**	Ethiopia	0.878 Ma	Minimum age. Tuff overlying the upper level A dated to 0.878 +/- 0.014 Ma (Beaudet et al. 2015 [9], Morgan et al. 2012 [10]).	Acheulian	Simbiro III sites: 1974, 1975, 1976, 2005
**Barogali**	Republic of Djibouti	1.6-1.3 Ma	Chavaillon et al. 1987 [11]	Oldowan	1985, 1986, 1987
**Gadeb 2B**	Ethiopia	1.45-0.7 Ma	Reverse paleomagnetic polarity of the deposits and tephra correlation between Turkana Basin and Gadeb (de la Torre 2011 [12], Haileab & Brown 1994 [13]).	Oldowan	Gadeb: 1975-1978 (D. Clark)
**Gadeb 2C**	Ethiopia	1.45-0.7 Ma	Reverse paleomagnetic polarity of the deposits and tephra correlation between Turkana Basin and Gadeb (de la Torre 2011 [12], Haileab & Brown 1994 [13]).	Oldowan	Gadeb: 1975-1978 (D. Clark)
**Gadeb 2E**	Ethiopia	1.45-0.7 Ma	Reverse paleomagnetic polarity of the deposits and tephra correlation between Turkana Basin and Gadeb (de la Torre 2011 [12], Haileab & Brown 1994 [13]).	Oldowan	Gadeb: 1975-1978 (D. Clark)
**Gadeb 8A**	Ethiopia	1.45-0.7 Ma	Reverse paleomagnetic polarity of the deposits and tephra correlation between Turkana Basin and Gadeb (de la Torre 2011 [12], Haileab & Brown 1994 [13]).	Acheulian	Gadeb: 1975-1978 (D. Clark)
**Gadeb 8F**	Ethiopia	1.45-0.7 Ma	Reverse paleomagnetic polarity of the deposits and tephra correlation between Turkana Basin and Gadeb (de la Torre 2011 [12], Haileab & Brown 1994 [13]).	Oldowan	Gadeb: 1975-1978 (D. Clark)
**Isenya (level V)**	Kenya	0.96 Ma	Minimum age. Stratigraphic correlation with tuffs from Olorgesailie M4 (Durkee & Brown 2014 [14]).	Acheulian	1983-1988
**Isenya (level VIa)**	Kenya	0.96 Ma	Minimum age. Stratigraphic correlation with tuffs from Olorgesailie M4 (Durkee & Brown 2014 [14]).	Acheulian	1983-1988
**Isenya (level VIb21)**	Kenya	0.96 Ma	Minimum age. Stratigraphic correlation with tuffs from Olorgesailie M4 (Durkee & Brown 2014 [14]).	Acheulian	1983-1988

**Olorgesailie Member 2**	Kenya	0.992-0.974 Ma	Between M1 dated at 0.992Ma and M5 at 0.974 Ma, by $^{40}Ar/^{39}Ar$ (Potts et al. 1999 [15]).	Acheulian	Olorgesailie: **1942-1947** (L.S.B. Leakey), 1960s & 1970s (G.L. Isaac), 80s (R. Potts)
**Olorgesailie Member 3**	Kenya	0.992-0.974 Ma	Between Member 1 dated at 0.992 Ma and Member 5 at 0.974 Ma, by $^{40}Ar/^{39}Ar$ (Potts et al. 1999 [15]).	Acheulian	Olorgesailie: **1942-1947** (L.S.B. Leakey), 1960s & 1970s (G.L. Isaac), 80s (R. Potts)
**Olorgesailie Member 6**	Kenya	0.974-0.78 Ma	Between M5 dated at 0.974 Ma by $^{40}Ar/^{39}Ar$ and M8 dated at 0.780 Ma by paleomagnetism (Potts et al. 1999 [15]).	Acheulian	Olorgesailie: **1942-1947** (L.S.B. Leakey), 1960s & 1970s (G.L. Isaac), 80s (R. Potts)
**Olorgesailie Member 7**	Kenya	0.974-0.78 Ma	Between M5 dated at 0.974 Ma by $^{40}Ar/^{39}Ar$ and M8 dated at 0.780 Ma by paleomagnetism (Potts et al. 1999 [15]).	Acheulian	Olorgesailie: **1942-1947** (L.S.B. Leakey), 1960s & 1970s (G.L. Isaac), 80s (R. Potts)
**Olorgesailie Member 8**	Kenya	0.78 Ma	Paleomagnetism (Potts et al. 1999 [15]).	Acheulian	Olorgesailie: **1942-1947** (L.S.B. Leakey), 1960s & 1970s (G.L. Isaac), 80s (R. Potts)
**Olorgesailie Member 9**	Kenya	0.746 Ma	0.746 Ma +/- 9 ka, single-crystal laser-fusion age (Deino & Potts 1992 [16]).	Acheulian	Olorgesailie: **1942-1947** (L.S.B. Leakey), 1960s & 1970s (G.L. Isaac), 80s (R. Potts)
**Olorgesailie Member 10**	Kenya	0.662 Ma	0.662 Ma +/- 4 ka, single-crystal laser-fusion age (Deino & Potts 1992 [16]).	Acheulian	Olorgesailie: **1942-1947** (L.S.B. Leakey), 1960s & 1970s (G.L. Isaac), 80s (R. Potts)
**Olorgesailie Member 11**	Kenya	0.662 Ma	$^{40}Ar/^{39}Ar$ (Potts et al. 1999 [15]).	Acheulian	Olorgesailie: **1942-1947** (L.S.B. Leakey), 1960s & 1970s (G.L. Isaac), 80s (R. Potts)
**Isimila (K6)**	Tanzania	0.26 Ma	Maximum age. U series date a bone from sands 4 to 0.260 Ma +0.070 Ma, -0.040 Ma (Howell et al. 1972 [17]).	Acheulian	Isimila; 1957 (F.C. Howell, G.H. Cole, M.R. Kleindienst), 1958 (M.R. Kleindienst)
**Isimila (LJ6-7)**	Tanzania	0.26 Ma	Maximum age. U series date a bone from sands 4 to 0.260 Ma +0.070 Ma, -0.040 Ma (Howell et al. 1972 [17]).	Acheulian	Isimila; 1957 (F.C. Howell, G.H. Cole, M.R. Kleindienst), 1958 (M.R. Kleindienst)
**Isimila (K14)**	Tanzania	0.26 Ma	Maximum age. U series date a bone from sands 4 to 0.260 Ma +0.070 Ma, -0.040 Ma (Howell et al. 1972 [17]).	Acheulian	Isimila; 1957 (F.C. Howell, G.H. Cole, M.R. Kleindienst), 1958 (M.R. Kleindienst)
**Isimila (H9-38)**	Tanzania	0.26 Ma	Maximum age. U series date a bone from sands 4 to 0.260 Ma +0.070 Ma, -0.040 Ma (Howell et al. 1972 [17]).	Acheulian	Isimila; 1957 (F.C. Howell, G.H. Cole, M.R. Kleindienst), 1958 (M.R. Kleindienst)

**Ounjougou**	Mali	NA	Terminus *ante quem* : 0.15 Ma, OSL. Probably a large hiatus between the most profound deposits (containing PSBs) and Middle Paleolithic remains. The assemblage with PSBs look like an Oldowan facies, in any case to be older than Middle Paleolithic (Soriano et al. 2010 [18]).	Oldowan	1990s
**Kabwe (Broken Hill)**	Zambia	0.299 Ma	0.299 Ma +/- 25 ka. Dating of the skull, U-series (Grün et al. 2020 [19]).	MSA	1921
**Broken Hill (Sangoan)**	Zambia	NA	Sangoan industry (Clark 1959 [20]).	Sangoan	1953
**Broken Hill (Rhodesian Acheulian)**	Zambia	NA	NA	Acheulian	1953
**Broken Hill (Hope Fountain Bariant)**	Zambia	NA	NA	NA	1953
**Kalambo Falls (A4 rubble IIA)**	Zambia	NA	Lupemban industry (Sheppard & Kleindienst 1996 [21]).	Lupemban	1956-1966
**Kalambo Falls (A4 rubble IIB)**	Zambia	NA	Lupemban industry (Sheppard & Kleindienst 1996 [21]).	Lupemban	1956-1966
**Kalambo Falls (A5 Comp)**	Zambia	NA	Acheulian industry (Sheppard & Kleindienst 1996 [21]).	Acheulian	1956-1966
**Cave of Hearths (Bed 1-3)**	South Africa	0.77-0.5 Ma	Paleomagnetism attests that the site is not older than 0.78 Ma. Secure age of 0.5 Ma to 0.77 Ma (synthesis in Lambert-Law de Lauriston 2005 [22]). Late Acheulian.	Acheulian	1937 (discovery of the site, C. van Riet Lowe), 1938 (1rst discovery of lithics), 1947 (1rst phase of systematic investigations, G. Gardner), 1953-1954 (R. Mason)
**Swartkrans (SWT-M1, SPRP excavation)**	South Africa	2.19-1.8 Ma	Gibbon et al. 2014 [23]: lower bank of M1 dated by cosmogenic nucleid (Al26 and Be10) to 2.19 Ma +/- 0.0 8 Ma (dating of a manuport) and 1.80 Ma +/- 0.09 Ma (dating of sediments).	Oldowan	Swartkrans: 1960s (C.K. Brain), 1965-1986 (C.K. Brain), **2005-present** (SPRP, Swartkrans Paleoanthropology Research Project).
**Swartkrans (SWT-M1, LB)**	South Africa	2.19-1.8 Ma	Gibbon et al. 2014 [23]: lower bank of M1 dated by cosmogenic nucleid (Al26 and Be10) to 2.19 Ma +/- 0.08 Ma (dating of a manuport) and 1.80 Ma +/- 0.09 Ma (dating of sediments).	Oldowan	Swartkrans: 1960s (C.K. Brain), 1965-1986 (CK. Brain), 2005-present (SPRP, Swartkrans Paleoanthropology Research Project).
**Swartkrans (SWT-M2)**	South Africa	1.5-1.0 Ma	Clark 1993 [24]	Oldowan	Swartkrans: 1960s (C.K. Brain), 1965-1986 (C.K. Brain), 2005-present (SPRP, Swartkrans Paleoanthropology Research Project).

**Swartkrans (SWT-M3)**	South Africa	1.0 Ma	0.96 Ma +/- 9 ka (Gibbon et al. 2014 [23]). Clark 1993 [24]	Acheulian	Swartkrans: 1960s (C.K. Brain), 1965-1986 (C.K. Brain), 2005-present (SPRP, Swartkrans Paleoanthropology Research Project).
**Vlakkraal Thermal springs**	South Africa	NA	MSA industry (Wells et al. 1942 [25]).	MSA	1931-1932 (Dreyer), 1939 (J. F. Hartman), 1949 (L.H. Wells & H.B.S. Cooke)
**Windhoek**	Namibia	NA	MSA lithics and "found at considerable depths in springs" (Walker 2008 [26]).	MSA	NA
**Esere**	Namibia	NA	"found at considerable depths in springs" (Walker 2008 [26]).	MSA	NA
**Rhino Cave (Tsodilo Hills)**	Botswana	NA	NA	MSA	1995 & 1996 (Robbins), 2004-2006 (test-excavations)
**Corner Cave (Tsodilo Hills)**	Botswana	NA	NA	MSA	NA
**Kalkbank**	South Africa	NA	MSA site (Mason et al. 1958 [27]).	MSA	**1954** (R. J. Mason)
**Florisbad**	South Africa	0.279-0.121 Ma	All the MSA sequence, dated by OSL and ESR (Grün et al. 1996 [28], Kuman et al. 1999 [29]).	MSA	**1932** (T.F. Dreyer), **1952** (A.C. Hoffman, T.F. Dreyer), 1980s
**Sterkfontein (Member 5)**	South Africa	2.18 Ma	Oldowan or Acheulian. Maximum age of the sequence; earliest stone tools dated to 2.18 Ma +/- 0.21 Ma (Granger et al. 2015 [30]).	NA	1930s, 1997
**El Guettar**	Tunisia	NA	"Mousterian" lithic assemblage (Gruet 1950 [31]).	Mousterian	1939 (prospection, M. Gruet), 1949 (survey, . Gruet)
**Ain El Hallouf**	Morroco	NA	NA	NA	NA
**Sidi Abderrahmane**	Morroco	NA	NA	Acheulian	1950s
**Sidi Abderrahmane STIC**	Morroco	NA	MIS16 minimum age, middle Acheulian (Raynal et al. 2005 [32]).	Acheulian	1950s
**Erg Tihodaine (Coll. Arambourg)**	Algeria	NA	Acheulian assemblage (industry similar to the one of an Acheulian IS13 level), Middle Pleistocene age (Hocine 2016 [33]).	Acheulian	End of 19e century (description of the archaeological material), **1948** (C. Arambourg)
**Tighennif I**	Algeria	0.7 Ma	Paleomagnetism, biochronology (Geraads et al. 1985 [34]).	Acheulian	1870s, 1880s, 1925 or 1928, 1931 (C. Arambourg), 1954-1956 (C. Arambourg & R. Hoffstetter), 1981-1983 (J.J. Jaeger)

**Ain Hanech**	Algeria	1.78 Ma	Paleomagnetism (polarity correlated with the Olduvai N subchron dated between 1.78-1.95 Ma), biochronology (synthesis in Sahnouni & Heinzelin 1998 [35], Sahnouni 2006 [36]).	Oldowan	1940s, 1952 (excursion), 1992, 1993
**Nzako Ambilo**	Central African Republic	NA	Lupemban industry (Mesfin 2018 [37]).	Lupemban	1967 & 1968 (surveys, R. Bayle des Hermens)
**Nzako Kono**	Central African Republic	NA	Lupemban industry (Mesfin 2018 [37]).	Lupemban	1967 & 1968 (surveys, R. Bayle des Hermens)
**M'Piaka**	Republic of Congo	NA	Lupemban industry (Mesfin 2018 [37]).	Lupemban	1930s (first collects: G. Droux, G. Bergeaud, V. Babet), 1997
**Hummal (Levels 17 & 18)**	Syria	NA	No absolute dating. Located before the Acheulian sequence of El Meirah dated to the palaeomagnetic reversion Matuyama-Brunhes (Le Tensorer et al. 2011 [38]).	Core-and-Flake	1999 (J.M. Le Tensorer & Sultan Muhesen). 2003-?
**Shuwayhitiyah**	Saudia Arabia	NA	NA	Core-and-Flake	1977 (initial survey)
**'Ubeidiya (III-20)**	Israel	1.5-1.2 Ma	'Ubeidiya; biochronological correlations and paleomagnetism (Martínez-Navarro et al. 2009 [39]).	Acheulian	'Ubeidiya: 1960-1966 (M. Stekelis), 1967-1974 (O. Bar-Yosef & E. Tchernov), **1988-1994** (O. Bar-Yosef & J.J. Shea, E. Tchernov, C. Guérin), 1997-1999 (O. Bar-Yosef, J. J. Shea, G. Bozinski, E. Tchernov)
**'Ubeidiya (III-22)**	Israel	1.5-1.2 Ma	'Ubeidiya; biochronological correlations and paleomagnetism (Martínez-Navarro et al. 2009 [39]).	Acheulian	'Ubeidiya: 1960-1966 (M. Stekelis), 1967-1974 (O. Bar-Yosef & E. Tchernov), **1988-1994** (O. Bar-Yosef & J.J. Shea, E. Tchernov, C. Guérin), 1997-1999 (O. Bar-Yosef, J.J. Shea, G. Bozinski, E. Tchernov)
**Dursunlu**	Turkey	0.99-0.78 Ma	Paleomagnetism: the layer "predates the Brunhes/Matuyama boundary but post-dates the Jaramillo" (Güleç et al. 2009 [40]).	Acheulian	1993-1996 (collect of fossil vertebrate, E. Guleç, F. Clark Howell, T.D. White)
**North of Bridge Acheulian (NBA)**	Israel	0.658 Ma	Ar/Ar dating of a basalt flow just below the NBA horizon: 0.658 Ma +/- 15 ka (Sharon et al. 2010 [41]).	Acheulian	1999
**Latamné**	Syria	0.560 Ma	TL age. Formation is 0.56 Ma, and the entire sequence is dated between 0.7 Ma-0.5 Ma (Bar-Yosef 1994 [42]).	Acheulian	**1962 & 1964** (Clark), 1967-1969 (Clark), 1993 (Sanlaville)

**Joubb Jannine II**	Lebanon	0.560 Ma	Formation correlated to the one of Latamné (Yazbeck 2002 [43]).	Acheulian	1966, 1968 (surveys, J. Besançon & F. Hours, with selection of "belles pièces"), 1970s, 1995 (J. Tixier)
**Khalliyé Sud**	Lebanon	NA	NA	Acheulian	Discovered in 1973
**Wadi Fatimah**	Saudia Arabia	NA	NA	Acheulian	NA
**Revadim Quarry (Area D)**	Israel	0.78-0.3 Ma	<0.78 Ma according to paleomagnetism. At least 0.5 Ma-0.3 Ma according to U-series performed on carbonate coating on flint objects (Marder et al. 1999 [44], Solodenko et al. 2015 [45]).	Acheulian	Four seasons of excavations since 1996
**Evron Quarry**	Israel	0.69-0.33 Ma	Optically and infrared stimulated luminescence, TL, ESR (Porat & Ronen 2002 [46]).	Acheulian	1976-1977, 1985
**Evron East**	Israel	NA	NA	Acheulian	2014
**Saffaqah**	Saudia Arabia	0.188 Ma	Layer D dated to 188ka +/- 11 ka, providing a *terminus post quem* for the overlying Layers B and C. Luminescence dating (Scerri et al. 2018 [47]).	Acheulian	Systematic survey and 2 seasons of excavations
**Qesem Cave**	Israel	0.42-0.2 Ma	TL, ESR, U-series dates (Barkai et al. 2003 [48], Gopher et al. 2010 [49], Mercier et al. 2013 [50], Falguères et al. 2016 [51]).	Acheulian	2000s
**Bezez**	Lebanon	NA	Acheuleo-Yabrudian (late Lower Paleolithic) (Barkai & Gopher 2016 [52]).	Acheulian / Yabrudian	1958-1963
**Ma'ayan Barukh**	Israel	0.35 Ma	Minimum age. U-series on travertine enclosing the site (Feraud et al. 1983 [53]).	Acheulian	1920s (occasional collect of flint tools)
**Kaletepe Deresi 3 (Level III)**	Turkey	1.0 Ma	Maximum age of Kaletepe Deresi 3 sequence. Tephrochronology, K/Ar (Slimak et al. 2008 [54]). Late Acheulian (Slimak et al. 2007 [55]).	Acheulian	2000-2006, 2008
**Kaletepe Deresi 3 (Level III/IV)**	Turkey	1.0 Ma	Maximum age of Kaletepe Deresi 3 sequence. Tephrochronology, K/Ar (Slimak et al. 2008 [54]). Late Acheulian (Slimak et al. 2007 [55]).	Acheulian	2000-2006, 2008
**Kaletepe Deresi 3 (Level IV)**	Turkey	1.0 Ma	Maximum age of Kaletepe Deresi 3 sequence. Tephrochronology, K/Ar (Slimak et al. 2008 [54]). Late Acheulian (Slimak et al. 2007 [55]).	Acheulian	2000-2006, 2008

**Kaletepe Deresi 3 (Level V)**	Turkey	1.0 Ma	Maximum age of Kaletepe Deresi 3 sequence. Tephrochronology, K/Ar (Slimak et al. 2008 [54]). Late Acheulian (Slimak et al. 2007 [55]).	Acheulian	2000-2006, 2008
**Kaletepe Deresi 3 (Level Vam)**	Turkey	1.0 Ma	Maximum age of Kaletepe Deresi 3 sequence. Tephrochronology, K/Ar (Slimak et al. 2008 [54]). Late Acheulian (Slimak et al. 2007 [55]).	Acheulian	2000-2006, 2008
**Kaletepe Deresi 3 (Level V')**	Turkey	1.0 Ma	Maximum age of Kaletepe Deresi 3 sequence. Tephrochronology, K/Ar (Slimak et al. 2008 [54]). Late Acheulian (Slimak et al. 2007 [55]).	Acheulian	2000-2006, 2008
**Kaletepe Deresi 3 (Level VI')**	Turkey	1.0 Ma	Maximum age of Kaletepe Deresi 3 sequence. Tephrochronology, K/Ar (Slimak et al. 2008 [54]). Late Acheulian (Slimak et al. 2007 [55]).	Acheulian	2000-2006, 2008
**Kaletepe Deresi 3 (Level VII)**	Turkey	1.0 Ma	Maximum age of Kaletepe Deresi 3 sequence. Tephrochronology, K/Ar (Slimak et al. 2008 [54]). Late Acheulian (Slimak et al. 2007 [55]).	Acheulian	2000-2006, 2008
**Kaletepe Deresi 3 (Level VIII)**	Turkey	1.0 Ma	Maximum age of Kaletepe Deresi 3 sequence. Tephrochronology, K/Ar (Slimak et al. 2008 [54]). Late Acheulian (Slimak et al. 2007 [55]).	Acheulian	2000-2006, 2008
**Kaletepe Deresi 3 (Level IX)**	Turkey	1.0 Ma	Maximum age of Kaletepe Deresi 3 sequence. Tephrochronology, K/Ar (Slimak et al. 2008 [54]). Late Acheulian (Slimak et al. 2007 [55]).	Acheulian	2000-2006, 2008
**Kaletepe Deresi 3 (Level X)**	Turkey	1.0 Ma	Maximum age of Kaletepe Deresi 3 sequence. Tephrochronology, K/Ar (Slimak et al. 2008 [54]). Late Acheulian (Slimak et al. 2007 [55]).	Acheulian	2000-2006, 2008
**Kaletepe Deresi 3 (Level XI)**	Turkey	1.0-0.16 Ma	Maximum age of Kaletepe Deresi 3 sequence. Tephrochronology, K/Ar (Slimak et al. 2008 [54]). Late Acheulian (Slimak et al. 2007 [55]).	Acheulian	2000-2006, 2008
**Kaletepe Deresi 3 (Level XII)**	Turkey	1.0-0.16 Ma	Maximum age of Kaletepe Deresi 3 sequence. Tephrochronology, K/Ar (Slimak et al. 2008 [54]). Late Acheulian (Slimak et al. 2007 [55]).	Acheulian	2000-2006, 2008
**Santa Ana Cave**	Spain	NA	NA	Acheulian	2000-2001 (E. Carbonell, A. Canals, I. Sauceda)

**Barranco León**	Spain	1.4-1.2 Ma	Relative and absolute dating: biochronology, magnetostratigraphy, U-series, ESR (synthesis in Barsky et al. 2015 [56]).	Cores-and-Flakes	1995, 1999-2000 (G. Martinez-Fernandez & I. Toro-Moyano), 2001- 2005 (I. Toro-Moyano), 2010 (General Project, coordinated by R. Sala Ramos & B. Martinez Navarro), 2012-2014 (General Project), 2016-present (J. Garcia Solano)
**Bois-de-Riquet (Unit 4)**	France	0.78-0.68 Ma	Cosmogenic dating of the mudflow event (beginning of the Middle Pleistocene). Dated between 0.78 Ma BP and 0.68 Ma BP (age of St. Thibéry volcano) (synthesis in Bourguignon et al. 2016 [57]).	Acheulian	Bois de Riquet site: 2008, 2000s, 2010s, 2020s
**Ca' Belvedere di Monte Poggiolo**	Italy	0.99-0.78 Ma	Reverse magnetic phase slightly before Jaramillo, and radiometric dating (Terradillos Bernal & Moncel 2004 [58]).	Core-and-Flake	Discovered in 1984
**Dorn-Dürkheim 3**	Germany	0.82-0.78 Ma	Biostratigraphy, late Matuyama chron, MIS 21-19. Beginning of Lower Palaeolithic in central Europe (Fiedler et al. 2019 [59]).	NA	1989-1996 (J.-L. Franzen)
**La Noira (Stratum c)**	France	0.449 Ma	ESR, top of stratum c, 449 ka +/-45 ka. Lithics abandoned during a temperate phase (Iovita et al. 2017 [60]).	Acheulian	2010-2018 (stratum a), 20 years of filed surveys (stratum c)
**Caune de l'Arago (Units H1,2,3)**	France	0.5 Ma	Barsky 2001 [61]	Acheulian	Caune de l'Arago: 1838 (first description of fauna, M. de Serres), 1948 (first recognition of prehistoric lithic artefacts, J. Ablanet), 1956 & 1960 (collect of artefacts, i.e. R. Ribes), 1964-present (systematic excavation, H. de Lumley)
**Caune de l'Arago (Unit G)**	France	0.45 Ma	Barsky 2001 [61]	Acheulian	Caune de l'Arago: 1838 (first description of fauna, M. de Serres), 1948 (first recognition of prehistoric lithic artefacts, J. Ablanet), 1956 & 1960 (collect of artefacts, i.e. R. Ribes), 1964-present (systematic excavation, H. de Lumley)

**Caune de l'Arago (Unit E)**	France	0.42 Ma	Barsky 2001 [61]	Acheulian	Caune de l'Arago: 1838 (first description of fauna, M. de Serres), 1948 (first recognition of prehistoric lithic artefacts, J. Ablanet), 1956 & 1960 (collect of artefacts, i.e. R. Ribes), 1964-present (systematic excavation, H. de Lumley)
**Caune de l'Arago (Unit D)**	France	0.41 Ma	Barsky 2001 [61]	Acheulian	Caune de l'Arago: 1838 (first description of fauna, M. de Serres), 1948 (first recognition of prehistoric lithic artefacts, J. Ablanet), 1956 & 1960 (collect of artefacts, i.e. R. Ribes), 1964-present (systematic excavation, H. de Lumley)
**Treugol'Naya Cave (assemblage II)**	Russia	0.427-0.364 Ma	Core Chopper industry (Doronichev & Golovanova 2010 [62]).	Core-Chopper	1986-1988
**Duclos (0)**	France	NA	Acheulian, "faciès pyrénéogaronnais" (Colonge 2012 [63]).	Acheulian	2008
**Duclos (Ensemble IV)**	France	0.235-0.169 Ma	OSL, TL: top of level 1 dated to 0.169 Ma +/- 0.019 Ma; base of level 2 dated to 0.235 Ma +/- 0.017 Ma. Acheulian, "faciès pyrénéogaronnais" (Colonge 2012 [63]).	Acheulian	2008
**Duclos (Ensemble III)**	France	0.235-0.169 Ma	OSL, TL: top of level 1 dated to 0.169 Ma +/- 0.019 Ma; base of level 2 dated to 0.235 Ma +/- 0.017 Ma. Acheulian, "faciès pyrénéogaronnais" (Colonge 2012 [63]).	Acheulian	2008
**Septsos**	France	NA	Chronologically quite similar to Duclos (Colonge 2012 [63]).	Acheulian	2008
**Cerveny Kopec**	Czech Republic	NA	NA	NA	1962 (B. Klima, found 2 flakes in quartzite), 1972 (K. Gebauer, found an object in quartz), 1991 (J. Svoboda, found another object in quartz)
**Bañugues (Asturias del Esferoid)**	Spain	NA	NA	Acheulian	End of 1977-1981 (A. Rodríguez Asencio)
**Tourville-la-Rivière (level D2)**	France	NA	ESR and ESR/U-series dated the site to the MIS7 (Bahain et al. 2020 [64]).	Middle Palaeolithic	End of 1960s (G. Carpentier), 1981 (G. Carpentier), 1982, **1983**, 1984 (L. Vallin), **2008** (D. Cliquet)

**Chez-Pinaud Jonzac (US 22)**	France	NA	Quina Mousterian level, dated by TL to the MIS4 (Richter et al. 2013 [65]).	Mousterian	1998-2003 (J. Airvaux), 2004-2008 (J. Jaubert & J.J. Hublin)
**La Quina (level 8)**	France	0.048-0.04 Ma	Denticulate Mousterian. Layers 8 to 6a in a hiatus dated by TL between 48 and 40 ka. MIS 3 (Debénath et al. 1998 [66]).	Mousterian	1872 (G. Chauvet), 1881 (collect), 1886 (Ramonet), 1906-1936 (H. Henri-Martin), 1952-1972 (G. Henri-Martin), **1985-1994** (A. Débénath)
**Festons (Rebières valley)**	France	NA	Mousterian or Aurignacian (Pittard & Donici 1927 [67]).	NA	Discovered in 1908, dug during the first half of the 20e century.
**Sablière Rambour (Villers- Bocage)**	France	NA	NA	NA	NA
**Isle-Adam (sablière de Cassan)**	France	NA	NA	Middle Palaeolithic	1942
**Coll de la Guille (Terrasses du Roussillon)**	France	NA	T3C Terrasse. See Collina-Girard 1986 [68].	NA	NA
**Mas Ferreol (Terrasses du Roussillon)**	France	1.1 Ma	Terrasse T5. Synthesis in Garcia Garriga 2014 [69]: attributed to the Günz (1.1 Ma), dated to 1.07-0.99 Ma BP, or attributed to the Villafranchian (2.5-1 Ma BP).	Core-and-Flake	NA
**Mas Ferrer (Terrasses du Roussillon)**	France	1.1 Ma	Terrasse T5/4. Synthesis in Collina-Girard 1976 [70]: as Cabsetany, attributed to the Günz (1.1 Ma BP), to the Villafranchian (2.5-1 Ma BP).	Core-and-Flake	NA
**Le Puech de la Boule (Terrasses du Roussillon)**	France	NA	Terrasse T4. See Collina-Girard 1986 [68].	NA	NA
**Mas Bruno (Terrasses du Roussillon)**	France	NA	NA	NA	NA
**Cabestany général (Terrasses du Roussillon)**	France	1.1 Ma	Terrasse T5. Synthesis in Collina-Girard 1976 [70]: attributed to the Günz (1.1 Ma BP,), to the Villafranchian (2.5-1 Ma BP).	Core-and-Flake	NA
**La Llabanère (Terrasses du Roussillon)**	France	0.374-0.44 Ma	Terrasse T3B. ESR, 2 samples : 374 ka+/-47 ka (MIS 10), 440 ka+/-39 ka (Delmas et al. 2018 [71]).	Acheulian	NA
**Singi Talav (Layer 3)**	India	0.8 Ma	Minimum age. Correlation with the middle part of the Amarpura quarry (Gaillard & Rajaguru 2017 [72]).	Acheulian	1981-1985 (V.N. Misra & S.N. Rajaguru)
**Singi Talav (Layer 4)**	India	0.8 Ma	Minimum age. Correlation with the middle part of the Amarpura quarry (Gaillard & Rajaguru 2017 [72]).	Acheulian	1981-1985 (V.N. Misra & S.N. Rajaguru)
**Torajunga**	India	NA	NA	Middle Palaeolithic	NA
**Chirki Nevasa**	India	NA	NA	Acheulian	**1966-1969**

**Atit 2**	India	NA	NA	Acheulian	2015-2016 (J. Joglekar
**Zhoukoudian 1 (Layer 1-3)**	China	0.4 Ma	Layers 1 to 14 may be dated to MIS 11 to MIS 19. Cosmogenic dating of sediment (26Al/10Be), U-series, dating of *Homo erectus* remains Shen et al. 2009 [73]).	NA	Zhoukoudian: 1921-1923 (O. Zdansky), 1927 (L. Jie), end of 1920s- 1934 (D. Black), 1934-1937 (F. Weidenreich), 1949-1966, 1978-982, 1998
**Zhoukoudian 1 (Layer 4-5)**	China	0.5-0.6 Ma	Layers 1 to 14 may be dated to MIS 11 to MIS 19. Cosmogenic dating of sediment (26Al/10Be), U-series, dating of *Homo erectus* remains Shen et al. 2009 [73]).	NA	Zhoukoudian: 1921-1923 (O. Zdansky), 1927 (L. Jie), end of 1920s- 1934 (D. Black), 1934-1937 (F. Weidenreich), 1949-1966, 1978-982, 1998
**Zhoukoudian 1 (QII)**	China	0.75-0.4 Ma	Age of the sequence. Layers 1 to 14 may be dated to MIS 11 to MIS 19. Cosmogenic dating of sediment (26Al/10Be), U-series, dating of *Homo erectus* remains Shen et al. 2009 [73]).	NA	Zhoukoudian: 1921-1923 (O. Zdansky), 1927 (L. Jie), end of 1920s- 1934 (D. Black), 1934-1937 (F. Weidenreich), 1949-1966, 1978-982, 1998
**Zhoukoudian 1 (Layer 8-9)**	China	0.67-0.75 Ma	Layers 1 to 14 may be dated to MIS 11 to MIS 19. Cosmogenic dating of sediment (26Al/10Be), U-series, dating of *Homo erectus* remains Shen et al. 2009 [73]).	NA	Zhoukoudian: 1921-1923 (O. Zdansky), 1927 (L. Jie), end of 1920s- 1934 (D. Black), 1934-1937 (F. Weidenreich), 1949-1966, 1978-982, 1998
**Liangshan Longgangsi**	China	0.6 Ma	TT-OSL (Li et al. 2014 [74]).	Acheulian	1951 (first discoveries of artefacts), 1980 (Y. Jiaqi), 1982-1985 (H. Weiwen), **1985** (surfacic collect), 1986
**Dingcun**	China	0.21-0.16 Ma	U-series on mammal teeth (Chen et al. 1984 [75]).	Acheulian	**1953-1954**
**Gongwangling**	China	NA	Paleomagnetism: 0.80 Ma-0.75 Ma BP (Ma X.S. et al. 1978 [76]; Cheng et al. 1978) [77]. An Z. & Ho C. (1989) [78] date it to 1.16 Ma. All these dates may be for the hominin skull, not lithics.	NA	1963-1966
**Ganyu**	China	NA	NA	Acheulian	NA
**Maling 2A**	China	0.386-0.221 Ma	Pei et al. 2015 [79]	Acheulian	**2011**
**Shuigou-Huixinggou**	China	0.9 Ma	Reversed polarity below the Matuyama-Brunhes boundary, MIS 23 (Li et al. 2017 [80]).	Acheulian	NA
**Zhoukoudian 15**	China	0.14-0.11 Ma	U-series (Gao 2000 [81]).	Middle Palaeolithic	**1935-1937**
**Xujiayao**	China	0.125-0.104 Ma	U-series (Yang et al. 2019 [82]).	Middle Palaeolithic	1976, 1977, 1979

**Lingjing (Layer 11, lower part of layer 10)**	China	0.125-0.09 Ma	OSL (Li et al. 2019 [83]).	Middle Palaeolithic	**2005-2006**
**Hsuchiyao**	China	0.1 Ma	NA	Middle Palaeolithic	1973-1974 (Chia & Chi), 1976-1977 (Chia & Chi)
**Diaozhai**	China	0.07-0.03 Ma	OSL (Wang et al. 2014 [84]).	Acheulian	2010s
**Jijiawan**	China	0.428-0.071 Ma	Dating of the artefacts layers (Zhuo et al. 2016 [85]).	NA	NA
**Houjiapu**	China	NA	NA	NA	NA
**Zhoupo (Locality 95LP07)**	China	0.5-0.1828 Ma	TL. 182,8 ka is the minimum age of Zhoupo sequence. Layer 12 of Zhoupo: 251,05 ka +/- 12,5 ka. Layer 15: 182,8+/-9,1 ka. If compared to Chenjiawo (Lantian), earliest layers of Zhoupo could be dated to 500 ka Wang 2005 [86].	NA	NA
**Mansuri (Locality 1)**	Korea	NA	NA	NA	NA
**Jeongok-Ri (surface)**	Korea	0.195 Ma	195 ka +/-12 ka, TT-OSL (de Lumley et al. 2011 [87])	NA	1980-1987, 1994-1995, 2000-2001 (Kidong Bae, Youg-Hoon Hwang, Youg-Wha Chong, Mou-Chang Choi), 2009 (Pr. Seonbok Yi).
**Jeongok-Ri (Layer 1)**	Korea	0.195 Ma	195 ka +/-12 ka, TT-OSL (de Lumley et al. 2011 [87])	NA	1980-1987, 1994-1995, 2000-2001 (Kidong Bae, Youg-Hoon Hwang, Youg-Wha Chong, Mou-Chang Choi), 2009 (Pr. Seonbok Yi).
**Jeongok-Ri (Layer 2)**	Korea	0.195 Ma	195 ka +/-12ka, TT-OSL (de Lumley et al. 2011 [87])	NA	1980-1987, 1994-1995, 2000-2001 (Kidong Bae, Youg-Hoon Hwang, Youg-Wha Chong, Mou-Chang Choi), 2009 (Pr. Seonbok Yi).
**Jeongok-Ri (Layer 3)**	Korea	0.195 Ma	195 ka +/-12 ka, TT-OSL (de Lumley et al. 2011 [87])	NA	1980-1987, 1994-1995, 2000-2001 (Kidong Bae, Youg-Hoon Hwang, Youg-Wha Chong, Mou-Chang Choi), 2009 (Pr. Seonbok Yi).
**Jangnamgyo (surface)**	Korea	0.5-0.13 Ma	Ar/Ar dating method (de Lumley et al. 2011 [87]).	NA	2009 (Kidong Bae)
**Jangnamgyo (Level 3)**	Korea	0.5-0.13 Ma	Ar/Ar dating method (de Lumley et al. 2011 [87]).	NA	2009 (Kidong Bae)
**Ngebung**	Indonesia	0.8 Ma	Ar/Ar, ESR, U/Th (Fauzi et al. 2016 [88]).	Acheulian	**1990s**
**Banjarejo**	Indonesia	NA	NA	NA	NA
**Matar**	Indonesia	0.165 Ma	U-series (Bartstra et al. 1988 [89]). According to Fauzi et al. 2016 [88] the site is not younger than 120-60 ka.	NA	1931-1933, 1986 (visit of G.J. Bartstra to collect samples for U-series), 2012-2014.
**Solo**	Indonesia	NA	NA	NA	NA
**Baksoko River**	Indonesia	NA	NA	NA	NA

References

1. Deino AL. $^{40}Ar/^{39}Ar$ dating of Bed I, Olduvai Gorge, Tanzania, and the chronology of early Pleistocene climate change. J Hum Evol. 2012; 63: 251-73.
2. Cueva-Temprana A, Lombao D, Soto M, Itambu M, Bushozi P, Boivin N, Petraglia M, Mercader J. Oldowan technology amid shifting environments ~2.03-1.83 million years ago. Front Ecol Evol. 2022; 10: 788101.
3. Mora R, de la Torre I. Percussion tools in Olduvai Beds I and II (Tanzania): implication for early human activities. J Anthropol Archaeol. 2005; 24: 179-92.
4. Hay RL. Geology of the Olduvai Gorge: a study of sedimentation in a semiarid basin. Los Angeles: University of California Press; 1976. 203 p.
5. Domínguez-Rodrigo M, Pickering TR, Baquedano E, Mabulla A, Mark DF, Musiba C et al. First partial skeleton of a 1.34-million-year-old *Paranthropus boisei* from Bed II, Olduvai Gorge, Tanzania. PloS One. 2013 Dec 5; 8(12): e80347.
6. Santonja M, Panera J, Rubio-Jara S, Pérez-González A, Uribelarrea D, Domínguez-Rodrigo M et al. Technological strategies and the economy of raw materials in the TK (Thiongo Korongo) lower occupation, Bed II, Olduvai Gorge, Tanzania. Quat Int. 2014; 322-323: 181-208.
7. Piperno M. The prehistory of Melka Kunture (Ethiopia). Bulletin du Centre de recherche français à Jérusalem 2001 Mar; 8: 135-45.
8. Mussi M, Altamura F, Di Bianco L, Bonnefille R, Gaudzinski-Windheuser S, Geraads D et al. After the emergence of the Acheulean at Melka Kunture (Upper Awash, Ethiopia): From Gombore IB (1.6 Ma) to Gombore Iγ (1.4 Ma), Gombore 1σ (1.3 Ma) and Gombore II OAM Test Pit C (1.2 Ma). Quat Int. Forthcoming.
9. Beaudet A, Zanolli C, Engda Redae B, Endalamaw M, Braga J, Macchiarelli R. A new cercopithecoid dentognathic specimen attributed to *Theropithecus* from the late Early Pleistocene (c. 1 Ma) deposits of Simbiro, at Melka Kunture, Ethiopian highlands. C R Palevol. 2015; 14: 657-69.
10. Morgan LE, Renne PR, Kieffer G, Piperno M, Gallotti R, Raynal JP. A chronological framework for a long and persistent archaeological record: Melka Kunture Ethiopia. J Hum Evol. 2012; 62: 104-15.
11. Chavaillon J, Boisaubert JL, Faure M, Guérin C, Ma JL, Nickel B et al. Le site de dépeçage pléistocène à *Elephas recki* de Barogali (République de Djibouti) : nouveaux résultats et datation. C R Acad Sci II. 1987; 305(15): 1259-66.
12. De la Torre. The Early Stone Age lithic assemblages of Gadeb (Ethiopia) and the Developed Oldowan/early Acheulean in East Africa. J Hum Evol. 2011; 60: 768-812.
13. Haileab B, Brown FH. Tephra correlations between the Gadeb prehistoric site and the Turkana Basin. J Hum Evol. 1994; 26(2): 167-73.
14. Durkee H, Brown FH. Correlation of volcanic ash layers between the Early Pleistocene Acheulean sites of Isnya, Kariandusi, and Olorgesailie, Kenya. J Archaeol Sci. 2014; 49: 510-17.
15. Potts R, Behrensmeyer AK, Ditchfield P. Paleolandscape variation and Early Pleistocene hominid activities: Members 1 and 7, Olorgesailie Formation, Kenya. J Hum Evol. 1999; 37: 747-88.
16. Deino A, Potts R. Age-probability spectra for examination of single-crystal $^{40}Ar/^{39}Ar$ dating results: examples from Olorgesailie, Southern Kenya rift. Quat Int. 1992; 13-14: 47-53.
17. Howell FC, Cole GH, Kleindienst MR, Szabo BJ, Oakley KP. Uranium-series dating of bone from the Isimila prehistoric site, Tanzania. Nature 1972; 237: 51-2.
18. Soriano S, Rasse M, Tribolo C, Huysecom E. Ounjougou: a long Middle Stone Age sequence in the Dogon country (Mali).

In: Allsworth-Jones P, editor. West African archaeology. New developments, new perspectives. BAR International Series Vol. S2164. Oxford: Archaeopress. p. 1-14.

19. Grün R, Pike A, McDermott F, Eggins S, Mortimer G, Aubert M et al. Dating the skull from Broken Hill, Zambia, and its position in human evolution. Nature 2020; 580: 372-88.
20. Clark JD. Further excavations at Broken Hill, Northern Rhodesia. J R Anthropol Inst. 1959; 89(2): 201-32.
21. Sheppard PJ, Kleindienst MR. Technological change in the Earlier and Middle Stone Age of Kalambo Falls (Zambia). Afr Archaeol Rev. 1996; 13(3): 171-96.
22. Lambert-Law de Lauriston TS. An exploration of use-wear analysis on Acheulean large cutting tools: the Cave of Hearths' Bed 3 assemblage [master thesis]. Johannesburg, South Africa: University of the Witwatersrand; 2015.
23. Gibbon RJ, Pickering TR, Sutton MB, Heaton JL, Kuman K, Clarke RJ et al. Cosmogenic nuclide burial dating of hominin-bearing Pleistocene cave deposits at Swartkrans, South Africa. Quat Geochronol. 2014; 24: 10-15.
24. Clark JD. Stone artefact assemblages from Members 1-3, Swartkrans Cave. In: Brain C, editor. Swartkrans: a cave's chronicle of early man. Transvaal Museum Monograph No. 8. Pretoria: Transvaal Museum; 1993. 167-94.
25. Wells LH, Cooke HBS, Malan BD, Wells LH, Cooke HBS. The associated fauna and culture of the Vlakkraal Thermal Springs, O.F.S. Transactions of the Royal Society of South Africa 1942; 29(3): 203-33.
26. Walker N. Through the crystal ball: making sense of spheroids in the Middle Stone Age. The South African Archaeological Bulletin 2008 Jun; 63(187): 12-17.
27. Mason RJ, Dart RA, Kitching JW. Bone tools at the Kalkbank Middle Stone Age site and the Makapansgat Australopithecine locality, central Transvaal. The South African Archaeological Bulletin 1958 Sep; 13(51); 85-116.
28. Grün R, Brink JS, Spooner NA, Taylor L, Stringer CB, Franciscus RG et al. Direct dating of Florisbad hominid. Nature 1996; 382: 500-1.
29. Kuman K, Inbar M, Clarke RJ. Paleoenvironments and cultural sequence of the Florisbad Middle Stone Age hominid site, South Africa. J Archaeol Sci. 1999; 26: 1409-25.
30. Granger DE, Gibbon RJ, Kuman K, Clarke RJ, Bruxelles L, Caffee MW. New cosmogenic burial ages for Sterkfontein Member 2 *Australopithecus* and Member 5 Oldowan. Nature 2015 Jun; 522: 85-92.
31. Gruet M. Note préliminaire sur le gisement moustérien d'El Guettar. Bulletin de la Société préhistorique de France 1950; 47(5): 232-41.
32. Raynal JP, Sbihi Alaoui FZ, Magoga L, Mohib A, Zouak M. The Lower Palaeolithic sequence of Atlantic Morocco revisited after recent excavations at Casablanca. Bulletin d'Archéologie Marocain, Institut National des Sciences de l'Archéologie et du Patrimoine 2004; 20: 44-76.
33. Hocine S. Le site acheuléen d'Erg Tihodaïne : caractéristiques technologiques de l'industrie lithique du Pléistocène moyen (Sahara central, Algérie). Anthropologie. 2016; 120: 263-84.
34. Geraads D, Hublin JJ, Jaeger JJ, Tong H, Sen S, Toubeau P. The Pleistocene hominid site of Ternifine, Algeria: new results on the environment, age, and human industries. Quat Res. 1986; 25: 380-6.
35. Sahnouni M, de Heinzelin J. The site of Ain Hanech revisited: new investigations at this Lower Pleistocene site in Northern Algeria. J Archaeol Sci. 1998; 25: 1083-101.
36. Sahnouni M. Les plus vieilles traces d'occupation humaine en Afrique du Nord : perspectives de l'Ain Hanech, Algérie. C R Palevol. 2006; 5: 243-54.
37. Mesfin I. Les assemblages lithiques lupembiens conservés au Muséum National d'Histoire Naturelle : apports et perspectives pour la connaissance du *Middle Stone Age* d'Afrique centrale [master thesis]. Paris, France: Muséum

National d'Histoire Naturelle; 2018.

38. Le Tensorer JM, Von Falkenstein V, Le Tensorer H, Schmid P, Muhesen S. Etude préliminaire des industries archaïques de faciès Oldowayen du site de Hummal (El Kowm, Syrie centrale). Anthropologie. 2011; 115: 247-66.

39. Martínez-Navarro B, Belmaker M, Bar-Yosef O. The large carnivores from 'Ubeidiya (early Pleistocene, Israel): biochronological and biogeographical implications. J Hum Evol. 2009; 56: 514-24.

40. Güleç E, White T, Kuhn S, Özer I, Sagir M, Yilmaz H et al. The Lower Pleistocene lithic assemblage from Dursunlu (Konya), central Anatolia, Turkey. Antiquity 2009 Mar; 83(319): 11-22.

41. Sharon G, Feibel C, Alperson-Afil N, Harlavan Y, Feraud G, Ashkenazi S et al. New evidence for the Northern Dead Sea rift Acheulian. PaleoAnthropology Society 2010: 79-99.

42. Bar-Yosef O. The Lower Paleolithic of the Near East. J World Prehist. 1994; 8(3): 211-65.

43. Yazbeck C. Les systèmes techniques de production au Paléolithique inférieur en Beqaa Libanaise : le cas de Joubb Jannine II [doctoral thesis]. Lyon, France: Université Lumière Lyon 2; 2002.

44. Marder O, Gvirtzman G, Ron H, Khalaily H, Wieder M, Bankirer R et al. The lower Paleolithic site of Revadim Quarry, preliminary finds. Journal of the Israel Prehistoric Society 1999; 28: 21-53.

45. Solodenko N, Zupancich A, Nunziante Cesaro S, Marder O, Lemorini C, Barkai R. Fat residue and use-wear found on Acheulian biface and scraper associated with butchered elephant remains at the site of Revadim, Israel. PloS One. 2015; 10(3): e0118572.

46. Porat N, Ronen A. Luminescence and ESR age determinations of the Lower Paleolithic site Evron Quarry, Israel. Advances in ESR applications 2002; 18: 123-30.

47. Scerri EML, Shipton C, Clark-Balzan L, Frouin M, Schwenninger JL, Groucutt HS et al. The expansion of later Acheulean hominins into the Arabian Peninsula. Sci Reports. 2018; 8: 17165.

48. Barkai R, Gopher A, Lauritzen SE, Frumkin A. Uranium series dates from Qesem Cave, Israel, and the end of the Lower Palaeolithic. Nature 2003; 423: 977-9.

49. Gopher A, Ayalon A, Bar-Matthews M, Barkai R, Frumkin A, Karkanas P et al. The chronology of the Late Lower Paleolithic in the Levant based on U-Th ages of speleothems from Qesem cave, Israel. Quat Geochronol. 2010; 5(6): 644-56.

50. Mercier N, Valladas H, Falguères C, Shao Q, Gopher A, Barkai R et al. New datings of Amudian layers at Qesem Cave (Israel): results of TL applied to burnt flints and ESR/U-series to teeth. J Archaeol Sci. 2013; 40:3011-20.

51. Falguères C, Richard M, Tombret O, Shao Q, Bahain JJ, Gopher A et al. New ESR/U-series dates in Yabrudian and Amudian layers at Qesem Cave, Israel. Quat Int. 2016; 398: 6-12.

52. Barkai R, Gopher A. On anachronism: the curious presence of spheroids and polyhedrons at Acheulo–Yabrudian Qesem Cave, Israel. Quat Int. 2016; 398: 118–28.

53. Feraud G, York D, Hall CM, Goren N, Schwarcz HP. $^{40}Ar/^{39}Ar$ age limit for an Acheulian site in Israel. Nature 1983 Jul; 304: 263-5.

54. Slimak L, Kuhn SL, Roche H, Mouralis D, Buitenhuis H, Balkan-Atli N et al. Kaletepe Deresi 3 (Turkey): archaeological evidence for early human settlement in Central Anatolia. J Hum Evol. 2008; 54: 99-111.

55. Slimak L, Kuhn SL, Balkan-Atli N, Binder D, Grenet M, Dinçer B. Kaletepe Deresi 3: de l'Acheuléen au Moustérien en Anatolie Centrale. Anatolia Antiqua, tome 15. 2007. p. 257-73.

56. Barsky D, Vergès JM, Sala R, Menéndez L, Toro-Moyano I. Limestone percussion tools from the late Early Pleistocene sites of Barranco León and Fuente Nueva 3 (Orce, Spain). Philos Trans R Soc Lond B. 2015; 370: 20140352.

57. Bourguignon L, Barsky D, Ivorra J, de Weyer L, Cuartero F, Capdevila R et al. The stone tools from stratigraphical unit 4 of the Bois-de-Riquet site (Lézignan-la-Cèbe, Hérault, France): a new milestone in the diversity of the European Acheulian. Quat Int. 2016; 411: 160-81.

58. Terradillos Bernal M, Moncel MH. Contribution à l'étude de la technologie du Paléolithique « archaïque » du sud de l'Europe selon le Système Logique Analytique (SLA). Application aux sites du Vallonnet (Roquebrune-Cap-Martin, France), de Gran Dolina TD6 (Burgos, Espagne), de Ca'Belvedere de Monte Poggiolo (Forli, Italie) et de Barranco Léon et Fuente Nueva 3 (Orce, Espagne). Anthropologie. 2004; 108: 307-29.

59. Fiedler L, Humburg C, Klingelhöfer H, Stoll S, Stoll M. Several Lower Palaeolithic sites along the Rhine Rift Valley, dated from 1.3 to 0.6 million years. Humanities 2019; 8(129).

60. Iovita R, Tuvi-Arad I, Moncel MH, Despriée, Voinchet P, Bahain JJ. High handaxe symmetry at the beginning of the European Acheulian: the data from la Noira (France) in context. PloS One. 2017; 12(5): e0177063.

61. Barsky D. Le débitage des industries lithiques de la Caune de l'Arago (Pyrénées-Orientales, France) : leur place dans l'évolution des industries du Paléolithique inférieur en Europe méditerranéenne [doctoral thesis]. Perpignan, France: Université de Perpignan; 2001.

62. Doronichev VB, Golovanova L. Beyond the Acheulean: a view on the Lower Paleolithic occupation of Western Eurasia. Quat Int. 2010; 223: 327-44.

63. Colonge D. Aquitaine, A65, Pyrénées-Atlantiques, Auriac, Duclos : Pléistocène moyen et Antiquité en Béarn. Inrap Grand Sud-Ouest; 2012. 476 p.

64. Bahain JJ, Duval M, Voinchet P, Tissoux H, Falguères C, Grün R et al. ESR and ESR/U-series chronology of the Middle Pleistocene site of Tourville-la-Rivière (Normandy, France) - A multi-laboratory approach. Quat Int. 2020; 556: 66-78.

65. Richter D, Hublin JJ, Jaubert J, McPherron SP, Soressi M, Texier JP. Thermoluminescence dates for the Middle Palaeolithic site of Chez-Pinaud Jonzac (France). J Archaeol Sci. 2013; 40(2): 1176-85.

66. Debénath A, Jelinek AJ, Armand D, Chase PG, Dibble HL, Mercier N et al. Nouvelles fouilles à La Quina (Charente) : résultats préliminaires. Gallia Préhistoire 1998; 40: 29-74.

67. Pittard E, Donici A. Les pierres de jet d'une station intermédiaire entre le Moustérien et l'Aurignacien (Dordogne). L'Homme Préhistorique 1927 Sep-Oct; 14(9-10): 209-19.

68. Collina-Girard J. Grille descriptive et évolution typologique des industries archaïques : le modèle catalan. Bulletin de la Société préhistorique française. 1986; 83(11-12): 383-403.

69. Garcia Garriga J. Las industrias arcaicas del Rosellón (sur de Francia): correlación geocronológica con las terrazas de los ríos Têt, Tech y Agly. Zephyrus 2014 Jan-Jun; 73: 17-43.

70. Collina-Girard J. Les industries archaïques sur galets des terrasses quaternaires de la Têt et du Tech (Catalogne française). Géologie Méditerranéenne 1976; 3(3): 183-90.

71. Delmas M, Calvet M, Gunnell Y, Voinchet P, Manel C, Braucher R et al. Terrestrial 10Be and electron spin resonance dating of fluvial terraces quantifies quaternary tectonic uplift gradients in the eastern Pyrenees. Quat Sci Rev. 2018; 193: 188-211.

72. Gaillard C, Rajaguru SN. Revisiting the Acheulian site of Singi Talav at Didwana (Rajasthan) 35 years later. In: Deo SG, Baptista A, Joglekar J, editors. Rethinking the past: a tribute to Professor V.N. Misra. Indian Society for Prehistoric and Quaternary Studies; 2017. p. 25-39.

73. Shen G, Gao X, Gao B, Granger DE. Age of Zhoukoudian *Homo erectus* determined with $^{26}Al/^{10}Be$ burial dating. Nature 2009 Mar; 458: 198-200.

74. Li Y, Sun X, Bodin E. A macroscopic technological perspective on lithic production from the Early to Late Pleistocene in the Hanshui River Valley, central China. Quat Int. 2014; 347: 148-62.

75. Chen TM, Yuan SX, Gao SJ. The study on uranium-series dating of fossil bones and an absolute age sequence for the main Paleolithic sites of North China. Acta Anthropol Sinica. 1984; 3(3): 259-69.

76. Ma XH, Qian FL, Li P, Ju SQ. Palaeomagnetic dating of Lantian man. Vertebrata PalAsiatica 1978; 16(4): 238-43.

77. Cheng GL, Lin JL, Li SL. A research on the ages of the strata of “Lantian man”. Collected papers of palaeoanthropology. Beijing, China: Science Press Beijing; 1978. p. 151-7.

78. An ZS, Ho CK. New magnetostratigraphic dates of Lantian *Homo erectus*. Quat Res. 1989; 32: 213-21.

79. Pei S, Niu D, Guan Y, Nian X, Yi M, Ma N et al. Middle Pleistocene hominin occupation in the Danjiangkou Reservoir region, Central China: studies of formation processes and stone technology of Maling 2A site. J Archaeol Sci. 2015; 53: 391-407.

80. Li X, Ao H, Dekkers MJ, Roberts AP, Zhang P, Lin S et al. Early Pleistocene occurrence of Acheulian technology in North China. Quat Sci Rev. 2017; 156: 12-22.

81. Gao X. Explanations of typological variability in Paleolithic remains from Zhoukoudian locality 15, China [doctoral thesis]. Tucson, United States of America: University of Arizona; 2000.

82. Yang SX, Deng CL, Zhu RX, Petraglia MD. The Paleolithic in the Nihewan Basin, China: evolutionary history of an early to late Pleistocene record in Eastern Asia. Evol Anthrop. 2019; 29: 125-42.

83. Li H, Li ZY, Gao X, Kuman K, Summer A. Technological behaviour of the early Late Pleistocene archaic humans at Lingjing (Xuchang, China). Archaeol Anthropol Sci. 2019; 11(7): 3477-90.

84. Wang S, Lu H, Zhang H, Sun X, Yi S, Chen Y et al. Newly discovered Palaeolithic artefacts from loess deposits and their ages in Lantian, central China. Chin Sci Bull. 2014; 59(7): 651-61.

85. Zhuo H, Lu H, Wang S, Ahmad K, Sun W, Zhang H et al. Chronology of newly-discovered Paleolithic artIfact assemblages in Lantian (Shaanxi province), central China. Quat Res. 2016; 86: 316-25.

86. Wang SJ. Perspectives on hominid behaviour and settlement patterns: a study of the Lower Palaeolithic sites in the Luonan Basin, China. BAR International series 1406. Oxford: Archaeopress; 2005. 248 p.

87. De Lumley H, Cauche D, Celiberti V, Khatib S, Lartigot-Campin AS, Lebatard AE et al. Les industries du Paléolithique ancien de Corée du Sud dans leur contexte stratigraphique et paléoécologique: leur place parmi les cultures du Paléolithique ancien en Eurasie et en Afrique. Paris: CNRS éditions; 2011. 631 p.

88. Fauzi MR, Ansyori MM, Prastiningtyas D, Intan MFS, Wibowo UP, Wulandari et al. Matar: a forgotten but promising Pleistocene locality in East Java. Quat Int. 2016; 416: 183-92.

89. Bartstra GJ, Soegondho S, Van der Wijk A. Ngandong man: age and artifacts. J Hum Evol. 1988; 17(3): 325-37.
