# Supplementary material for: Palaeolithic polyhedrons, spheroids and bolas over time and space
Source: PLoS One. 2022 Jul 28;17(7):e0272135. doi: 10.1371/journal.pone.0272135 (PMC9333226; doi:10.1371/journal.pone.0272135)
Supplement: S3 Table — (PDF) [file pone.0272135.s003.pdf]

S3 Table. Types of sites of the corpus and their potential functions.

Key of reading:

Names of assemblage in dark grey cells: assemblages for which we considered some objects as PSBs but most of authors that worked on the site did not (e.g., they could consider it as multifacial cores).

Light grey cells: the information in the cell is very probable but not certain, or is incomplete.

NA: no information collected.

Site	Type of site	Type of occupation, activities	Comments - Types of occupation, activities
**Ewass Oldupa (Phase II)**	Open air	NA	NA
**Ewass Oldupa (Phase III)**	Open air	NA	NA
**Olduvai DK (Bed I)**	Open air	NA	NA
**Olduvai FLK North (Bed I)**	Open air	NA	NA
**Olduvai HWK-EE (Clay Unit, Bed II)**	Open air	Butchery	Pante & de la Torre 2018 [1]: majority of limb bones intentionally broken to access the marrow.
**Olduvai HWK-EE (SC Unit, Bed II)**	Open air	Butchery	Pante & de la Torre 2018 [1]: majority of limb bones intentionally broken to access the marrow.
**Olduvai SHK Main Site (Level A & B Bed II)**	Open air	Butchery	Domínguez-Rodrigo et al. 2014 [2]: several activities. Butchery was a minor activity at the site. Cut and percussion marks on some bones.
**Olduvai BK (Level 1 to 3, Bed II)**	Open air	Butchery	Butchery activities for a long period of time (Diez-Martín et al. 2009 [3], Yravedra et al. 2017 [4]).
**Olduvai BK (Level 4, Bed II)**	Open air	Butchery	Butchery activities for a long period of time (Diez-Martín et al. 2009 [3], Yravedra et al. 2017 [4]).
**Olduvai TK (Bed II)**	Open air	Butchery, Plant processing	Experiments of Santonja et al. 2014 [5]: plant processing, bone-breaking, bone-marrow extraction.
**Melka Kunture (Karre Level K1-2)**	Open air	NA	NA
**Melka Kunture (Garba IV)**	Open air	Butchery, Knapping	Piperno 2001 [6]: "activities dealing with meat sharing and consumption"; knapping tools in obsidian.
**Melka Kunture (Gombore IB)**	Open air	NA	NA
**Melka Kunture (Gombore II, Locality 1)**	Open air	NA	NA
**Melka Kunture (Gombore II, Locality 2)**	Open air	Butchery	Possible hippo butchery site (Chavaillon & Berthelet 2004 [7]).
**Melka Kunture (Gombore II, Locality 3)**	Open air	Recurrent occupation	Chavaillon & Berthelet 2004 [7]

**Melka Kunture (Gombore II, Locality 4)**	Open air	Recurrent occupation	Chavaillon & Berthelet 2004 [7]
**Melka Kunture (Gombore II, Locality 5)**	Open air	Recurrent occupation	Chavaillon & Berthelet 2004 [7]
**Melka Kunture (Gombore Iγ)**	Open air	Short occupation	Chavaillon et Berthelet 2004 [7]
**Melka Kunture (Simbiro III, level A)**	Open air	NA	NA
**Melka Kunture (Simbiro III, level B)**	Open air	NA	NA
**Melka Kunture (Simbiro III, level D)**	Open air	NA	NA
**Barogali**	Open air	Butchery, Scavenging, Knapping	Hominins knapped tools *in situ* during the scavenging process (Berthelet 2001 [8]).
**Gadeb 2B**	Open air	Recurrent occupation	Gadeb localities except 8F (de la Torre 2011 [9], citing Clark 1987 [10]): probably “regular re-occupation”.
**Gadeb 2C**	Open air	Recurrent occupation	Gadeb localities except 8F (de la Torre 2011 [9], citing Clark 1987 [10]): probably “regular re-occupation”.
**Gadeb 2E**	Open air	Recurrent occupation	Gadeb localities except 8F (de la Torre 2011 [9], citing Clark 1987 [10]): probably “regular re-occupation”.
**Gadeb 8A**	Open air	Recurrent occupation	Gadeb localities except 8F (de la Torre 2011 [9], citing Clark 1987 [10]): probably “regular re-occupation”.
**Gadeb 8F**	Open air	Butchery	Hippo butchery site (Clark and Kurashina 1979 [11].
**Isenya (level V)**	Open air	Short occupation, Butchery, Knapping	Short occupation : "occupations temporaires : installation lors des étiages" (Roche et al. 1988 [12]). Knapping activities. Faunal remains brought to the site by hominins; anthropical bone breakage (Roche et al. 1988 [12], Clément 2019 [13]).
**Isenya (level VIa)**	Open air	Short occupation, Butchery, Knapping	Short occupation : "occupations temporaires : installation lors des étiages" (Roche et al. 1988 [12]). Knapping activities. Faunal remains brought to the site by hominins; anthropical bone breakage (Roche et al. 1988 [12], Clément 2019 [13]).

**Isenya (level VIb21)**	Open air	Short occupation, Butchery, Knapping	Short occupation : "occupations temporaires : installation lors des étiages" (Roche et al. 1988 [12]). Knapping activities. Faunal remains brought to the site by hominins; anthropical bone breakage (Roche et al. 1988 [12], Clément 2019 [13]).
**Olorgesailie Member 2**	Open air	NA	Butchery activities attested at Olorgesailie (Clément 2019 [13]).
**Olorgesailie Member 3**	Open air	NA	Butchery activities attested at Olorgesailie (Clément 2019 [13]).
**Olorgesailie Member 6**	Open air	NA	Butchery activities attested at Olorgesailie (Clément 2019 [13]).
**Olorgesailie Member 7**	Open air	NA	Butchery activities attested at Olorgesailie (Clément 2019 [13]).
**Olorgesailie Member 8**	Open air	NA	Butchery activities attested at Olorgesailie (Clément 2019 [13]).
**Olorgesailie Member 9**	Open air	NA	Butchery activities attested at Olorgesailie (Clément 2019 [13]).
**Olorgesailie Member 10**	Open air	NA	Butchery activities attested at Olorgesailie (Clément 2019 [13]).
**Olorgesailie Member 11**	Open air	NA	Butchery activities attested at Olorgesailie (Clément 2019 [13]).
**Isimila (K6)**	Open air	Short occupation	Butchery activities attested at Isimila (Clément 2019 [13]). "only brief, perhaps seasonal encampments (and workshops in some cases)" (Howell et al. 1962 [14]).
**Isimila (LJ6-7)**	Open air	Short occupation	Butchery activities attested at Isimila (Clément 2019 [13]). "only brief, perhaps seasonal encampments (and workshops in some cases)" (Howell et al. 1962 [14]).
**Isimila (K14)**	Open air	Short occupation	Butchery activities attested at Isimila (Clément 2019 [13]). "only brief, perhaps seasonal encampments (and workshops in some cases)" (Howell et al. 1962 [14]).
**Isimila (H9-38)**	Open air	Short occupation	Butchery activities attested at Isimila (Clément 2019 [13]). "only brief, perhaps seasonal encampments (and workshops in some cases)" (Howell et al. 1962 [14]).
**Ounjougou**	Open air	NA	NA
**Kabwe (Broken Hill)**	Cave	NA	NA
**Broken Hill (Sangoan)**	Open air	NA	NA
**Broken Hill (Rhodesian Acheulian)**	Open air	Bone processing, or Wood processing	The "nature of the working" on flake edges and nodule tools suggest work on "hard substances" (wood or bone) (Clark 1959 [15]).
**Broken Hill (Hope Fountain Bariant)**	Open air	Bone processing, or Wood processing	The "nature of the working" on flake edges and nodule tools suggest work on "hard substances" (wood or bone) (Clark 1959 [15]).
**Kalambo Falls (A4 rubble [illegible]**	Open air	NA	NA
**Kalambo Falls (A4 rubble [illegible]**	Open air	NA	NA
**Kalambo Falls (A5 Comp)**	Open air	Habitat	"These Acheulian living floors represent dry season camp sites." (Clark 1981 [15]).

**Cave of Hearths (Bed 1-3)**	Cave	Butchery, Knapping	Butchery traces on bones, as notches and pits resulting from percussion activity with hammerstone. Knapping (McNabb et al. 2009 [16]).
**Swartkrans (SWT-M1, SPRP excavation)**	Cave	Butchery	Kuman et al. 2018 [17]: lower bank of M1 fauna records "contains the oldest evidence for hominid butchery of large vertebrate carcasses in southern Africa (Pickering et al. 2008)".
**Swartkrans (SWT-M1, LB)**	Cave	Butchery	Kuman et al. 2018 [17]: lower bank of M1 fauna records "contains the oldest evidence for hominid butchery of large vertebrate carcasses in southern Africa (Pickering et al. 2008)".
**Swartkrans (SWT-M2)**	Cave	NA	NA
**Swartkrans (SWT-M3)**	Cave	Butchery	"[…] evidence of fire and butchery" (Clark 1993 [18]).
**Vlakkraal Thermal springs**	Open air	NA	NA
**Windhoek**	Open air	NA	NA
**Esere**	Open air	NA	NA
**Rhino Cave (Tsodilo Hills)**	Cave	NA	NA
**Corner Cave (Tsodilo Hills)**	Cave	NA	NA
**Kalkbank**	Open air	Hunting, Butchery	Maybe a predation hot spot. Human butchery use-wear (i.e. percussions marks on bovids limb bones). Mason et al. 1958 [19], Hutson & Cain 2008 [20].
**Florisbad**	Open air	NA	NA
**Sterkfontein (Member 5)**	NA	NA	NA
**El Guettar**	Open air	NA	NA
**Ain El Hallouf**	NA	NA	NA
**Sidi Abderrahmane**	Open air	NA	NA
**Sidi Abderrahmane STIC**	Open air	NA	NA
**Erg Tihodaine (Coll. Arambourg)**	Open air	Habitat, or Hunting, or Butchery	The faunal assemblage could be due to anthropic activity (but the presence of *E. recki,* Thomas 1977 [21]), according to Hocine 2016 [22]: "lieu d'habitat ou d'acquisition de ressources carnées".

**Tighennif I**	Open air	Butchery	In few cases, intentionally broken long bones probably to extract marrow. Cut marks on some bones (Geraads et al. 1986 [23]).
**Ain Hanech**	Open air	Butchery	Meat processing use-wear on flint artifacts (Sahnouni 2006 [24]).
**Nzako Ambilo**	Open air	NA	NA
**Nzako Kono**	Open air	NA	NA
**M'Piaka**	Open air	NA	NA
**Hummal (Levels 17 & 18)**	Open air	Habitat, Knapping, Butchery	Habitat, with flint knapping activities and many faunal bones records brought to the site by hominids. More details about these assemblages: e.g. Wegmüller 2011 [25], Le Tensorer et al. 2011 [26].
**Shuwayhitiyah**	Open air	NA	NA
**'Ubeidiya (III-20)**	Open air	NA	NA
**'Ubeidiya (III-22)**	Open air	NA	NA
**Dursunlu**	Open air	NA	NA
**North of Bridge Acheulian (NBA)**	Open air	NA	NA
**Latamné**	Open air	NA	NA
**Joubb Jannine II**	Open air	NA	NA
**Khalliyé Sud**	Open air	NA	NA
**Wadi Fatimah**	Open air	NA	NA
**Revadim Quarry (Area D)**	Open air	NA	Use-wear (i.e. cut marks) on bones (elephant) and fat residues on some tools, attest butchery activities (Solodenko et al. 2015 [27]). Maybe not in the same assemblage than PSBs.
**Evron Quarry**	Open air	NA	NA
**Evron East**	Open air	NA	NA
**Saffaqah**	Open air	NA	NA
**Qesem Cave**	Cave	Base camp, Knapping, Butchery	Base camp, with many different activities. Knapping. Butchery (e.g. bone breakage) attested by use-wear and residues on tools (i.e. Assaf et al. 2020 [28]).
**Bezez**	Cave	NA	NA
**Ma'ayan Barukh**	Open air	Camp site	Camp site according to Stekelis & Gilead 1966 [29].
**Kaletepe Deresi 3 (Level III)**	Open air	NA	NA
**Kaletepe Deresi 3 (Level III/IV)**	Open air	NA	NA

**Kaletepe Deresi 3 (Level IV)**	Open air	NA	NA
**Kaletepe Deresi 3 (Level V)**	Open air	NA	NA
**Kaletepe Deresi 3 (Level Vam)**	Open air	NA	NA
**Kaletepe Deresi 3 (Level V')**	Open air	NA	NA
**Kaletepe Deresi 3 (Level VI')**	Open air	NA	NA
**Kaletepe Deresi 3 (Level VII)**	Open air	NA	NA
**Kaletepe Deresi 3 (Level VIII)**	Open air	NA	NA
**Kaletepe Deresi 3 (Level IX)**	Open air	NA	NA
**Kaletepe Deresi 3 (Level X)**	Open air	NA	NA
**Kaletepe Deresi 3 (Level XI)**	Open air	NA	NA
**Kaletepe Deresi 3 (Level XII)**	Open air	NA	NA
**Santa Ana Cave**	Cave	NA	NA
**Barranco León**	Open air	Butchery, Knapping, Wood processing, Plant processing	Knapping (lithic refitting), butchery (traces on large mammal bones), maybe wood and plant treatment. Synthesis: Titton 2020 [30]).
**Bois-de-Riquet (Unit 4)**	NA	NA	NA
**Ca' Belvedere di Monte Poggiolo**	Open air	Knapping, Butchery, Wood processing	Butchery (more generally, exploitation of animal resources; knapping (Terradillos-Bernal & Moncel 2004 [31]). Use-wear on lithics also indicate work on wood.
**Dorn-Dürkheim 3**	Open air	NA	NA
**La Noira (Stratum c)**	Open air	NA	NA
**Caune de l'Arago (Unit H1,2,3)**	Cave	Seasonal habitat	Seasonal habitat of cervid hunters (Barsky 2001 [32]).
**Caune de l'Arago (Unit G)**	Cave	Long occupation	Occupant may have been big herbivore hunters (Barsky 2001 [32]).
**Caune de l'Arago (Unit E)**	Cave	Seasonal habitat	Occupant may have been mouflon hunters (Barsky 2001 [32]).
**Caune de l'Arago (Unit D)**	Cave	Seasonal habitat, Butchery	Seasonal habitat of hunters (Barsky 2001 [32]). Long bone broken for marrow extraction (Barsky et al. 2019 [33]).
**Treugol'Naya Cave (assemblage II)**	Cave	NA	NA
**Duclos (0)**	Open air	Residential, Butchery, Wood processing	Many activities performed on the site, as carcasses treatment, disarticulation, maybe bone fracturing, acquisition and/or treatment of vegetal wood (Cologne 2012 [34]).
**Duclos (Ensemble IV)**	Open air	Residential, Butchery, Wood processing	Many activities performed on the site, as carcasses treatment, disarticulation, maybe bone fracturing, acquisition and/or treatment of vegetal wood (Cologne 2012 [34]).

**Duclos (Ensemble III)**	Open air	Residential, Butchery, Wood processing	Many activities performed on the site, as carcasses treatment, disarticulation, maybe bone fracturing, acquisition and/or treatment of vegetal wood (Colonge 2012 [34]).
**Septsos**	Open air	Habitat, Knapping, Butchery, Wood processing	Probably butchery activities (disarticulation, percussion on bones and/or carcasses), knapping, processing of semi-hard material as wood. The morpho-technical and functional diversity of the industries coupled with the potential of the habitat suggests a generalist habitat, possibly residential (Fourloubey 2012 [35]).
**Cerveny Kopec**	NA	NA	NA
**Bañugues (Asturias del Esferoid)**	Open air	NA	NA
**Tourville-la-Rivière (level D2)**	Open air	Hunting, Butchery, Scavenging	Cliquet 2010 [36].
**Chez-Pinaud Jonzac (US 22)**	Shelter	Butchery, Knapping	Butchery activities (cutting and percussion) and work of fresh skin. Possible workshop of primary treatment of carcasses (Claud et al. 2012 [37]). Roussel et al. 2009 [38] argue that the "boules calcaires" are hammers, maybe to knap scrapers in flint.
**La Quina (level 8)**	Open air	Butchery	Fracturing of reindeer carcasses, probably to extract marrow. Meat-rich parts may have been transported elsewhere (Chase 1999 [39], Park 2007 [40]).
**Festons (Rebières valley)**	Shelter	NA	NA
**Sablière Rambour (Villers-Bocage)**	NA	NA	NA
**Isle-Adam (sablière de Cassan)**	NA	NA	NA
**Coll de la Guille (Terrasses du Roussillon)**	Open air	NA	NA
**Mas Ferreol (Terrasses du Roussillon)**	Open air	NA	NA
**Mas Ferrer (Terrasses du Roussillon)**	Open air	NA	NA
**Le Puech de la Boule (Terrasses du Roussillon)**	Open air	NA	NA
**Mas Bruno (Terrasses du Roussillon)**	Open air	NA	NA
**Cabestany général (Terrasses du Roussillon)**	Open air	NA	NA
**La Llabanère (Terrasses du Roussillon)**	Open air	NA	NA
**Singi Talav (Layer 3)**	Open air	Living site	Gaillard et al. 1985 [41]: "probably not a workshop but a living area where various activities took place".
**Singi Talav (Layer 4)**	Open air	Living site	Gaillard et al. 1985 [41]: "probably not a workshop but a living area where various activities took place".
**Torajunga**	Open air	NA	NA
**Chirki Nevasa**	Open air	Living site, Knapping	Living and factory site (Corvinus 1983) [42].

**Atit 2**	Open air	NA	NA
**Zhoukoudian 1 (Layer 1-3)**	Cave	NA	NA
**Zhoukoudian 1 (Layer 4-5)**	Cave	NA	NA
**Zhoukoudian 1 (QII)**	Cave	NA	NA
**Zhoukoudian 1 (Layer 8-9)**	Cave	NA	NA
**Liangshan Longgangsi**	Open air	NA	NA
**Dingcun**	Open air	NA	NA
**Gongwangling**	Open air	NA	NA
**Ganyu**	Open air	NA	NA
**Maling 2A**	Open air	NA	NA
**Shuigou-Huixinggou**	NA	NA	NA
**Zhoukoudian 15**	Open air	NA	NA
**Xujiayao**	Open air	Kill site, Scavenging	Horse kill site (i.e. Li et al. 2017 [43]), but also scavenging (Yang et al. 2019 [44]).
**Lingjing (Layer 11, lower part of layer 10)**	Open air	Kill site, Butchery	Zhang et al. 2011 [45]: "Middle Paleolithic kill-butchery site rather than a home base for early humans".
**Hsuchiyao**	NA	Butchery, Hunting	Faunal bones may have been intentionally fragmented in order to access marrow (Chi 1979 [46]).
**Diaozhai**	Open air	NA	NA
**Jijiawan**	Open air	NA	NA
**Houjiapu**	Open air	NA	NA
**Zhoupo (Locality 95LP07)**	Open air	NA	NA
**Mansuri (Locality 1)**	NA	NA	NA
**Jeongok-Ri (surface)**	Open air	NA	NA
**Jeongok-Ri (Layer 1)**	Open air	NA	NA
**Jeongok-Ri (Layer 2)**	Open air	NA	NA
**Jeongok-Ri (Layer 3)**	Open air	NA	NA
**Jangnamgyo (surface)**	Open air	NA	NA
**Jangnamgyo (Level 3)**	Open air	NA	NA
**Ngebung**	Open air	NA	NA
**Banjarejo**	Open air	NA	NA
**Matar**	Open air	NA	NA
**Solo**	Open air	NA	NA
**Baksoko River**	Open air	NA	NA

References

1. Pante MC, de la Torre I. A hidden treasure of the Lower Pleistocene at Olduvai Gorge, Tanzania: the Leakey HWK EE assemblage. J Hum Evol. 2018; 120: 114-39.
2. Domínguez-Rodrigo M, Diez-Martín F, Yravedra J, Barba R, Mabulla A, Baquedano E et al. Study of the SHK main site faunal assemblage, Olduvai Gorge, Tanzania: implications for Bed II taphonomy, paleoecology, and hominin utilization of megafauna. Quat Int. 2014; 322-323: 153-66.
3. Diez-Martín F, Sánchez P, Domínguez-Rodrigo M, Mabulla A, Barba R. Were Olduvai hominins making butchering tools or battering tools? Analysis of a recently excavated lithic assemblage from BK (Bed II, Olduvai Gorge, Tanzania). J Anthropol Archaeol. 2009; 28(3): 274-89.
4. Yravedra J, Maté-González MA, Palomeque-González JF, Aramendi J, Estaca-Gómez V, San Juan Blazquez M et al. A new approach to raw material use in the exploitation of animal carcasses at BK (Upper Bed II, Olduvai Gorge, Tanzania): a micro-photogrammetric and geometric morphometric analysis of fossil cut marks. Boreas. 2017; 46: 860-73.
5. Santonja M, Panera J, Rubio-Jara S, Pérez-González A, Uribelarrea D, Domínguez-Rodrigo M et al. Technological strategies and the economy of raw materials in the TK (Thiongo Korongo) lower occupation, Bed II, Olduvai Gorge, Tanzania. Quat Int. 2014; 322-323: 181-208.
6. Piperno M. The prehistory of Melka Kunture (Ethiopia). Bulletin du CRFJ. 2001; 8: 135-45.
7. Chavaillon J, Berthelet A. The archaeological sites of Melka Kunture. In: Chavaillon J, Piperno M, editors. Studies on the Early Paleolithic site of Melka Kunture, Ethiopia. Florence: Istituto Italiano di Preistoria e Protostoria; 2004. p. 25-80.
8. Berthelet A. L’outillage lithique du site de dépeçage à *Elephas recki ileretensis* de Barogali (République de Djibouti). C R Acad Sci IIa. 2001 Mar; 332(6): 411-6.
9. De la Torre I. The Early Stone Age lithic assemblages of Gadeb (Ethiopia) and the Developed Oldowan/early Acheulean in East Africa. J Hum Evol. 2011; 60: 768-812.
10. Clark JD. Transitions: *Homo erectus* and the Acheulian: the Ethiopian sites of Gadeb and the middle Awash. J Hum Evol. 1987; 16: 809-26.
11. Clark JD, Kurashina H. Hominid occupation of the East-Central Highlands of Ethiopia in the Plio-Pleistocene. Nature 1979 Nov; 282: 33-9.
12. Roche H, Brugal JP, Lefevre D, Ploux S, Texier PJ. Isenya: état des recherches sur un nouveau site acheuléen d’Afrique orientale. Afr Archaeol Rev. 1988; 6(1): 27-55.
13. Clément S. Les techniques de percussion : un reflet des changements techniques durant l'Acheuléen ? [doctoral thesis]. Nanterre, France: Université Paris X; 2019.
14. Howell FC, Cole GH, Kleindienst MR, Haldemann EG. Isimila: an Acheulian occupation site in the Iringa Highlands, Southern Highlands Province, Tanganyika. Musée Royal de l’Afrique centrale 1962; 43-81.
15. Clark JD. Further excavations at Broken Hill, Northern Rhodesia. The Journal of the Royal Anthropological Institute of Great Britain and Ireland 1959 Jul-Dec; 89(2): 201-32.
16. McNabb J, Sinclair A., Wadley L, Maguire J, Latham A, Herries A et al. The Cave of Hearths: Makapan Middle Pleistocene research project: field research by Antony Sinclair and Patrick Quinney, 1996-2001. Oxford: Archaeopress; 2009. 193 p.
17. Kuman K, Sutton MB, Pickering TR, Heaton JL. The Oldowan industry from Swartkrans cave, South Africa, and its relevance for the African Oldowan. J Hum Evol. 2018; 123: 52-69.
18. Clark JD. Stone artefact assemblages from Members 1-3, Swartkrans Cave. In: Brain C, editor. Swartkrans: a cave’s chronicle

of early man. Transvaal Museum Monograph No. 8. Pretoria: Transvaal Museum; 1993. 167-94.

19. Mason RJ. Bone tools at the Kalkbank Middle Stone Age site and the Makapansgat Australopithecine locality, central Transvaal. Part 1: the Kalbank site. The South African Archaeological Bulletin 1958; 13: 85-93.

20. Hutson JM, Cain CR. Reanalysis and reinterpretation of the Kalkbank faunal accumulation, Limpopo Province, South Africa. Journal of Taphonomy 2008; 6(3-4): 399-428.

21. Thomas H. Géologie et Paléontologie du gisement acheuléen de l'Erg Tihodaïne (Ahaggar, Sahara central). Mém Cent rech anthropol préhist ethnogr. (Alger). 1977; 27.

22. Hocine S. Le site acheuléen d'Erg Tihodaïne : caractéristiques technologiques de l'industrie lithique du Pléistocène moyen (Sahara central, Algérie). Anthropologie. 2016 Jun; 120(3): 263-84.

23. Geraads D, Hublin JJ, Jaeger JJ, Tong H, Sen S, Toubeau P. The Pleistocene hominid site of Ternifine, Algeria: new results on the environment, age, and human industries. Quat Res. 1986; 25: 380-6.

24. Sahnouni M. Les plus vieilles traces d'occupation humaine en Afrique du Nord : perspectives de l'Ain Hanech, Algérie. C R Palevol. 2006; 5: 243-54.

25. Wegmüller F. The Lower Palaeolithic assemblage of Hummal. Le Tensorer JM, Jagher R, Otte M, editors. The Lower and Middle Palaeolithic in the Middle East and Neighbouring Regions. Basel Symposium (May 8-10 2008). Liège: ErAul 126; 2011. p. 271-8.

26. Le Tensorer JM, Von Falkenstein V, Le Tensorer H, Schmid P, Muhesen S. Etude préliminaire des industries archaïques de faciès Oldowayen du site de Hummal (El Kowm, Syrie centrale). Anthropologie. 2011; 115: 247-66.

27. Solodenko N, Zupancich A, Nunziante Cesaro S, Marder O, Lemorini C, Barkai R. Fat residue and use-wear found on Acheulian biface and scraper associated with butchered elephant remains at the site of Revadim, Israel. PloS One. 2015; 10(3): e0118572.

28. Assaf E, Caricola I, Gopher A, Rosell J, Blasco R, Bar O, et al. Shaped stone balls were used for bone marrow extraction at Lower Paleolithic Qesem Cave, Israel. PLoS One. 2020; 15(4): e0230972.

29. Stekelis M, Gilead D. Ma'ayan Barukh: a Lower Palaeolithic site in Upper Galilee. Mitekufat Haeven: Journal of the Israel Prehistoric Society 1966; 1-23.

30. Titton S. Lithic assemblage, percussive technologies and behaviour at the Oldowan site of Barranco León (Orce, Andalucía, Spain) [doctoral thesis]. Tarragona, Spain: Universitat Rovira I Virgili; 2020.

31. Terradillos Bernal M, Moncel MH. Contribution à l'étude de la technologie du Paléolithique « archaïque » du sud de l'Europe selon le Système Logique Analytique (SLA). Application aux sites du Vallonnet (Roquebrune-Cap-Martin, France), de Gran Dolina TD6 (Burgos, Espagne), de Ca'Belvedere de Monte Poggiolo (Forli, Italie) et de Barranco Léon et Fuente Nueva 3 (Orce, Espagne). Anthropologie. 2004; 108: 307-29.

32. Barsky D. Le débitage des industries lithiques de la Caune de l'Arago (Pyrénées-Orientales, France) : leur place dans l'évolution des industries du Paléolithique inférieur en Europe méditerranéenne [doctoral thesis]. Perpignan, France: Université de Perpignan; 2001.

33. Barsky D, Moigne AM, Pois V. The shift from typical Western European Late Acheulian to microproduction in unit 'D' of the late Middle Pleistocene deposits of the Caune de l'Arago (Pyrénées-Orientales, France). J Hum Evol. 2019; 135: 102650.

34. Colonge D. Aquitaine, A65, Pyrénées-Atlantiques, Auriac, Duclos : Pléistocène moyen et Antiquité en Béarn. Inrap Grand Sud-Ouest; 2012. 476 p.

35. Fourloubey C. Aquitaine, A65, Landes, Cazères-sur-l'Adour: Septsos. Inrap Grand Sud-Ouest; 2012. 278 p.

36. Cliquet D. Tourville-la-Rivière, Seine-Maritime : carrières et ballastières de Normandie : la Fosse-Marmitaine. Inrap Grand-Ouest; 2010. 105 p.

37. Claud É, Soressi M, Jaubert J, Hublin JJ. Étude tracéologique de l'outillage moustérien de type Quina du bonebed de Chez-Pinaud à Jonzac (Charente-Maritime). Nouveaux éléments en faveur d'un site de boucherie et de traitement des peaux. Gallia Préhistoire 2012; 54(2): 3-32.

38. Roussel M, Bourguignon L, Soressi M. Identification par l'expérimentation de la percussion au percuteur de calcaire au Paléolithique moyen : le cas du façonnage des racloirs bifaciaux Quina de Chez Pinaud (Jonzac, Charente-Maritime). Bulletin de la Société préhistorique française 2009 Apr; 106(2): 219-38.

39. Chase P. Bison in the context of complex utilization of faunal resources: a preliminary report on the Mousterian zooarchaeology of La Quina (Charente, France). In: Brugal F, David F, Enloe JG, Jaubert J, editors. Le Bison : gibier et moyen de subsistance des hommes du Paléolithique aux Paléoindiens des grandes plaines. Juan les Pins: APDCA; 1999. p. 159-84.

40. Park SJ. Systèmes de production lithique et circulation des matières premières au Paléolithique moyen récent et final. Une approche techno-économique à partir de l'étude des industries lithiques de La Quina (Charente) [doctoral thesis]. Nanterre, France: Université Paris X; 2007.

41. Gaillard C, Misra VN, Rajaguru SN, Raju DR, Raghavan H. Acheulian occupation at Singi Talav in the Thar desert: a preliminary report on 1981 excavation. Bulletin of the Deccan College Post-Graduate and Research Institute 1985; 44: 141-52.

42. Corvinus G. A survey of the Pravara River system in western Maharashtra, India, vol. 2: the excavation of the Acheulian site of Chirki-on-Pravara, India. Tübingen: Archaeologica Vanatoria; 1983. 466 p.

43. Li JS, Bunn HT, Zhang SQ, Gao X. Equid prey acquisition and Archaic *Homo* adaptability at the early Late Pleistocene site of Xujiayao, China. Int J Osteoarchaeol. 2018; 28: 75-82.

44. Yang SX, Deng CL, Zhu RX, Petraglia MD. The Paleolithic in the Nihewan Basin, China: evolutionary history of an early to late Pleistocene record in Eastern Asia. Evol Anthrop. 2019; 29: 125-42.

45. Zhang SQ, Gao X, Zhang Y, Li ZY. Taphonomic analysis of the Lingjing fauna and the first report of a Middle Paleolithic kill-butchery site in North China. Chin Sci Bull. 2011 Oct; 56(30): 3213-19.

46. Chi W. Searching for descendants of "Pecking man". Anthropol Anz. 1979 Jun; 37(2): 61-7.
