# Supplementary material for: Palaeolithic polyhedrons, spheroids and bolas over time and space
Source: PLoS One. 2022 Jul 28;17(7):e0272135. doi: 10.1371/journal.pone.0272135 (PMC9333226; doi:10.1371/journal.pone.0272135)
Supplement: S4 Table — (PDF) [file pone.0272135.s004.pdf]

S4 Table. Types of environment and climate of the assemblages of the corpus.

Key of reading:

Names of assemblage in dark grey cells: assemblages for which we considered some objects as PSBs but most of authors that worked on the site did not (e.g., they could consider it as multifacial cores).

Light grey cells: the information in the cell is very probable but not certain, or is incomplete.

NA: no information collected.

Site	Water source nearby	Open environment	Mosaic environment	Wetland	Woodland	Tropical forest	Climate	Comment - Environment, climate
**Ewass Oldupa (Phase II)**	Yes	No	Yes	No	No	No	NA	Short woodland mosaics (Cueva-Temprana et al. 2022 [1]).
**Ewass Oldupa (Phase III)**	Yes	No	No	No	Yes	No	NA	"Mixed landscape of woodlands and palms" (Cueva-Temprana et al. 2022 [1]).
**Olduvai DK (Bed I)**	Yes	No	Yes	No	No	No	NA	Grasses, probably trees/shrubs (Albert et al. 2015 [2]).
**Olduvai FLK North (Bed I)**	Yes	NA	NA	NA	NA	NA	NA	NA
**Olduvai HWK-EE (Clay Unit, Bed II)**	Yes	Yes	Yes	No	No	No	NA	Open habitat with at least some trees (Pante & de la Torre 2018 [3]).
**Olduvai HWK-EE (SC Unit, Bed II)**	Yes	Yes	Yes	No	No	No	NA	Open habitat (Pante & de la Torre 2018 [3]).
**Olduvai SHK Main Site (Level A & B Bed II)**	Yes	Yes	Yes	No	No	No	NA	Open environment, with probably bushy and closed vegetation (Domínguez-Rodrigo et al. 2014 [4]).
**Olduvai BK (Level 1 to 3, Bed II)**	Yes	NA	NA	NA	NA	NA	NA	NA
**Olduvai BK (Level 4, Bed II)**	Yes	NA	NA	NA	NA	NA	NA	NA
**Olduvai TK (Bed II)**	Yes	Yes	Yes	No	No	No	Arid	Fauna typical of arid and open savannas from Bed II. Near the site, fluvial context and small woodland habitats (Gentry & Gentry 1978 [5], Yravedra et al. 2016 [6]).
**Melka Kunture (Karre Level K1-2)**	Yes	No	Yes	No	Yes	No	NA	"Montane grassland and forest/savanna ecotone" (Clark & Kurashina 1980 [7]).

**Melka Kunture (Garba IV)**	Yes	Yes	No	No	No	No	Arid	Savanna (Geraads 1985 [8]).
**Melka Kunture (Gombore IB)**	Yes	No	Yes	No	Yes	No	Humid	Humid savanna, cooler during Gombore 1B. Liana indicates a "light forest or gallery forest-edge environment" (Chavaillon 2004 [9]).
**Melka Kunture (Gombore II, Locality 1)**	NA	No	Yes	No	Yes	No	NA	"Montane grassland and forest/savanna ecotone" (Clark & Kurashina 1980 [7]).
**Melka Kunture (Gombore II, Locality 2)**	NA	No	Yes	No	Yes	No	NA	"Montane grassland and forest/savanna ecotone" (Clark & Kurashina 1980 [7]).
**Melka Kunture (Gombore II, Locality 3)**	NA	No	Yes	No	Yes	No	NA	"Montane grassland and forest/savanna ecotone" (Clark & Kurashina 1980 [7]).
**Melka Kunture (Gombore II, Locality 4)**	NA	No	Yes	No	Yes	No	NA	"Montane grassland and forest/savanna ecotone" (Clark & Kurashina 1980 [7]).
**Melka Kunture (Gombore II, Locality 5)**	NA	No	Yes	No	Yes	No	NA	"Montane grassland and forest/savanna ecotone" (Clark & Kurashina 1980 [7]).
**Melka Kunture (Gombore Iγ)**	NA	No	Yes	No	Yes	No	NA	"Montane grassland and forest/savanna ecotone" (Clark & Kurashina 1980 [7]).
**Melka Kunture (Simbiro III, level A)**	NA	No	Yes	No	Yes	No	NA	"Montane grassland and forest/savanna ecotone" (Clark & Kurashina 1980 [7]).
**Melka Kunture (Simbiro III, level B)**	NA	No	Yes	No	Yes	No	NA	"Montane grassland and forest/savanna ecotone" (Clark & Kurashina 1980 [7]).

**Melka Kunture (Simbiro III, level D)**	NA	No	Yes	No	Yes	No	NA	"Montane grassland and forest/savanna ecotone" (Clark & Kurashina 1980 [7]).
**Barogali**	NA	No	No	Yes	No	No	NA	Berthelet 2001 [10]
**Gadeb 2B**	Yes	No	Yes	No	Yes	No	NA	"Montane grassland and forest/savanna ecotone" (Clark & Kurashina 1980 [7]).
**Gadeb 2C**	Yes	No	Yes	No	Yes	No	NA	"Montane grassland and forest/savanna ecotone" (Clark & Kurashina 1980 [7]).
**Gadeb 2E**	Yes	No	Yes	No	Yes	No	NA	"Montane grassland and forest/savanna ecotone" (Clark & Kurashina 1980 [7]).
**Gadeb 8A**	Yes	No	Yes	No	Yes	No	NA	"Montane grassland and forest/savanna ecotone" (Clark & Kurashina 1980 [7]).
**Gadeb 8F**	Yes	No	Yes	No	Yes	No	NA	"Montane grassland and forest/savanna ecotone" (Clark & Kurashina 1980 [7]).
**Isenya (level V)**	Yes	No	Yes	No	No	No	Semi-arid	Wide plain of shrubby savanna, river bank environment (Clément 2019 [11]).
**Isenya (level VIa)**	Yes	No	Yes	No	No	No	Semi-arid	Wide plain of shrubby savanna, river bank environment (Clément 2019 [11]).
**Isenya (level VIb21)**	Yes	No	Yes	No	No	No	Semi-arid	Wide plain of shrubby savanna, river bank environment (Clément 2019 [11]).
**Olorgesailie Member 2**	Yes	No	Yes	No	No	No	NA	Mixed vegetation, C4 grasslands and C3 plants (Kinyanjui 2011 [12]).
**Olorgesailie Member 3**	Yes	No	Yes	No	No	No	NA	Mixed vegetation, C4 grasslands and C3 plants (Kinyanjui 2011 [12]).
**Olorgesailie Member 6**	Yes	No	No	Yes	No	No	NA	Potts et al. 1999 [13]
**Olorgesailie Member 7**	Yes	No	No	Yes	No	No	NA	Potts et al. 1999 [13]
**Olorgesailie Member 8**	Yes	NA	NA	NA	NA	No	NA	NA
**Olorgesailie Member 9**	Yes	NA	NA	NA	NA	No	NA	NA
**Olorgesailie Member 10**	Yes	NA	NA	NA	NA	No	NA	NA
**Olorgesailie Member 11**	Yes	NA	NA	NA	NA	No	NA	NA
**Isimila (K6)**	Yes	No	Yes	No	No	No	Semi-arid	Dry, drier than today; some vegetation (Howell et al. 1962 [14]).

**Isimila (LJ6-7)**	Yes	No	Yes	No	No	No	Semi-arid	Dry, drier than today (Howell et al. 1962 [14]).
**Isimila (K14)**	Yes	No	Yes	No	No	No	Semi-arid	Dry, drier than today (Howell et al. 1962 [14]).
**Isimila (H9-38)**	Yes	No	Yes	No	No	No	Semi-arid	Dry, drier than today (Howell et al. 1962 [14]).
**Ounjougou**	NA	NA	NA	NA	NA	NA	NA	NA
**Kabwe (Broken Hill)**	Yes	No	Yes	No	No	No	Semi-arid	Site close to an open grassland. It could be a savanna woodland (Avery 2003 [15]).
**Broken Hill (Sangoan)**	Yes	NA	NA	NA	NA	NA	NA	NA
**Broken Hill (Rhodesian Acheulian)**	Yes	NA	NA	NA	NA	NA	NA	NA
**Broken Hill (Hope Fountain Bariant)**	Yes	NA	NA	NA	NA	NA	NA	NA
**Kalambo Falls (A4 rubble IIA)**	NA	NA	NA	NA	NA	NA	NA	NA
**Kalambo Falls (A4 rubble IIB)**	NA	NA	NA	NA	NA	NA	NA	NA
**Kalambo Falls (A5 Comp)**	NA	No	No	No	Yes	No	NA	Clark 1964 [16]: "evergreen, riparian forest close to the river, of shallow, grass-filled, seasonally flooded valleys (dambos) and, on the higher slopes, *Brachystegia* woodland".
**Cave of Hearths (Bed 1-3)**	Yes	NA	NA	NA	NA	NA	NA	NA
**Swartkrans (SWT-M1, SPRP excavation)**	Yes	NA	NA	NA	NA	NA	NA	NA
**Swartkrans (SWT-M1, LB)**	Yes	No	Yes	No	No	No	Sub-tropical	Swartkrans: sub-tropical grassland and steppe (Clark & Kurashina 1980 [7]). In Kuman et al. 2018 [17]: "savanna woodland with riparian woodland and reed beds [...] edaphic grasslands supporting fresh grass grazers (Waston, 1993; Reed, 1997)."

**Swartkrans (SWT-M2)**	Yes	Yes	No	No	No	No	Sub-tropical	About Swartkrans in general: sub-tropical grassland and steppe (Clark & Kurashina 1980 [7]).
**Swartkrans (SWT-M3)**	Yes	Yes	No	No	No	No	Sub-tropical	About Swartkrans in general: sub-tropical grassland and steppe (Clark & Kurashina 1980 [7]).
**Vlakkraal Thermal springs**	Yes	NA	NA	NA	NA	NA	NA	NA
**Windhoek**	Yes	NA	NA	NA	NA	NA	NA	NA
**Esere**	Yes	NA	NA	NA	NA	NA	NA	NA
**Rhino Cave (Tsodilo Hills)**	NA	NA	NA	NA	NA	NA	NA	NA
**Corner Cave (Tsodilo Hills)**	NA	NA	NA	NA	NA	NA	NA	NA
**Kalkbank**	NA	Yes	Yes	No	No	No	Semi-arid	Grassland. Open woodland nearby. (Hutson & Cain 2008 [18]).
**Florisbad**	Yes	Yes	No	NA	No	No	Semi-arid	Kuman et al. 1999 [19]: "Periods of lowerspring discharge are represented by organic horizons as swampy vegetation [...]. During wet phases grasses were dominant, with a lush paleolake habitat surrounded by semi-desert or treeless grassland. [...] During the accumulation of the basal units, a treeless alpine grass-land implies that sedimentation began first in a cool, moist phase."
**Sterkfontein (Member 5)**	No	NA	NA	NA	NA	NA	NA	NA
**El Guettar**	Yes	NA	NA	NA	NA	NA	NA	NA
**Ain El Hallouf**	NA	NA	NA	NA	NA	NA	NA	NA
**Sidi Abderrahmane**	NA	NA	NA	NA	NA	NA	NA	NA
**Sidi Abderrahmane STIC**	NA	NA	NA	NA	NA	NA	NA	NA

**Erg Tihodaine (Coll. Arambourg)**	Yes	No	Yes	No	No	No	Semi-arid	Savanna type of fauna (Hocine 2016 [20]).
**Tighennif I**	NA	NA	NA	NA	NA	NA	NA	Various conclusions according to studies (synthesis in Djemmali 1985 : p.165-166 [21]).
**Ain Hanech**	Yes	No	Yes	No	No	No	NA	Fauna suggests an open savanna with watering place (i.e. Sahnouni 2006 [22]).
**Nzako Ambilo**	NA	No	No	No	No	Yes	Humid	Maybe a rainforest (Lupemban is possibly an adaptation to rainforest). Synthesis in Mesfin 2018 [23]).
**Nzako Kono**	NA	No	No	No	No	Yes	Humid	Maybe a rainforest (Lupemban is possibly an adaptation to rainforest). Synthesis in Mesfin 2018 [23]).
**M'Piaka**	NA	No	No	No	No	Yes	Humid	Maybe a rainforest (Lupemban is possibly an adaptation to rainforest). Synthesis in Mesfin 2018 [23]).
**Hummal (Levels 17 & 18)**	NA	Yes	No	No	No	No	Arid	Strictly steppic fauna (Le Tensorer et al. 2011 [24]).
**Shuwayhitiyah**	Yes	NA	NA	NA	NA	NA	NA	NA
**'Ubeidiya (III-20)**	Yes	NA	NA	NA	NA	NA	Humid	Belmaker 2006 [25]: "Described by Mallol (2004, 2006) as a "prolonged period of soil stability in a very wet environment". Diatom analysis suggests freshwater to oligosaline environment […] and shallow lake environments."

**'Ubeidiya (III-22)**	No	NA	NA	NA	NA	No	Semi-arid	Belmaker 2006 [25]: "Described by Mallol (2004, 2006) as "Drier conditions […]. No springs identified. […]"".
**Dursunlu**	Yes	Yes	No	Yes	No	No	Arid	Steppe and wetland (marsh or swamp). A part of the microfauna is typical from open steppe environments, so conditions were like today or perhaps cooler and drier. Some data about the paleoenvironment in Güleç et al. 2009 [26].
**North of Bridge Acheulian (NBA)**	Yes	NA	NA	NA	NA	NA	NA	NA
**Latamné**	Yes	Yes	No	No	No	No	NA	"Sub-tropical and steppe grassland" (Clark & Kurashina 1980 [7]).
**Joubb Jannine II**	NA	NA	NA	NA	NA	NA	NA	NA
**Khalliyé Sud**	NA	NA	NA	NA	NA	NA	NA	NA
**Wadi Fatimah**	NA	NA	NA	NA	NA	NA	NA	NA
**Revadim Quarry (Area D)**	NA	NA	NA	NA	NA	NA	NA	NA
**Evron Quarry**	Yes	No	Yes	No	Yes	No	NA	Bar-Yosef 1994 [27]: "The fauna indicate a mixture of woodland environment on the coastal plain."
**Evron East**	Yes	NA	NA	NA	NA	NA	NA	NA
**Saffaqah**	Yes	NA	NA	NA	NA	NA	NA	NA
**Qesem Cave**	Yes	No	Yes	No	Yes	No	Semi-arid	"Mosaic of open area with sparse vegetation but also shrubland, Mediterranean forest, rocky areas and riverbanks." Semi-arid (Maul et al. 2016 [28]).
**Bezez**	NA	NA	NA	NA	NA	NA	NA	NA
**Ma'ayan Barukh**	Yes	NA	NA	NA	NA	NA	NA	NA
**Kaletepe Deresi 3 (Level III)**	NA	NA	NA	NA	NA	NA	NA	NA
**Kaletepe Deresi 3 (Level III/IV)**	NA	NA	NA	NA	NA	NA	NA	NA

**Kaletepe Deresi 3 (Level IV)**	NA	NA	NA	NA	NA	NA	NA	NA
**Kaletepe Deresi 3 (Level V)**	NA	NA	NA	NA	NA	NA	NA	NA
**Kaletepe Deresi 3 (Level Vam)**	NA	NA	NA	NA	NA	NA	NA	NA
**Kaletepe Deresi 3 (Level V')**	NA	NA	NA	NA	NA	NA	NA	NA
**Kaletepe Deresi 3 (Level VI')**	NA	NA	NA	NA	NA	NA	NA	NA
**Kaletepe Deresi 3 (Level VII)**	NA	NA	NA	NA	NA	NA	NA	NA
**Kaletepe Deresi 3 (Level VIII)**	NA	NA	NA	NA	NA	NA	NA	NA
**Kaletepe Deresi 3 (Level IX)**	NA	NA	NA	NA	NA	NA	NA	NA
**Kaletepe Deresi 3 (Level X)**	NA	NA	NA	NA	NA	NA	NA	NA
**Kaletepe Deresi 3 (Level XI)**	NA	NA	NA	NA	NA	NA	NA	NA
**Kaletepe Deresi 3 (Level XII)**	NA	NA	NA	NA	NA	NA	NA	NA
**Santa Ana Cave**	NA	NA	NA	NA	NA	NA	NA	NA
**Barranco León**	Yes	Yes	Yes	No	No	No	NA	Synthesis in Titton 2020 [29] for the Iberian Peninsula: "dry stages were characterized by steppe, savannah and open woodland […] while the humid stages show an increase in the arboreal component with wooded steppe or open forests" (Titton 2020 [29]).
**Bois-de-Riquet (Unit 4)**	NA	NA	NA	NA	NA	NA	NA	NA
**Ca' Belvedere di Monte Poggiolo**	Yes	NA	NA	NA	NA	NA	NA	NA
**Dorn-Dürkheim 3**	Yes	Yes	No	No	No	No	Arid	Steppe with forest, lake-shore (Fiedler & Franzen 2002 [30]).
**La Noira (Stratum c)**	NA	NA	NA	NA	NA	No	Tem-perate	MIS 11 (Moncel et al. 2021 [31]) so probably temperate.
**Caune de l'Arago (Unit H1,2,3)**	Yes	NA	NA	NA	NA	No	Tem-perate, Humid	Temperate and humid (synthesis of Barsky 2013 [32]).
**Caune de l'Arago (Unit G)**	Yes	NA	NA	NA	NA	No	Cold, Dry	Fresh to cold and dry (synthesis of Barsky 2013 [32]).
**Caune de l'Arago (Unit E)**	Yes	Yes	No	No	No	No	Arid	Steppe. Cold and dry (synthesis of Barsky 2013 [32]).
**Caune de l'Arago (Unit D)**	Yes	NA	NA	NA	NA	No	Cold, Dry	More temperate than E levels. Cold and dry (synthesis of Barsky 2013 [32]).

**Treugol'Naya Cave (assemblage II)**	NA	Yes	No	No	Yes	No	Dry, Cool, Cold, Humid	Successively: cool and dry (subalpine meadow) / warm and humid (low altitude deciduous wood) / cool and dry (subalpine meadows) (Doronichev 2008 [33]).
**Duclos (0)**	NA	NA	NA	NA	NA	No	NA	NA
**Duclos (Ensemble IV)**	NA	NA	NA	NA	NA	No	NA	NA
**Duclos (Ensemble III)**	NA	NA	NA	NA	NA	No	NA	NA
**Septsos**	Yes	NA	NA	NA	NA	No	NA	NA
**Cerveny Kopec**	NA	NA	NA	NA	NA	No	NA	NA
**Bañugues (Asturias del Esferoid)**	NA	NA	NA	NA	NA	No	NA	NA
**Tourville-la-Rivière (level D2)**	NA	Yes	No	No	Yes	No	NA	Woodland and steppe (Cliquet 2010 [34]).
**Chez-Pinaud Jonzac (US 22)**	NA	Yes	No	No	No	No	Semi-arid	Cold, dry and open (abundant reindeer) (Claud et al. 2012 [35]).
**La Quina (level 8)**	NA	Yes	No	No	No	No	Cold	Open environment and cold climate according to fauna (Park 2007 [36]).
**Festons (Rebières valley)**	NA	NA	NA	NA	NA	No	NA	Abundant reindeer, so at least one very cold and long season (Pittard & de Saint-Périer 1955 [37])
**Sablière Rambour (Villers-Bocage)**	NA	NA	NA	NA	NA	No	NA	NA
**Isle-Adam (sablière de Cassan)**	NA	NA	NA	NA	NA	No	NA	NA
**Coll de la Guille (Terrasses du Roussillon)**	NA	NA	NA	NA	NA	No	NA	NA
**Mas Ferreol (Terrasses du Roussillon)**	NA	NA	NA	NA	NA	No	NA	NA
**Mas Ferrer (Terrasses du Roussillon)**	NA	NA	NA	NA	NA	No	NA	NA
**Le Puech de la Boule (Terrasses du Roussillon)**	NA	NA	NA	NA	NA	No	NA	NA
**Mas Bruno (Terrasses du Roussillon)**	NA	NA	NA	NA	NA	No	NA	NA
**Cabestany général (Terrasses du Roussillon)**	NA	NA	NA	NA	NA	No	NA	NA
**La Llabanère (Terrasses du Roussillon)**	NA	NA	NA	NA	NA	No	NA	NA
**Singi Talav (Layer 3)**	Yes	NA	NA	NA	NA	NA	NA	NA
**Singi Talav (Layer 4)**	Yes	NA	NA	NA	NA	NA	NA	NA

**Torajunga**	NA	NA	NA	NA	NA	NA	NA	NA
**Chirki Nevasa**	NA	NA	NA	NA	NA	NA	NA	NA
**Atit 2**	NA	NA	NA	NA	NA	NA	NA	NA
**Zhoukoudian 1 (Layer 1-3)**	NA	NA	NA	NA	NA	NA	NA	NA
**Zhoukoudian 1 (Layer 4-5)**	NA	NA	NA	NA	NA	NA	NA	NA
**Zhoukoudian 1 (QII)**	NA	NA	NA	NA	NA	NA	NA	NA
**Zhoukoudian 1 (Layer 8-9)**	NA	NA	NA	NA	NA	NA	NA	NA
**Liangshan Longgangsi**	NA	NA	NA	NA	NA	NA	NA	NA
**Dingcun**	Yes	NA	NA	NA	NA	NA	NA	NA
**Gongwangling**	NA	No	Yes	Yes	No	No	NA	"steppe with some forest, brush, and swamp" (Wang et al. 2014 [38]).
**Ganyu**	NA	NA	NA	NA	NA	NA	NA	NA
**Maling 2A**	NA	NA	NA	NA	NA	NA	NA	NA
**Shuigou-Huixinggou**	Yes	NA	NA	NA	NA	NA	NA	NA
**Zhoukoudian 15**	Yes	No	Yes	No	No	No	Tem-perate	Gao 2000 [39]: "warm-temperate and forest-steppe environment".
**Xujiayao**	Yes	No	Yes	No	No	No	Dry, cold	Forest-steppe vegetation. (Fuhua et al.1979 [40]). Dry and cold (Yang et al. 2019 [41]).
**Lingjing (Layer 11, lower part of layer 10)**	Yes	No	NA	NA	NA	No	NA	Li et al. 2019 [42], "warm and wet last interglacial period" (MIS5).
**Hsuchiyao**	Yes	No	No	No	Yes	No	Conti-nental	Chi 1979 [43]: "the climate around the lake was continental [...]. A microclimate might have developed as a result of the presence of the lake - warm and moist in summer, cool and rainy in spring and autumn, and cold in winter. [...] grassy lakeshore rose densely-forested slopes".
**Diaozhai**	NA	NA	NA	NA	NA	NA	NA	NA
**Jijiawan**	NA	NA	NA	NA	NA	NA	NA	NA
**Houjiapu**	NA	NA	NA	NA	NA	NA	NA	NA
**Zhoupo (Locality 95LP07)**	NA	NA	NA	NA	NA	NA	NA	NA

**Mansuri (Locality 1)**	NA	NA	NA	NA	NA	NA	NA	NA
**Jeongok-Ri (surface)**	NA	NA	NA	NA	NA	NA	NA	NA
**Jeongok-Ri (Layer 1)**	NA	NA	NA	NA	NA	NA	NA	NA
**Jeongok-Ri (Layer 2)**	NA	NA	NA	NA	NA	NA	NA	NA
**Jeongok-Ri (Layer 3)**	NA	NA	NA	NA	NA	NA	NA	NA
**Jangnamgyo (surface)**	NA	NA	NA	NA	NA	NA	NA	NA
**Jangnamgyo (Level 3)**	NA	NA	NA	NA	NA	NA	NA	NA
**Ngebung**	Yes	No	Yes	No	No	No	Arid	Dry, savanna or open woodland (Fauzi et al. 2016 [44]).
**Banjarejo**	Yes	NA	NA	NA	NA	NA	NA	NA
**Matar**	Yes	No	Yes	No	No	No	Arid	Typical dry and open-woodland or savanna (Fauzi et al. 2016 [44]).
**Solo**	NA	No	Yes	No	Yes	No	NA	Fauzi et al. 2016 [44]: "Dry, open woodland, development of deciduous forest. Appearance of browsing animals and arboreal primate."
**Baksoko River**	NA	No	Yes	No	No	No	Arid	Dry, open woodland (Fauzi et al. 2016 [44]).

References

1. Cueva-Temprana A, Lombao D, Soto M, Itambu M, Bushozi P, Boivin N, Petraglia M, Mercader J. Oldowan technology amid shifting environments ~2.03-1.83 million years ago. Front Ecol Evol. 2022; 10: 788101.
2. Albert RM, Bamford MK, Stanistreet I, Stollhofen H, Rivera-Rondón C, Rodríguez-Cintas A. Vegetation landscape at DK locality, Olduvai Gorge, Tanzania. Palaeogeogr Palaeoclimatol Palaeoecol. 2015; 426: 34-45.
3. Pante MC, de la Torre I. A hidden treasure of the Lower Pleistocene at Olduvai Gorge, Tanzania: the Leakey HWK EE assemblage. J Hum Evol. 2018 Jul; 120: 114-39.
4. Domínguez-Rodrigo M, Diez-Martín F, Yravedra J, Barba R, Mabulla A, Baquedano E et al. Study of the SHK main site faunal assemblage, Olduvai Gorge, Tanzania: implications for Bed II taphonomy, paleoecology, and hominin utilization of megafauna. Quat Int 2014; 322-323: 153-66 p.
5. Gentry AW, Gentry A. Fossil Bovidae (Mammalia) of Olduvai Gorge, Tanzania, part I. Bull Br Mus (Geology). 1978; 29: 289-446.
6. Yravedra J, Domínguez-Rodrigo M, Santonja M, Rubio-Jara S, Panera J, Pérez-González A et al. The larger mammal palimpsest from TK (Thiongo Korongo), Bed II, Olduvai Gorge, Tanzania. Quat Int. 2016; 417: 3-15.
7. Clark JD, Kurashina H. New Plio-Pleistocene archaeological occurences from the plain of Gadeb, Upper Webi Shebele basin, Ethiopia, and a statistical comparison of the Gadeb sites with other Early Stone Age assemblages. Anthropologie 1980; 18(2-3): 161-87.
8. Geraads D. La faune des gisements de Melka-Kunturé (Éthiopie). In: Ferembach D, editor. L'Environnement des Hominidés au Plio-Pléistocène. Paris: Masson; 1985. p. 165-74.
9. Chavaillon J. Prehistoric archaeology. The site of Gombore I. Discovery, geological introduction and study of percussion material and tools on pebble. In: Chavaillon J, Piperno M, editors. Studies on the Early Paleolithic site of Melka Kunture, Ethiopia. Florence: Istituto Italiano di Preistoria e Protostoria; 2004. p. 253-369.
10. Berthelet A. L'outillage lithique du site de dépeçage à *Elephas recki ileretensis* de Barogali (République de Djibouti). C R Acad Sci IIa. 2001 Mar; 332(6): 411-6.
11. Clément S. Les techniques de percussion : un reflet des changements techniques durant l'Acheuléen ? [doctoral thesis]. Nanterre, France: Université Paris X; 2019.
12. Kinyanjui RN. Phytolith analysis as a palaeoecological tool for reconstructing Mid-to Late-Pleistocene environments in the Olorgesailie Basin, Kenya [master thesis]. Cape Town, South Africa: University of Cape Town; 2011.
13. Potts R, Behrensmeyer AK, Ditchfield P. Paleolandscape variation and Early Pleistocene hominid activities: Members 1 and 7, Olorgesailie Formation, Kenya. J Hum Evol. 1999; 37: 747-88.
14. Howell FC, Cole GH, Kleindienst MR, Haldemann EG. Isimila: an Acheulian occupation site in the Iringa Highlands, Southern Highlands Province, Tanganyika. Musée Royal de l'Afrique Centrale 1962; 43-81.
15. Avery DM. Early and Middle Pleistocene environments and hominid biogeography; micromammalian evidence from Kabwe, Twin Rivers and Mumbwa Caves in central Zambia. Palaeogeogr Palaeoclimatol Palaeoecol. 2003; 189: 55-69.
16. Clark JD. Prehistory in southern Africa. In: Ki-Zerbo J, editor. General history of Africa, vol. 1: methodology and African Prehistory. London: Heinemann; 1981. p. 487-529.
17. Kuman K, Sutton MB, Pickering TR, Heaton JL. The Oldowan industry from Swartkrans cave, South Africa, and its relevance for the African Oldowan. J Hum Evol. 2018; 123: 52-69.
18. Hutson JM, Cain CR. Reanalysis and reinterpretation of the Kalkbank faunal accumulation, Limpopo Province, South

Africa. Journal of Taphonomy 2008; 6(3-4): 399-428.

19. Kuman K, Inbar M, Clarke RJ. Paleoenvironments and cultural sequence of the Florisbad Middle Stone Age hominid site, South Africa. J Archaeol Sci. 1999; 26: 1409-25.

20. Hocine S. Le site acheuléen d'Erg Tihodaïne : caractéristiques technologiques de l'industrie lithique du Pléistocène moyen (Sahara central, Algérie). Anthropologie. 2016 Jun; 120(3): 263-84.

21. Djemmali NE. L'industrie lithique acheuléenne du gisement de Tighennif (Ternifine), Algérie [doctoral thesis]. Paris, France: Muséum National d'Histoire Naturelle, Université Pierre et Marie Curie; 1985.

22. Sahnouni M. Les plus vieilles traces d'occupation humaine en Afrique du Nord : perspectives de l'Ain Hanech, Algérie. C R Palevol. 2006; 5: 243-54.

23. Mesfin I. Les assemblages lithiques Lupembiens conservés au Muséum National d'Histoire naturelle : apports et perspectives pour la connaissance du *Middle Stone Age* d'Afrique centrale [master thesis]. Paris, France: Muséum National d'Histoire Naturelle; 2018.

24. Le Tensorer JM, Von Falkenstein V, Le Tensorer H, Schmid P, Muhesen S. Etude préliminaire des industries archaïques de faciès Oldowayen du site de Hummal (El Kowm, Syrie centrale). Anthropologie. 2011; 115: 247-66.

25. Belmaker M. Community structure through time: 'Ubeidiya, a Lower Pleistocene site as a case study [doctoral thesis]. Jerusalem, Israel: Hebrew University; 2006.

26. Güleç E, White T, Kuhn S, Özer I, Sagir M, Yilmaz H et al. The Lower Pleistocene lithic assemblage from Dursunlu (Konya), central Anatolia, Turkey. Antiquity 2009 Mar; 83(319): 11-22.

27. Bar-Yosef O. The lower Paleolithic of the Near East. J World Prehist. 1994 Sep; 8(3): 211-65.

28. Maul LC, Bruch AA, Smith KT, Shenbrot G, Barkai R, Gopher A. Palaeoecological and biostratigraphical implications of the microvertebrates of Qesem Cave in Israel. Quat Int. 2016; 398: 219-32.

29. Titton S. Lithic assemblage, percussive technologies and behaviour at the Oldowan site of Barranco León (Orce, Andalucía, Spain) [doctoral thesis]. Tarragona, Spain: Universitat Rovira I Virgili; 2020.

30. Fiedler L, Franzen JL. Artefakte vom altpleistozänen Fundplatz "Dorn-Dürkheim 3" am nördlichen Oberrhein. Germania: Anzeiger der Römisch-Germanischen Kommission des Deutschen Archäologischen Instituts 2002; 80(2): 421-40.

31. Moncel MH, García-Medrano P, Despriée J, Arnaud J, Voinchet P, Bahain JJ. Tracking behavioral persistence and innovations during the Middle Pleistocene in Western Europe. Shift in occupations between 700 and 450 ka at la Noira site (Centre, France). J Hum Evol. 2021; 156: 103009.

32. Barsky D. The Caune de l'Arago stone industries in their stratigraphical context. C R Palevol. 2013; 12: 305-25.

33. Doronichev VB. The Lower Paleolithic in Eastern Europe and the Caucasus: a reappraisal of the data and new approaches. PaleoAnthropology 2008: 107-57.

34. Cliquet D. Tourville-la-Rivière, Seine-Maritime : carrières et ballastières de Normandie : la Fosse-Marmitaine. Inrap Grand-Ouest; 2010. 105 p.

35. Claud É, Soressi M, Jaubert J, Hublin JJ. Étude tracéologique de l'outillage moustérien de type Quina du bonebed de Chez-Pinaud à Jonzac (Charente-Maritime). Nouveaux éléments en faveur d'un site de boucherie et de traitement des peaux. Gallia Préhistoire 2012; 54(2): 3-32.

36. Park SJ. Systèmes de production lithique et circulation des matières premières au Paléolithique moyen récent et final. Une approche techno-économique à partir de l'étude des industries lithiques de La Quina (Charente) [doctoral thesis]. Nanterre, France: Université Paris X; 2007.

37. Pittard E, de Saint-Périer RS. Les Festons, gisement paléolithique à Brantôme (Dordogne). Arch Suisses Anthropol Gen. 1955; 20(1-2): 1-141.

38. Wang S, Lu H, Xing L. Chronological and typo-technological perspectives on the Palaeolithic archaeology in Lantian, central China. Quat Int. 2014; 347: 183-92.

39. Gao X. Explanations of typological variability in Paleolithic remains from Zhoukoudian locality 15, China [doctoral thesis]. Tucson, United States of America: University of Arizona; 2000.

40. Fuhua Y, Yongying Y, Xueshun M, Yuexia L. On the environment and geological age of Xujiayao site from pollen analysis data. Seismology and Ecology 1979; 1(4): 72-8.

41. Yang SX, Deng CL, Zhu RX, Petraglia MD. The Paleolithic in the Nihewan Basin, China: evolutionary history of an early to late Pleistocene record in Eastern Asia. Evol Anthrop. 2019; 29: 125-42.

42. Li H, Li ZY, Gao X, Kuman K, Summer A. Technological behaviour of the early Late Pleistocene archaic humans at Lingjing (Xuchang, China). Archaeol Anthropol Sci. 2019; 11(7): 3477-90.

43. Chi W. Searching for descendants of "Pecking man". Anthropol Anz. 1979; 37(2): 61-7.

44. Fauzi MR, Ansyori MM, Prastiningtyas D, Intan MFS, Wibowo UP, Wulandari et al. Matar: a forgotten but promising Pleistocene locality in East Java. Quat Int. 2016; 416: 183-92.
