# Supplementary material for: Palaeolithic polyhedrons, spheroids and bolas over time and space
Source: PLoS One. 2022 Jul 28;17(7):e0272135. doi: 10.1371/journal.pone.0272135 (PMC9333226; doi:10.1371/journal.pone.0272135)
Supplement: S5 Table — (PDF) [file pone.0272135.s005.pdf]

S5 Table. Fauna recorded on the assemblages of the corpus.

Key of reading:

Names of assemblage in dark grey cells: assemblages for which we considered some objects as PSBs but most of authors that worked on the site did not (e.g., they could consider it as multifacial cores).

Light grey cells: the information in the cell is very probable but not certain, or is incomplete.

Abbreviations: P= polyhedron; S= spheroid; SS= subspheroid; B= bola.

NA: no information collected.

Site	*Cervidae*	*Equidae*	*Bovidae*	*Proboscidea*	*Suidae*	Other big mammals	Comment - Fauna	Anthropically broken bones	Comment - Broken bones
**Ewass Oldupa (Phase II)**	NA	NA	NA	NA	NA	NA	NA	NA	NA
**Ewass Oldupa (Phase III)**	Yes	No	Yes	No	No	No	Cueva-Temprana et al. 2022 [1]	NA	NA
**Olduvai DK (Bed I)**	NA	NA	NA	NA	NA	NA	NA	NA	NA
**Olduvai FLK North (Bed I)**	NA	NA	NA	NA	NA	NA	NA	NA	NA
**Olduvai HWK-EE (Clay Unit, Bed II)**	No	Yes	Yes	No	Yes	Yes	Dominated by bovids (Pante & de la Torre 2018 [2]).	Yes	Majority of limb bones intentionally broken for access to marrow (Pante & de la Torre 2018 [2]).
**Olduvai HWK-EE (SC Unit, Bed II)**	No	Yes	Yes	Yes	Yes	Yes	Dominated by bovids (Pante & de la Torre 2018 [2]).	Yes	Majority of limb bones intentionally broken for access to marrow (Pante & de la Torre 2018 [2]).
**Olduvai SHK Main Site (Level A & B Bed II)**	No	Yes	Yes	Yes	Yes	Yes	Exploitation of large and medium-sized animals in SHK, more limited than in BK (Domínguez-Rodrigo et al. 2014 [3]) and TK where more lithic activities have been attested.	No	Probably biotic agents for bones breakage. 219 long bone shaft fragments analysed: only 4 with notches (Domínguez-Rodrigo et al. 2014 [3]).
**Olduvai BK (Level 1 to 3, Bed II)**	No	Yes	Yes	NA	Yes	Yes	Domínguez-Rodrigo et al. 2009 [4]	Yes	Maybe marrow exploitation, but more focus on large bone meat than on marrow (Domínguez-Rodrigo et al. 2009 [4]).
**Olduvai BK (Level 4, Bed II)**	No	Yes	Yes	NA	No	NA	Domínguez-Rodrigo et al. 2009 [4]	Yes	Large amount of cut marks and percussion traces (Domínguez-Rodrigo et al. 2009 [4]).

**Olduvai TK (Bed II)**	No	Yes	Yes	No	Yes	Yes	Mostly bovids (47.8%), equids (45.7%), then hippos and suids. (Egeland 2007 [5]). Details: Yravedra et al. 2016 [6].	No	No notch on bones. Percussion marks and tooth marks limited. High bone fragmentation would be taphonomic (Yravedra et al. 2016 [6]).
**Melka Kunture (Karre Level K1-2)**	No	Yes	Yes	No	No	Yes	85 faunal remains (for 62, the species is unidentified). Bovids, hippo, equids (Berthelet & Chavaillon 2004 [7]).	NA	NA
**Melka Kunture (Garba IV)**	No	Yes	Yes	No	Yes	Yes	Mostly antelopes. Details: Geraads 1985 [8], Chavaillon & Piperno 1975 [9].	NA	NA
**Melka Kunture (Gombore IB)**	No	Yes	Yes	Yes	Yes	Yes	Hippo and antelopes. Suids badly preserved. Equids and elephants are rare (Chavaillon 2004 [10]).	NA	NA
**Melka Kunture (Gombore II, Locality 1)**	NA	NA	NA	NA	NA	NA	NA	NA	NA
**Melka Kunture (Gombore II, Locality 2)**	No	Yes	No	No	No	Yes	2 hippos, equids (Chavaillon & Berthelet 2004 [11]).	Yes	Chavaillon & Berthelet 2004 [11]
**Melka Kunture (Gombore II, Locality 3)**	NA	NA	NA	NA	NA	NA	NA	NA	NA
**Melka Kunture (Gombore II, Locality 4)**	NA	NA	NA	NA	NA	NA	NA	NA	NA
**Melka Kunture (Gombore II, Locality 5)**	NA	NA	NA	NA	NA	NA	NA	NA	NA
**Melka Kunture (Gombore Iγ)**	No	Yes	Yes	No	Yes	Yes	Mussi et al. Forthcoming [12]	NA	NA
**Melka Kunture (Simbiro III, level A)**	NA	NA	Yes	NA	NA	NA	Abundance of animal bones and teeth (Chavaillon & Berthelet 2004 [11]).	NA	NA
**Melka Kunture (Simbiro III, level B)**	No	Yes	Yes	No	No	Yes	Chavaillon & Berthelet 2004 [11]	NA	NA

**Melka Kunture (Simbiro III, level D)**	No	Yes	Yes	No	No	Yes	Well preserved faunal remains (22% of the assemblage) (Chavaillon & Berthelet 2004 [11]).	NA	NA
**Barogali**	No	No	No	Yes	No	No	Only one elephant, scavenged (Berthelet 2001 [13]).	Yes	Extraction of marrow (or detachment of tendons) (Berthelet 2001 [13]).
**Gadeb 2B**	NA	NA	NA	NA	NA	NA	NA	NA	NA
**Gadeb 2C**	NA	NA	NA	NA	NA	NA	NA	NA	NA
**Gadeb 2E**	No	No	Yes	Yes	Yes	Yes	Clark & Kurashina 1979 [14]	NA	NA
**Gadeb 8A**	NA	NA	NA	NA	NA	NA	NA	NA	Impossible to interpret the juxtaposition of faunal remains and lithics (de la Torre 2011 [15]).
**Gadeb 8F**	No	No	No	No	No	Yes	Butchery of a hippo (Clark & Kurashina 1979 [14]; Clark 1987 [16]).	Yes	Assefa et al. 1982 [17]: scapula, long bone and rib fragments may have been broken to access the marrow.
**Isenya (level V)**	No	Yes	Yes	Yes	Yes	Yes	Roche et al. 1988 [18]	Yes	Roche et al. 1988 [18]: Diaphyses and epiphyses of long bones are broken. The first cause of the fragmentation may be anthropical (marrow extraction), but other factors could also be involved (e.g., sedimentation, carnivores).
**Isenya (level VIa)**	No	Yes	Yes	Yes	Yes	Yes	Roche et al. 1988 [18]	Yes	Roche et al. 1988 [18]: Diaphyses and epiphyses of long bones are broken. The first cause of the fragmentation may be anthropical (marrow extraction), but other factors could also be involved (e.g., sedimentation, carnivores).

**Isenya (level VIb21)**	No	Yes	Yes	Yes	Yes	Yes	Roche et al. 1988 [18]	Yes	Roche et al. 1988 [18]: Diaphyses and epiphyses of long bones are broken. The first cause of the fragmentation may be anthropical (marrow extraction), but other factors could also be involved (e.g., sedimentation, carnivores).
**Olorgesailie Member 2**	NA	NA	NA	NA	NA	NA	NA	NA	NA
**Olorgesailie Member 3**	NA	NA	NA	NA	NA	NA	NA	NA	NA
**Olorgesailie Member 6**	NA	Yes	Yes	NA	NA	Yes	Clément 2019 [19]).	NA	NA
**Olorgesailie Member 7**	NA	Yes	Yes	NA	NA	Yes	Clément 2019 [19]).	NA	NA
**Olorgesailie Membre 8**	NA	NA	NA	NA	NA	NA	NA	NA	NA
**Olorgesailie Member 9**	NA	NA	NA	NA	NA	NA	NA	NA	NA
**Olorgesailie Member 10**	NA	NA	NA	NA	NA	NA	NA	NA	NA
**Olorgesailie Member 11**	NA	NA	NA	NA	NA	NA	NA	NA	NA
**Isimila (K6)**	NA	NA	NA	NA	NA	NA	NA	NA	NA
**Isimila (LJ6-7)**	NA	NA	NA	NA	NA	NA	NA	NA	NA
**Isimila (K14)**	NA	NA	NA	NA	NA	NA	NA	NA	NA
**Isimila (H9-38)**	NA	NA	NA	NA	NA	NA	NA	NA	NA
**Ounjougou**	No	No	No	No	No	No	Soil too acid to preserve bones (de Weyer 2017	No	NA
**Kabwe (Broken Hill)**	No	No	Yes	No	Yes	Yes	Klein 1973 [21]	NA	NA
**Broken Hill (Sangoan)**	NA	NA	NA	NA	NA	NA	NA	NA	NA
**Broken Hill (Rhodesian Acheulian)**	NA	NA	NA	NA	NA	NA	NA	NA	NA
**Broken Hill (Hope Fountain Bariant)**	NA	NA	NA	NA	NA	NA	NA	NA	NA
**Kalambo Falls (A4 rubble IIA)**	No	No	No	No	No	No	No fauna preserved (Clark 1981 [illegible]	No	No fauna preserved (Clark 1981 [22]).
**Kalambo Falls (A4 rubble IIB)**	No	No	No	No	No	No	No fauna preserved (Clark 1981 [22]).	No	No fauna preserved (Clark 1981 [22]).
**Kalambo Falls (A5 Comp)**	No	No	No	No	No	No	No fauna preserved (Clark 1981 [22]).	No	No fauna preserved (Clark 1981 [22]).

**Cave of Hearths (Bed 1-3)**	NA	NA	NA	NA	NA	NA	NA	NA	NA
**Swartkrans (SWT-M1, SPRP excavation)**	NA	NA	NA	NA	NA	NA	NA	NA	NA
**Swartkrans (SWT-M1, LB)**	No	Yes	Yes	Yes	Yes	Yes	Fauna from M2 and M3 approximately the same than M1 (Brain et al. 1988 [23], Clark 1993 [24]).	NA	NA
**Swartkrans (SWT-M2)**	No	Yes	Yes	Yes	Yes	Yes	Fauna from M2 and M3 approximately the same than M1 (Brain et al. 1988 [23], Clark 1993 [24]).	NA	NA
**Swartkrans (SWT-M3)**	No	Yes	Yes	Yes	Yes	Yes	Fauna from M2 and M3 approximately the same than M1 (Brain et al. 1988 [23], Clark 1993 [24]).	NA	NA
**Vlakkraal Thermal springs**	No	Yes	Yes	No	Yes	Yes	Data from Wells et al. 1942 [25].	NA	NA
**Windhoek**	NA	NA	NA	NA	NA	NA	NA	NA	NA
**Esere**	NA	NA	NA	NA	NA	NA	NA	NA	NA
**Rhino Cave (Tsodilo Hills)**	NA	NA	NA	NA	NA	NA	NA	NA	NA
**Corner Cave (Tsodilo Hills)**	NA	NA	NA	NA	NA	NA	NA	NA	NA
**Kalkbank**	No	Yes	Yes	Yes	Yes	Yes	Hutson & Cain 2008 [26]	Yes	Involvement of animals and taphonomy in the bone fragmentation, but for a small part it may also involve humans (Huston & Cain 2008 [26]).
**Florisbad**	No	No	Yes	No	No	Yes	Brink 1987 [27], Kuman et al. 1999 [28]	NA	NA
**Sterkfontein (Member 5)**	No	Yes	No	No	Yes	Yes	Kuman & Clarke 2000 [29]	NA	NA
**El Guettar**	No	Yes	Yes	No	No	Yes	Gruet 1950 [30]	NA	NA
**Ain El Hallouf**	NA	NA	NA	NA	NA	NA	NA	NA	NA
**Sidi Abderrahmane**	NA	NA	NA	NA	NA	NA	NA	NA	NA
**Sidi Abderrahmane STIC**	NA	NA	NA	NA	NA	NA	NA	NA	NA

**Erg Tihodaine (Coll. Arambourg)**	No	Yes	Yes	Yes	Yes	Yes	Thomas 1977 [31]	NA	NA
**Tighennif I**	No	Yes	Yes	Yes	Yes	Yes	Djemmali 1985 [32]	Yes	Some broken long bones, percussion marks, probably to extract marrow (Denys et al. 1984 [33]).
**Ain Hanech**	No	Yes	Yes	Yes	Yes	Yes	Synthesis in Sahnouni et al. 2013 [34].	NA	NA
**Nzako Ambilo**	NA	NA	NA	NA	NA	NA	NA	NA	NA
**Nzako Kono**	NA	NA	NA	NA	NA	NA	NA	NA	NA
**M'Piaka**	NA	NA	NA	NA	NA	NA	NA	NA	NA
**Hummal (Levels 17 & 18)**	No	Yes	Yes	No	No	Yes	Dominance of *Camelidae* (Le Tensorer et al. 2011 [35]).	NA	NA
**Shuwayhitiyah**	NA	NA	NA	NA	NA	NA	NA	NA	NA
**'Ubeidiya (III-20)**	Yes	Yes	Yes	No	Yes	Yes	Belmaker 2006 [36]	No	No percussion marks on bone fragments, no extraction of marrow (Belmaker 2006 [36]).
**'Ubeidiya (III-22)**	Yes	Yes	Yes	Yes	Yes	Yes	Belmaker 2006 [36]	No	Cut-marks, but according to Belmaker 2006 [36] not related to crushing bones to access the marrow.
**Dursunlu**	Yes	Yes	Yes	Yes	No	Yes	Güleç et al. 2009 [37]	No	Güleç et al. 2009 [37]: possibly cut-marks on a metatarsus of a large bird.
**North of Bridge Acheulian (NBA)**	Yes	Yes	Yes	Yes	No	No	Sharon et al. 2010 [38]	NA	NA
**Latamné**	Yes	Yes	Yes	Yes	No	Yes	Bar-Yosef 1994 [39]	NA	NA
**Joubb Jannine II**	No	Yes	No	No	No	No	Only one equid teeth recovered (Yazbeck 2002 [40]).	No	Only 1 equid teeth (Yazbeck 2002 [40]).
**Khalliyé Sud**	NA	NA	NA	NA	NA	NA	NA	NA	NA
**Wadi Fatimah**	No	No	No	No	No	No	No fauna. Details about the site: Petraglia 2003 [41].	No	NA
**Revadim Quarry (Area D)**	NA	NA	NA	NA	NA	NA	NA	NA	NA
**Evron Quarry**	Yes	No	Yes	Yes	Yes	Yes	Tchernov et al. 1994 [42]	NA	NA
**Evron East**	NA	NA	NA	NA	NA	NA	Photo of tooth of a large mammal (Shemer & Barzilai 2017 [43]).	NA	NA
**Saffaqah**	No	No	No	No	No	No	No fauna. Details about the site: Petraglia 2003 [41].	No	NA
**Qesem Cave**	Yes	Yes	Yes	No	Yes	Yes	Maul et al. 2016 [44]	Yes	Assaf et al. 2020 [45]: PSBs could have been used to break bones and access the marrow.
**Bezez**	NA	NA	NA	NA	NA	NA	NA	NA	NA

**Ma'ayan Barukh**	No	No	No	Yes	No	No	Only fragments of tusk and of an elephant molar (Stekelis & Gilead 1966 [46]).	No	NA
**Kaletepe Deresi 3 (Level III)**	NA	NA	NA	NA	NA	NA	Slimak 2008 [47]: Only few remains of fauna (high acidity of the soil). "However, we cannot rule out the possibility that the scarcity of bone is related to the kinds of activities that took place at KD3 prehistorically."	NA	NA
**Kaletepe Deresi 3 (Level III/IV)**	NA	NA	NA	NA	NA	NA	Slimak 2008 [47]: Only few remains of fauna (high acidity of the soil). "However, we cannot rule out the possibility that the scarcity of bone is related to the kinds of activities that took place at KD3 prehistorically."	NA	NA
**Kaletepe Deresi 3 (Level IV)**	NA	NA	NA	NA	NA	NA	Slimak 2008 [47]: Only few remains of fauna (high acidity of the soil). "However, we cannot rule out the possibility that the scarcity of bone is related to the kinds of activities that took place at KD3 prehistorically."	NA	NA
**Kaletepe Deresi 3 (Level V)**	NA	NA	NA	NA	NA	NA	Slimak 2008 [47]: Only few remains of fauna (high acidity of the soil). "However, we cannot rule out the possibility that the scarcity of bone is related to the kinds of activities that took place at KD3 prehistorically."	NA	NA
**Kaletepe Deresi 3 (Level Vam)**	NA	NA	NA	NA	NA	NA	Slimak 2008 [47]: Only few remains of fauna (high acidity of the soil). "However, we cannot rule out the possibility that the scarcity of bone is related to the kinds of activities that took place at KD3 prehistorically."	NA	NA
**Kaletepe Deresi 3 (Level V')**	NA	NA	NA	NA	NA	NA	Slimak 2008 [47]: Only few remains of fauna (high acidity of the soil). "However, we cannot rule out the possibility that the scarcity of bone is related to the kinds of activities that took place at KD3 prehistorically."	NA	NA

**Kaletepe Deresi 3 (Level VI')**	NA	NA	NA	NA	NA	NA	Slimak 2008 [47]: Only few remains of fauna (high acidity of the soil). “However, we cannot rule out the possibility that the scarcity of bone is related to the kinds of activities that took place at KD3 prehistorically.”	NA	NA
**Kaletepe Deresi 3 (Level VII)**	NA	NA	NA	NA	NA	NA	Slimak 2008 [47]: Only few remains of fauna (high acidity of the soil). “However, we cannot rule out the possibility that the scarcity of bone is related to the kinds of activities that took place at KD3 prehistorically.”	NA	NA
**Kaletepe Deresi 3 (Level VIII)**	NA	NA	NA	NA	NA	NA	Slimak 2008 [47]: Only few remains of fauna (high acidity of the soil). “However, we cannot rule out the possibility that the scarcity of bone is related to the kinds of activities that took place at KD3 prehistorically.”	NA	NA
**Kaletepe Deresi 3 (Level IX)**	NA	NA	NA	NA	NA	NA	Slimak 2008 [47]: Only few remains of fauna (high acidity of the soil). “However, we cannot rule out the possibility that the scarcity of bone is related to the kinds of activities that took place at KD3 prehistorically.”	NA	NA
**Kaletepe Deresi 3 (Level X)**	NA	NA	NA	NA	NA	NA	Slimak 2008 [47]: Only few remains of fauna (high acidity of the soil). “However, we cannot rule out the possibility that the scarcity of bone is related to the kinds of activities that took place at KD3 prehistorically.”	NA	NA

**Kaletepe Deresi 3 (Level XI)**	NA	NA	NA	NA	NA	NA	Slimak 2008 [47]: Only few remains of fauna in the sequence, probably because of the high acidity of the soil. “However, we cannot rule out the possibility that the scarcity of bone is related to the kinds of activities that took place at KD3 prehistorically.”	NA	NA
**Kaletepe Deresi 3 (Level XII)**	NA	NA	NA	NA	NA	NA	Slimak 2008 [47]: Only few remains of fauna in the sequence, probably because of the high acidity of the soil. “However, we cannot rule out the possibility that the scarcity of bone is related to the kinds of activities that took place at KD3 prehistorically.”	NA	NA
**Santa Ana Cave**	Yes	Yes	NA	NA	NA	Yes	García-Vadillo et al. 2022 [48]	No	García-Vadillo et al. 2022 [48]: scarce anthropic modifications on bones.
**Barranco León**	Yes	Yes	Yes	Yes	No	Yes	Rodríguez- Gomez et al. 2016 [49]	Yes	Bones fragments with cutmarks (Titton 2020 [50]).
**Bois-de-Riquet (Unit 4)**	NA	NA	NA	NA	NA	NA	NA	NA	NA
**Ca’ Belvedere di Monte Poggiolo**	NA	NA	Yes	Yes	NA	Yes	Details in: Terradillos-Bernal & Moncel 2004 [51]).	NA	NA
**Dorn-Dürkheim 3**	Yes	Yes	Yes	Yes	No	Yes	Fiedler & Franzen 2002 [52]	NA	Fiedler et al. 2019 [53]: no direct evidence of animal butchery (weathering), but some clues of hominin involvement in the accumulation (e.g., anatomic selection of skeletal elements).

**La Noira (Stratum c)**	No	No	No	No	No	No	No fauna discovered in the stratum. Data about the site: Moncel et al. 2021 [54]	NA	NA
**Caune de l'Arago (Unit H1,2,3)**	Yes	Yes	Yes	No	No	Yes	Occupants were cervid hunters (Barsky 2001 [55]). Data: de Lumley 2021 [56].	NA	NA
**Caune de l'Arago (Unit G)**	Yes	Yes	Yes	Yes	Yes	Yes	Occupants were big herbivore hunters (Barsky 2001 [55]). Data: de Lumley 2021 [56].	NA	NA
**Caune de l'Arago (Unit E)**	Yes	Yes	Yes	Yes	No	Yes	Occupants were mouflon hunters (Barsky 2001 [55]). Data: de Lumley 2021 [56].	NA	NA
**Caune de l'Arago (Unit D)**	Yes	Yes	Yes	No	No	Yes	Seasonal habitat of hunters (Barsky 2001 [55]). Data: de Lumley 2021 [56].	Yes	Long bone broken for marrow extraction (Barsky et al. 2019 [57]).
**Treugol'Naya Cave (assemblage II)**	Yes	No	Yes	No	No	Yes	Doronichev 2008 [58]	No	Probably a carnivore accumulation. Highly fragmented (bone trampling) (Doronichev et al. 2007 [59]).
**Duclos (0)**	No	No	No	No	No	No	No fauna discovered, probably because of taphonomical process (Colonge 2012 [60]).	No	No fauna discovered (probably taphonomical process) but butchery activities probably took place on the site (Colonge 2012 [60]).
**Duclos (Ensemble IV)**	No	No	No	No	No	No	No fauna discovered, probably because of taphonomical process (Colonge 2012 [60]).	No	No fauna discovered (probably taphonomical process) but butchery activities probably took place on the site (Colonge 2012 [60]).

**Duclos (Ensemble III)**	No	No	No	No	No	No	No fauna discovered, probably because of taphonomic al process (Colonge 2012 [60]).	No	No fauna discovered (probably taphonomical process) but butchery activities probably took place on the site (Colonge 2012 [60]).
**Septsos**	NA	NA	NA	NA	NA	NA	NA	NA	NA
**Cerveny Kopec**	NA	NA	NA	NA	NA	NA	NA	NA	NA
**Bañugues (Asturias del Esferoid)**	NA	NA	NA	NA	NA	NA	NA	NA	NA
**Tourville-la-Rivière (level D2)**	Yes	Yes	Yes	No	No	Yes	Cliquet 2010 [61]	Yes	Bones intentionally broken to extract the marrow. Cliquet 2010 [61].
**Jonzac (Chez Pinaud, US 22)**	Yes	Yes	Yes	No	No	Yes	Niven et al. 2012 [62]	Yes	Long bones intentionally broken to extract the marrow. Cliquet 2010 [61].(Niven et al. 2012 [62]).
**La Quina (level 8)**	Yes	NA	NA	NA	NA	NA	Reindeer is dominant (Park 2007 [63]).	Yes	Reindeer intentionally broken to extract marrow from long bones (Park 2007 [63]).
**Festons (Rebières valley)**	Yes	Yes	Yes	Yes	Yes	Yes	Pittard & de Saint-Périer 1955 [64]	Yes	Abundant bones that may have been broken to access marrow: (Pittard & de Saint-Périer 1955 [64]).
**Sablière Rambour (Villers-Bocage)**	NA	NA	NA	NA	NA	NA	NA	NA	NA
**Isle-Adam (sablière de Cassan)**	NA	NA	NA	NA	NA	NA	NA	NA	NA

**Coll de la Guille (Terrasses du Roussillon)**	NA	NA	NA	NA	NA	NA	NA	NA	NA
**Mas Ferreol (Terrasses du Roussillon)**	NA	NA	NA	NA	NA	NA	NA	NA	NA
**Mas Ferrer (Terrasses du Roussillon)**	NA	NA	NA	NA	NA	NA	NA	NA	NA
**Le Puech de la Boule (Terrasses du Roussillon)**	NA	NA	NA	NA	NA	NA	NA	NA	NA
**Mas Bruno (Terrasses du Roussillon)**	NA	NA	NA	NA	NA	NA	NA	NA	NA
**Cabestany général (Terrasses du Roussillon)**	NA	NA	NA	NA	NA	NA	NA	NA	NA
**La Llabanère (Terrasses du Roussillon)**	NA	NA	NA	NA	NA	NA	NA	NA	NA
**Singi Talav (Layer 3)**	No	No	No	No	No	No	Bones not preserved. Data about the site: e.g. Gaillard & Rajaguru 2017 [65].	No	Bones not preserved. Data about the site: e.g. Gaillard & Rajaguru 2017 [65].
**Singi Talav (Layer 4)**	No	No	No	No	No	No	Bones not preserved. Data about the site: e.g. Gaillard & Rajaguru 2017 [65].	No	Bones not preserved. Data about the site: e.g. Gaillard & Rajaguru 2017 [65].
**Torajunga**	NA	NA	NA	NA	NA	NA	NA	NA	NA
**Chirki Nevasa**	No	No	Yes	No	No	No	2 fragments of teeth of *bos* (Corvinus 1983 [66]).	NA	NA
**Atit 2**	NA	NA	NA	NA	NA	NA	NA	NA	NA
**Zhoukoudian 1 (Layer 1-3)**	NA	NA	NA	NA	NA	NA	NA	NA	NA
**Zhoukoudian 1 (Layer 4-5)**	NA	NA	NA	NA	NA	NA	NA	NA	NA
**Zhoukoudian 1 (QII)**	NA	NA	NA	NA	NA	NA	NA	NA	NA
**Zhoukoudian 1 (Layer 8-9)**	NA	NA	NA	NA	NA	NA	NA	NA	NA
**Liangshan Longgangsi**	NA	NA	NA	NA	NA	NA	NA	NA	NA
**Dingcun**	NA	NA	NA	NA	NA	NA	28 types of mammals in the entire site (13 assemblages) (Yang et al. 2014 [67]).	NA	NA
**Gongwangling**	Yes	Yes	NA	Yes	Yes	Yes	All the fauna may not come from layers with PSBs. Data from Wang et al. 2014 [68].	NA	NA
**Ganyu**	NA	NA	NA	NA	NA	NA	NA	NA	NA

**Maling 2A**	No	No	No	No	No	No	No fossil found at the site. For data about the assemblage: Pei et al. 2015 [69].	NA	NA
**Shuigou-Huixinggou**	No	Yes	No	No	No	No	One equid premolar (Li et al. 2017 [70]).	NA	NA
**Zhoukoudian 15**	Yes	Yes	Yes	Yes	NA	Yes	Gao 2000 [71]	NA	NA
**Xujiayao**	Yes	Yes	Yes	Yes	Yes	Yes	Norton & Gao 2008 [72]	Yes	Norton & Gao 2008 [72], "Xujiayao hominins had primary access to high utility (meat-bearing and marrow-rich) long bones".
**Lingjing (Layer 11, lower part of layer 10)**	Yes	Yes	Yes	Yes	Yes	Yes	Zhang et al. 2011 [73]	Yes	Bones may have been broken to access marrow (Zhang et al. 2011 [73]).
**Hsuchiyao**	Yes	Yes	Yes	Yes	No	Yes	Chi 1979 [74]	Yes	Chi 1979 [74]: "there was not a single complete animal skeleton or skull. Most of them were fragments and they were probably cracked for sucking out the marrow or broken to fashion into tools."
**Diaozhai**	NA	NA	NA	NA	NA	NA	NA	NA	NA
**Jijiawan**	NA	NA	NA	NA	NA	NA	NA	NA	NA
**Houjiapu**	NA	NA	NA	NA	NA	NA	NA	NA	NA
**Zhoupo (Locality 95LP07)**	NA	NA	NA	NA	NA	NA	NA	NA	NA
**Mansuri (Locality 1)**	NA	NA	NA	NA	NA	NA	NA	NA	NA
**Jeongok-Ri (surface)**	NA	NA	NA	NA	NA	NA	NA	NA	NA
**Jeongok-Ri (Layer 1)**	NA	NA	NA	NA	NA	NA	NA	NA	NA
**Jeongok-Ri (Layer 2)**	NA	NA	NA	NA	NA	NA	NA	NA	NA
**Jeongok-Ri (Layer 3)**	NA	NA	NA	NA	NA	NA	NA	NA	NA
**Jangnamgyo (surface)**	NA	NA	NA	NA	NA	NA	NA	NA	NA
**Jangnamgyo (Level 3)**	NA	NA	NA	NA	NA	NA	NA	NA	NA
**Ngebung**	Yes	No	Yes	Yes	No	No	Bouteaux & Moigne 2010 [75]	NA	NA
**Banjarejo**	NA	NA	NA	NA	NA	NA	NA	NA	NA
**Matar**	Yes	No	Yes	Yes	Yes	Yes	Fauzi et al. 2016 [76]	NA	NA
**Solo**	NA	NA	NA	NA	NA	NA	NA	NA	NA
**Baksoko River**	NA	NA	NA	NA	NA	NA	NA	NA	NA

References

1. Cueva-Temprana A, Lombao D, Soto M, Itambu M, Bushozi P, Boivin N, Petraglia M, Mercader J. Oldowan technology amid shifting environments ~2.03-1.83 million years ago. Front Ecol Evol. 2022; 10: 788101.
2. Pante MC, de la Torre I. A hidden treasure of the Lower Pleistocene at Olduvai Gorge, Tanzania: the Leakey HWK EE assemblage. J Hum Evol. 2018 Jul; 120: 114-39.
3. Domínguez-Rodrigo M, Diez-Martín F, Yravedra J, Barba R, Mabulla A, Baquedano E et al. Study of the SHK main site faunal assemblage, Olduvai Gorge, Tanzania: implications for Bed II taphonomy, paleoecology, and hominin utilization of megafauna. Quat Int. 2014; 322-323: 153-66.
4. Domínguez-Rodrigo M, Mabulla A, Bunn HT, Barba R, Diez-Martín F, Egeland CP et al. Unraveling hominin behavior at another anthropogenic site from Olduvai Gorge (Tanzania): new archaeological and taphonomic research at BK, Upper Bed II. J Hum Evol. 2009; 57: 260-83.
5. Egeland CP. Zooarchaeological and taphonomic perspectives on hominid and carnivore interactions at Olduvai Gorge, Tanzania [doctoral thesis]. Bloomington, United States of America: University of Indiana; 2007.
6. Yravedra J, Domínguez-Rodrigo M, Santonja M, Rubio-Jara S, Panera J, Pérez-González A et al. The larger mammal palimpsest from TK (Thiongo Korongo), Bed II, Olduvai Gorge, Tanzania. Quat Int. 2016; 417: 3-15.
7. Berthelet A, Chavaillon J. Prehistoric archaeology. The site of Karre I: Karre I. In: Chavaillon J, Piperno M, editors. Studies on the Early Paleolithic site of Melka Kunture, Ethiopia. Florence: Origines; 2004. p. 211-51.
8. Geraads D. La faune des gisements de Melka-Kunturé (Éthiopie). In: Ferembach D, editor. L'Environnement des Hominidés au Plio-Pléistocène. Paris: Masson; 1985. p. 165-74.
9. Chavaillon J, Piperno M. Garba IV, site paléolithique ancien de Melka-Kunturé. Bulletin de la Société préhistorique française. 1975; 72: 134-8.
10. Chavaillon J. Prehistoric archaeology. The site of Gombore I: discovery, geological introduction and study of percussion material and tools on pebble. In: Chavaillon J, Piperno M, editors. Studies on the Early Paleolithic site of Melka Kunture, Ethiopia. Florence: Istituto Italiano di Preistoria e Protostoria; 2004. p. 253-369.
11. Chavaillon J, Berthelet A. The archaeological sites of Melka Kunture. In: Chavaillon J, Piperno M, editors. Studies on the Early Paleolithic site of Melka Kunture, Ethiopia. Florence: Istituto Italiano di Preistoria e Protostoria; 2004. p. 25-80.
12. Mussi M, Altamura F, Di Bianco L, Bonnefille R, Gaudzinski-Windheuser S, Geraads D et al. After the emergence of the Acheulean at Melka Kunture (Upper Awash, Ethiopia): from Gombore IB (1.6 Ma) to Gombore Iγ (1.4 Ma), Gombore 1σ (1.3 Ma) and Gombore II OAM Test Pit C (1.2 Ma). Quat Int. Forthcoming.
13. Berthelet A. L'outillage lithique du site de dépeçage à *Elephas recki ileretensis* de Barogali (République de Djibouti). C R Acad Sci IIa. 2001 Mar; 332(6): 411-6.
14. Clark JD, Kurashina H. Hominid occupation of the East-Central Highlands of Ethiopia in the Plio-Pleistocene. Nature 1979 Nov; 282: 33-9.
15. De la Torre I. The Early Stone Age lithic assemblages of Gadeb (Ethiopia) and the Developed Oldowan/early Acheulean in East Africa. J Hum Evol. 2011; 60: 768-812.
16. Clark JD. Transitions: *Homo erectus* and the Acheulian: the Ethiopian sites of Gadeb and the middle Awash. J Hum Evol. 1987; 16(7-8): 809-26.
17. Assefa G, Clark JD, Williams MAJ. Late Cenozoic history and archaeology of the Upper Webi Shebele basin, East Central Ethiopia. SINET: Ethiopian J Sci. 1982; 5(1): 27-46.

18. Roche H, Brugal JP, Lefevre D, Ploux S, Texier PJ. Isenya: état des recherches sur un nouveau site acheuléen d'Afrique orientale. Afr Archaeol Rev. 1988; 6(1): 27-55.

19. Clément S. Les techniques de percussion : un reflet des changements techniques durant l'Acheuléen ? [doctoral thesis]. Nanterre, France: Université Paris X; 2019.

20. De Weyer L. An Early Stone Age in Western Africa? Spheroids and polyhedrons at Ounjougou, Mali. Journal of Lithic Studies 2017; 4(1).

21. Klein RG. Geological antiquity of Rhodesian Man. Nature 1973 Aug; 244: 311-2

22. Clark JD. Prehistory in southern Africa. In: Ki-Zerbo J, editor. General history of Africa, vol. 1: methodology and African Prehistory. London: Heinemann; 1981. p. 487-529.

23. Brain CK, Churcher CS, Clark JD, Grine FE, Shipman P, Susman RL et al. New evidence of early hominids, their culture and environment from the Swartkrans cave, South Africa. S Afr J Sci. 1988 Oct; 84: 828-835.

24. Clark JD. Stone artefact assemblages from Members 1-3, Swartkrans Cave. In: Brain C, editor. Swartkrans: a cave's chronicle of early man. Transvaal Museum Monograph No. 8. Pretoria: Transvaal Museum; 1993. 167-94.

25. Wells LH, Cooke HBS, Malan BD, Wells LH, Cooke HBS. The associated fauna and culture of the Vlakkraal Thermal Springs, O.F.S. Transactions of the Royal Society of South Africa 1942; 29(3): 203-33.

26. Hutson JM, Cain CR. Reanalysis and reinterpretation of the Kalkbank faunal accumulation, Limpopo Province, South Africa. Journal of Taphonomy 2008; 6(3-4): 399-428.

27. Brink JS. The archaeozoology of Florisbad, Orange Free State [doctoral thesis]. Stellenbosch, South Africa: Stellenbosch University; 1987.

28. Kuman K, Inbar M, Clarke RJ. Paleoenvironments and cultural sequence of the Florisbad Middle Stone Age hominid site, South Africa. J Archaeol Sci. 1999; 26: 1409-25.

29. Kuman K, Clarke RJ. Stratigraphy, artefact industries and hominid associations for Sterkfontein, Member 5. J Hum Evol. 2000; 38: 827-47.

30. Gruet M. Note préliminaire sur le gisement moustérien d'El Guettar. Bulletin de la Société préhistorique de France 1950; 47(5): 232-41.

31. Thomas H. Géologie et Paléontologie du gisement acheuléen de l'Erg Tihodaïne (Ahaggar, Sahara central). Mém Cent rech anthropol préhist ethnogr. (Alger). 1977; 27.

32. Djemmali NE. L'industrie lithique acheuléenne du gisement de Tighennif (Ternifine), Algérie [doctoral thesis]. Paris, France: Muséum National d'Histoire Naturelle, Université Pierre et Marie Curie; 1985.

33. Denys C, Patou M, Djemmali N. Tighennif (Ternifine, Algérie). Premiers résultats concernant l'origine de l'accumulation du matériel osseux de ce gisement Pléistocène. C R Acad Sc. 1984; 299(8): 481-6.

34. Sahnouni M, Rosell J, Van der Made J, María Vergès J, Ollé A, Kandi N et al. The first evidence of cut marks and usewear traces from the Plio-Pleistocene locality of El-Kherba (Ain Hanech), Algeria: implications for early hominin subsistence activities circa 1.8 Ma. J Hum Evol. 2013; 64: 137-50.

35. Le Tensorer JM, Von Falkenstein V, Le Tensorer H, Schmid P, Muhesen S. Etude préliminaire des industries archaïques de faciès Oldowayen du site de Hummal (El Kowm, Syrie centrale). Anthropologie. 2011; 115: 247-66.

36. Belmaker M. Community structure through time: 'Ubeidiya, a Lower Pleistocene site as a case study [doctoral thesis]. Jerusalem, Israel: Hebrew University; 2006.

37. Güleç E, White T, Kuhn S, Özer I, Sagir M, Yilmaz H et al. The Lower Pleistocene lithic assemblage from Dursunlu (Konya), central Anatolia, Turkey. Antiquity 2009 Mar; 83(319): 11-22.

38. Sharon G, Feibel C, Alperson-Afil N, Harlavan Y, Feraud G, Ashkenazi S et al. New evidence for the Northern Dead Sea rift Acheulian. PaleoAnthropology Society 2010: 79-99.

39. Bar-Yosef O. The lower Paleolithic of the Near East. J World Prehist. 1994 Sep; 8(3): 211-65.

40. Yazbeck C. Les systèmes techniques de production au Paléolithique inférieur en Beqaa Libanaise : le cas de Joubb Jannine II [doctoral thesis]. Lyon, France: Université Lumière Lyon 2; 2002.

41. Petraglia MD. The Lower Paleolithic of the Arabian Peninsula: occupations, adaptations, and dispersals. J World Prehist. 2003 Jun; 17(2): 141-79.

42. Tchernov E, Horwitz LK, Ronen A, Lister A. The faunal remains from Evron Quarry in relation to other Lower Paleolithic hominid sites in the Southern Levant. Quat Res. 1994; 42: 328-39.

43. Shemer M, Barzilai O. 'Evron (East): preliminary report. Israel Antiquities Authority; 2017. 6p.

44. Maul LC, Bruch AA, Smith KT, Shenbrot G, Barkai R, Gopher A. Palaeoecological and biostratigraphical implications of the microvertebrates of Qesem Cave in Israel. Quat Int. 2016; 398: 219-32.

45. Assaf E, Caricola I, Gopher A, Rosell J, Blasco R, Bar O, et al. Shaped stone balls were used for bone marrow extraction at Lower Paleolithic Qesem Cave, Israel. PLoS One. 2020; 15(4): e0230972.

46. Stekelis M, Gilead D. Ma'ayan Barukh: a Lower Palaeolithic site in Upper Galilee. Mitekufat Haeven: Journal of the Israel Prehistoric Society 1966; 1-23.

47. Slimak L, Kuhn SL, Roche H, Mouralis D, Buitenhuis H, Balkan-Atli N et al. Kaletepe Deresi 3 (Turkey): archaeological evidence for early human settlement in Central Anatolia. J Hum Evol. 2008; 54: 99-111.

48. García-Vadillo FJ, Canals-Salomó A, Rodríguez-Alvarez XP, Carbonell-Roura E. The large flake Acheulian with spheroids from Santa Ana Cave (Cáceres, Spain). J Archaeol Sci Rep. 2022; 41: 103265.

49. Rodríguez-Gómez G, Palmqvist P, Rodríguez J, Mateos A, Martín-González JA, Espigares MP et al. On the ecological context of the earliest human settlements in Europe: resource availability and competition intensity in the carnivore guild of Barranco León-D and Fuente Nueva-3 (Orce, Baza Basin, SE Spain). Quat Sci Rev. 2016; 143: 69-83.

50. Titton S. Lithic assemblage, percussive technologies and behaviour at the Oldowan site of Barranco León (Orce, Andalucía, Spain) [doctoral thesis]. Tarragona, Spain: Universitat Rovira I Virgili; 2020.

51. Terradillos Bernal M, Moncel MH. Contribution à l'étude de la technologie du Paléolithique « archaïque » du sud de l'Europe selon le Système Logique Analytique (SLA). Application aux sites du Vallonnet (Roquebrune-Cap-Martin, France), de Gran Dolina TD6 (Burgos, Espagne), de Ca'Belvedere de Monte Poggiolo (Forli, Italie) et de Barranco Léon et Fuente Nueva 3 (Orce, Espagne). Anthropologie. 2004; 108: 307-29.

52. Fiedler L, Franzen JL. Artefakte vom altpleistozänen Fundplatz "Dorn-Dürkheim 3" am nördlichen Oberrhein. Germania: Anzeiger der Römisch-Germanischen Kommission des Deutschen Archäologischen Instituts 2002; 80(2): 421-40.

53. Fiedler L, Humburg C, Klingelhöfer H, Stoll S, Stoll M. Several Lower Palaeolithic sites along the Rhine Rift Valley, dated from 1.3 to 0.6 million years. Humanities 2019; 8(129).

54. Moncel MH, García-Medrano P, Despriée J, Arnaud J, Voinchet P, Bahain JJ. Tracking behavioral persistence and innovations during the Middle Pleistocene in Western Europe. Shift in occupations between 700 and 450 ka at la Noira site (Centre, France). J Hum Evol. 2021; 156: 103009.

55. Barsky D. Le débitage des industries lithiques de la Caune de l'Arago (Pyrénées-Orientales, France) : leur place dans l'évolution des industries du Paléolithique inférieur en Europe méditerranéenne [doctoral thesis]. Perpignan, France: Université de Perpignan; 2001.

56. De Lumley H, Batalla i Llasat G, Fontaneil C, Grégoire S, Pollet G. Caune de l'Arago, Tautavel-en-Roussillon Pyrénées-Orientales,

France : les industries paléolithiques du Pléistocène moyen et du début du Pléistocène supérieur, fascicule I (généralités). Paris: CNRS Editions; 2021. 378 p.

57. Barsky D, Moigne AM, Pois V. The shift from typical Western European Late Acheulian to microproduction in unit 'D' of the late Middle Pleistocene deposits of the Caune de l'Arago (Pyrénées-Orientales, France). J Hum Evol. 2019; 135: 102650.

58. Doronichev VB. The Lower Paleolithic in Eastern Europe and the Caucasus: a reappraisal of the data and new approaches. PaleoAnthropology 2008: 107-57.

59. Doronichev VB, Golovanova LV, Baryshnikov GF, Blackwell BAB, Garutt NV, Levkovskaya GM et al. Treugol'Naya Cave: the Early Palaeolithic in Caucasus and Eastern Europe. St Petersburg; 2007.

60. Colonge D. Aquitaine, A65, Pyrénées-Atlantiques, Auriac, Duclos : Pléistocène moyen et Antiquité en Béarn. Inrap Grand Sud-Ouest; 2012. 476 p.

61. Cliquet D. Tourville-la-Rivière, Seine-Maritime: carrières et ballastières de Normandie : la Fosse-Marmitaine. Inrap Grand-Ouest; 2010. 105 p.

62. Niven L, Steele TE, Rendu W, Mallye JB, McPherron SP, Soressi M et al. Neandertal mobility and large-game hunting: the exploitation of reindeer during the Quina Mousterian at Chez-Pinaud Jonzac (Charente-Maritime, France). J Hum Evol. 2012; 63: 624-35.

63. Park SJ. Systèmes de production lithique et circulation des matières premières au Paléolithique moyen récent et final. Une approche techno-économique à partir de l'étude des industries lithiques de La Quina (Charente) [doctoral thesis]. Nanterre, France: Université Paris X; 2007.

64. Pittard E, de Saint-Périer RS. Les Festons, gisement paléolithique à Brantôme (Dordogne). Arch Suisses Anthropol Gen. 1955; 20(1-2): 1-141.

65. Gaillard C, Rajaguru SN. Revisiting the Acheulian site of Singi Talav at Didwana (Rajasthan) 35 years later. In: Deo SG, Baptista A, Joglekar J, editors. Rethinking the past: a tribute to Professor V.N. Misra. Pune: Indian Society for Prehistoric and Quaternary Studies; 2017. p. 25-39.

66. Corvinus G. A survey of the Pravara River system in western Maharashtra, India, vol. 2: the excavation of the Acheulian site of Chirki-on-Pravara, India. Tübingen: Archaeologica Vanatoria; 1983. 466 p.

67. Yang SX, Huang WW, Hou YM, Yuan BY. Is the Dingcun lithic assembly a "chopper-chopping tool industry", or "Late Acheulian"? Quat Int. 2014; 321: 3-11.

68. Wang S, Lu H, Xing L. Chronological and typo-technological perspectives on the Palaeolithic archaeology in Lantian, central China. Quat Int. 2014; 347: 183-92.

69. Pei S, Niu D, Guan Y, Nian X, Yi M, Ma N et al. Middle Pleistocene hominin occupation in the Danjiangkou Reservoir region, central China: studies of formation processes and stone technology of Maling 2A site. J Archaeol Sci. 2015; 53: 391-407.

70. Li X, Ao H, Dekkers MJ, Roberts AP, Zhang P, Lin S et al. Early Pleistocene occurrence of Acheulian technology in North China. Quat Sci Rev. 2017; 156: 12-22.

71. Gao X. Explanations of typological variability in Paleolithic remains from Zhoukoudian locality 15, China [doctoral thesis]. Tucson, United States of America: University of Arizona; 2000.

72. Norton CJ, Gao X. Hominin–carnivore interactions during the Chinese Early Paleolithic: taphonomic perspectives from Xujiayao. J Hum Evol. 2008 Jul; 55(1): 164-78.

73. Zhang SQ, Gao X, Zhang Y, Li ZY. Taphonomic analysis of the Lingjing fauna and the first report of a Middle Paleolithic kill-butchery site in North China. Chin Sci Bull. 2011 Oct; 56(30): 3213-19.

74. Chi W. Searching for descendants of "Pecking man". Anthropol Anz. 1979; 37(2): 61-7.

75. Bouteaux A, Moigne AM. New taphonomical approaches : the Javanese Pleistocene open-air sites (Sangiran, central Java). Quat Int. 2010; 223-224: 220-5.

76. Fauzi MR, Ansyori MM, Prastiningtyas D, Intan MFS, Wibowo UP, Wulandari et al. Matar: a forgotten but promising Pleistocene locality in East Java. Quat Int. 2016; 416: 183-92.
