# Supplementary material for: Palaeolithic polyhedrons, spheroids and bolas over time and space
Source: PLoS One. 2022 Jul 28;17(7):e0272135. doi: 10.1371/journal.pone.0272135 (PMC9333226; doi:10.1371/journal.pone.0272135)
Supplement: S6 Table — (PDF) [file pone.0272135.s006.pdf]

S6 Table. Lithic items in the assemblages of the corpus.

Key of reading:

Names of assemblage in dark grey cells: assemblages for which we considered some objects as PSBs but most of authors that worked on the site did not (e.g., they could consider it as multifacial cores).

Light grey cells: the information in the cell is very probable but not certain, or is incomplete. **When only PSBs are mentioned in a cell, no data were recorded about the existing lithic assemblage associated**.

Raw materials in bold: main raw material in terms of quantity of items.

Types of debitage are not exhaustive.

Abbreviations: Prop= proportions; P= polyhedron; S= spheroid; SS= subspheroid; B= bola; Ba= basalt; C= chert; D= diabase; F= flint; G= granite; L= limestone; Q= quartz; Qzite= quartzite; Ph= phonolite; Sa= sandstone; LCT= large cutting tools; LDT= light duty tools; HDT= heavy duty tools

NA: no information collected.

Percentages of HDTs and LDTs have been calculated from data available in the literature and may be approximations.

Site	Lithic assemblage	Comment - Items	HDT - Raw materials	HDT - Prop. (in %)	LDT - Raw materials	LDT - Prop. (in %)	Types of debitage	Comment - Lithic assemblages
**Ewass Oldupa (Phase II)**	Spheroid, Chopper or Chopping tool, Core, Flake, Fragment, Debris, Hammerstone, Notch, Denticulate, Side scraper	201 artefacts (Cueva-Temprana et al. 2022) [1].	Qzite, Ignimbrite	4.98	Qzite	74.13	Bipolar on anvil	All debitage methods: multipolar multifacial, unipolar longitudinal, bifacial orthogonal, bipolar on anvil, bifacial centripetal. Percentages calculated with the data from Cueva-Temprana et al. 2022 [1].
**Ewass Oldupa (Phase III)**	Spheroid, Core, Flake, Fragment, Debris, Notch, Hammerstone	188 artefacts (Cueva-Temprana et al. 2022) [1].	Qzite	1.06	**Qzite**, Ignimbrite	87.23	Unipolar, Bipolar	All debitage methods: multipolar multifacial, unipolar longitudinal, bifacial orthogonal, bifacial centripetal, unifacial centripetal, unifacial bipolar. Percentages calculated with the data from Cueva-Temprana et al. 2022 [1].
**Olduvai DK (Bed I)**	Polyhedron	NA	NA	NA	NA	NA	NA	Sahnouni 1993 [2]
**Olduvai FLK North (Bed I)**	Polyhedron, Spheroid	NA	NA	NA	NA	NA	NA	Sahnouni 1993 [2], a photo of S and P in Mora & de la Torre 2005 [3]
**Olduvai HWK - EE (Clay Unit, Bed II)**	Core, Chopper, Discoid, Polyhedron, Proto biface, Denticulate, Side scraper, Anvil, Hammerstone, Spheroid, Flake, Debris	457 objects (Pante & de la Torre 2018 [4]).	**Qzite**, Ph, Ba, C, Trachyte, Gneiss	16.7	**Qzite**, Ph, Ba, C, Trachyte, Gneiss	31.3	Bipolar, Discoid, Unipolar	Percentages calculated with the data from Pante & de la Torre 2018 [4].
**Olduvai HWK-EE (SC Unit, Bed II)**	Core, Chopper, Discoid, Polyhedron, Proto biface, Denticulate, Side scraper, Anvil, Hammerstone, Spheroid, Flake, Debris	3030 objects (Pante & de la Torre 2018 [4]).	**Qzite**, Ph, Ba, C, Trachyte, Gneiss	15.8	**Qzite**, Ph, Ba, C, Trachyte, Gneiss	16	Bipolar, Discoid, Unipolar	Percentages calculated with the data from Pante & de la Torre 2018 [4].
**Olduvai SHK Main Site (Level A & B Bed II)**	Core, Hammerstone, Handaxe, Chopper core, Polyhedron, Spheroid, Denticulate, Scraper, Awl, Anvil, Flake, Debris	1773 objects (Sánchez-Yustos et al. 2019 [5]). Only 2 LCT (handaxes) .	**Qzite**, Ba, Ph	6.19	**Qzite**, Ba, Ph	63.17	Unipolar, Bipolar, Discoid, Oppor-tunistic	Percentages calculated with the data from Sánchez- Yustos et al. 2019 [5]. Multifacial cores (considered here as P) are the main reduction scheme (35.5%).
**Olduvai BK (Level 1 to 3, Bed II)**	Core, Polyhedron, Manuport, Hammerstone, Flake, Debris	1151 artifacts (Diez- Martin et al. 2009 [6]).	NA	NA	NA	NA	Bipolar, Discoid, Levallois	Diez-Martin et al. 2009 [6].
**Olduvai BK (Level 4, Bed II)**	Core, Manuport, Hammerstone, Spheroid, Flake, Debris	424 artifacts (Diez-Martin et al. [illegible]	NA	NA	NA	NA	Bipolar, Discoid, Levallois	Diez-Martin et al. 2009 [6].

**Olduvai TK (Bed II)**	Core, Chopper, Biface, Trihedral pick, Cleaver, Polyhedron, Spheroid, Core scraper, Scraper, Burin, Awl, Notch, Bec, Knife, Anvil, Flake, Debris	5805 artifacts (Santonja et al. 2014 [7]).	**Qzite**, Volcanic rock	2.27	**Qzite**, Volcanic rock	90	Unipolar, Bipolar, Opportunistic, Discoid	Percentages calculated with the data from Santonja et al. 2014 [7] data. Less hammerstones in TKLF than in TKUF.
**Melka Kunture (Karre Level K1-2)**	Core, Hammerstone, Chopper, Polyhedron, Heavy endscraper, Notch pebble, Rabot, Notch, Denticulate pebble, Handaxe, Debris, Flake, Side scraper, Endscraper, Awl, Notch, Denticulate	178 objects (16 from K1, 162 from K2) (Berthelet & Chavaillon 2004 [8]).	**Ba, Trachyte**, Obsidian	28.01	Ba, Obsidian, Trachyte, Tuff	33.71	Unipolar, Bipolar	Percentages calculated with the data from Berthelet & Chavaillon 2004 [8].
**Melka Kunture (Garba IV)**	Core, Endscraper, Side scraper, Burin, Perforator, Notch, Denticulate, Borer, Chopper, Polyhedron, Rabot, Heavy endscraper, Spheroid, Handaxe, Cleaver, Flake, Debris	9821 artifacts (Gallotti 2013 [9]). 2 cleavers.	Ba, Lava, Trachyte, Tuff	11.81	Obsidian, Lava	55.15	Bipolar, Discoid	Percentages calculated with the data from Gallotti 2013 [9].
**Melka Kunture (Gombore IB)**	Core, Hammerstone, Chopper, Polyhedron, Rabot, Spheroid, Heavy endscraper, Handaxe, Flake, Debris	10411 artifacts. Also pebble tools (Chavaillon 2004 [10], Mussi et al. Forthcoming [11]).	**Ba**, Obsidian	18	**Obsidian**, Ba	20.84	Unipolar	Percentages calculated with the data from Chavaillon 2004 [10].
**Melka Kunture (Gombore II, Locality 1)**	Core, Chopper, Polyhedron, Bola, Rabot, Heavy endscraper, Handaxe, Cleaver, Side scraper, Endscraper, Awl, Notch, Denticulate, Anvil, Flake	666 artifacts, no debris collected (Chavaillon & Berthelet 2004 [12]). Only 1 cleaver.	Ba, Obsidian	15.47	**Obsidian**, Ba	44.59	Unipolar	Percentages calculated with the data from Chavaillon & Berthelet 2004 [12].
**Melka Kunture (Gombore II, Locality 2)**	Core, Chopper, Polyhedron, Bola, Heavy endscraper, Rabot, Notch on pebble, Handaxe, Anvil, Flake, Debris	81 artifacts, only 2 debris collected (Chavaillon & Berthelet 2004 [12]).	Volcanic rock	54.32	Volcanic rock	23.46	NA	Percentages calculated with the data from Chavaillon & Berthelet 2004 [12].

**Melka Kunture (Gombore II, Locality 3)**	Core, Chopper, Polyhedron, Handaxe, Cleaver, Side scraper, Endscraper, Awl, Anvil, Flake	742 artifacts, no debris collected (Chavaillon & Berthelet 2004 [12]).	Ba, Obsidian	16.31	**Obsidian**, Ba	64.69	Unipolar	Percentages calculated with the data from Chavaillon & Berthelet 2004 [12].
**Melka Kunture (Gombore II, Locality 4)**	Core, Chopper, Polyhedron, Handaxe, Rabot, Heavy endscraper, Pick, Side scraper, Endscraper, Awl, Knife, Notch, Denticulate, Anvil, Flake, Debris	134 artifacts, only 2 debris collected. Also pebble tools (Chavaillon & Berthelet 2004 [12]). 1 pick.	Ba, Obsidian	19.4	**Obsidian**, Ba	63.43	Unipolar	Percentages calculated with the data from Chavaillon & Berthelet 2004 [12].
**Melka Kunture (Gombore II, Locality 5)**	Core, Chopper, Polyhedron, Handaxe, Rabot, Cleaver, Heavy endscraper, Pick, Side scraper, Endscraper, Notch, Denticulate, Awl, Knife, Anvil, Flake	211 artifacts, no debris collected. Also pebble tools (Chavaillon & Berthelet 2004 [12]). Only 1 pick, 1 cleaver.	Ba, Obsidian	25.12	**Obsidian**, Ba	57.82	Unipolar	Percentages calculated with the data from Chavaillon & Berthelet 2004 [12].
**Melka Kunture (Gombore Iγ)**	Core, Chopper, Polyhedron, Heavy endscraper, Pick, Side scraper, Endscraper, Awl, Knife, Notch, Denticulate, Anvil, Flake	1266 artifacts (Chavaillon & Berthelet 2004 [12]).	Volcanic rock	11.06	Volcanic rock	28.28	Unipolar	Percentages calculated with the data from Chavaillon & Berthelet 2004 [12]. 2 operative chains: production of small-medium sized flakes vs. production of large flakes (some used as core blanks) (Mussi et al. 2021 [11].
**Melka Kunture (Simbiro III, level A)**	Core, Chopper, Polyhedron, Heavy endscraper, Handaxe, Side scraper, Endscraper, Awl, Perforator, Notch, Denticulate, Anvil, Flake	246 artifacts. No debris collected (Chavaillon & Berthelet 2004 [12]).	**Ba, Tuff**, Obsidian	8.13	**Obsidian**, Volcanic Rock	69.51	NA	Percentages calculated with the data from Chavaillon & Berthelet 2004 [12].
**Melka Kunture (Simbiro III, level B)**	Core, Chopper, Polyhedron, Heavy endscraper, Handaxe, Cleaver, Side scraper, Endscraper, Awl, Perforator, Notch, Denticulate, Anvil, Flake	769 artifacts. No debris collected (Chavaillon & Berthelet 2004 [12]).	Volcanic rock	27.18	**Obsidian**, Volcanic r ock	55.92	Unipolar, Discoid	Percentages calculated with the data from Chavaillon & Berthelet 2004 [12].

**Melka Kunture (Simbiro III, level B)**	Core, Chopper, Polyhedron, Heavy endscraper, Handaxe, Cleaver, Side scraper, Endscraper, Awl, Perforator, Notch, Denticulate, Anvil, Flake	769 artifacts. No debris collected (Chavaillon & Berthelet 2004 [12]).	Volcanic rock	27.18	**Obsidian**, Volcanic rock	55.92	Unipolar, Discoid	Percentages calculated with the data from Chavaillon & Berthelet 2004 [12].
**Melka Kunture (Simbiro III, level D)**	Core, Chopper, Polyhedron, Heavy endscraper, Handaxe, Cleaver, Side scraper, Notch, Denticulate, Anvil, Flake	113 artifacts. No debris collected (Chavaillon & Berthelet 2004 [12]).	Volcanic rock	23.89	**Obsidian**, Volcanic rock	76.26	NA	Percentages calculated with the data from Chavaillon & Berthelet 2004 [12].
**Barogali**	Core, Hammerstone, Chopper, Core scraper, Polyhedron, Bola, Flake, Scraper, Endscraper, Notch, Denticulate, Knife	569 artefacts (Berthelet 2001 [13]).	D	5.62	D	70.65	NA	Percentages calculated with the data from Berthelet 2001 [13].
**Gadeb 2B**	Core, Chopper, Polyhedron, Scraper, Handaxe, Cleaver, Proto biface, Flake, Debris	585 artefacts. Similar to Gadeb 2C but with more bifaces (handaxes and others) (Clark & Kurashina 1980 [14]).	Ba	10	Ba	81.34	NA	Percentages calculated with the data from Clark & Kurashina 1980 [14].
**Gadeb 2C**	Core, Chopper, Polyhedron, Scraper, Handaxe, Biface, Flake, Debris	622 artifacts (Clark & Kurashina 1980 [14]).	Ba	10	Ba	89	NA	Percentages calculated with the data from Clark & Kurashina 1980 [14].
**Gadeb 2E**	Core, Handaxe, Spheroid, Flake, Debris	Also percussive tools, LDT and LCT. 761 lithic artefacts (de la Torre 2011 [15]).	Ba	NA	Ba	48.5	NA	Percentage calculated with the data from de la Torre 2011 [15].
**Gadeb 8A**	Handaxe, Cleaver, Cleaver flake, Biface, Knife, Chopper, Polyhedron, Spheroid, Steep, Scraper, Blade	1849 artifacts (Clark & Kurashina 1980 [14]).	Ba	21	Ba	NA	NA	Percentage calculated with the data from Clark & Kurashina 1980 [14].
**Gadeb 8F**	Core, Handaxe, Spheroid, Flake, Debris	Also percussive tools, LDT and LCT. 179 artifacts (de la Torre 2011 [15]).	Ba	NA	Ba	65.36	NA	Percentage calculated with the data from de la Torre 2011 [15].

**Isenya (level V)**	Core, Biface, Handaxe, Cleaver, Core-axe, Polyhedron, Spheroid, Bola, Core	1073 artifacts. Also pebble tools (Roche et al. 1988 [16]).	**Ph**, Q	20.84	Ph, Q, Qzite, C	72.21	Unipolar	Percentages calculated with the data from Roche et al. 1988 [16].
**Isenya (level VIa)**	Polyhedron, Spheroid, Bola, Handaxe, Cleaver	3021 coordinated lithic artefacts. Data about the site: Roche et al. 1988 [16].	**Ph**, Volcanic rock	NA	NA	NA	NA	Data about the site: Roche et al. 1988 [16].
**Isenya (level VIb21)**	Polyhedron, Spheroid, Bola, Handaxe, Cleaver	1137 coordinated lithic artefacts. Data about the site: Roche et al. 1988 [16].	**Ph**, Q	NA	NA	NA	NA	Data about the site: Roche et al. 1988 [16].
**Olorgesailie Member 2**	Core, Flake, Debris, Handaxe, Cleaver, Scraper, Spheroid, Chopper, Core scraper	451 artifacts (Kleindienst 1961 [17]).	Ba, Volcanic rock, Q	15	NA	70	NA	Percentages calculated with the data from Kleindienst 1961 [17].
**Olorgesailie Member 3**	Core, Flake, Debris, Handaxe, Cleaver, Scraper, Spheroid, Chopper, Pick	465 artifacts (Kleindienst 1961 [17]).	Ba, Volcanic rock, Q	8	NA	75	NA	Percentages calculated with the data from Kleindienst 1961 [17].
**Olorgesailie Member 6**	Core, Flake, Debris, Handaxe, Cleaver, Scraper, Spheroid, Chopper, Pick, Core scraper, Knife, Disc	740 artifacts (Kleindienst 1961 [17]).	Ba, Volcanic rock, Q	18	NA	75	Discoid	Percentages calculated with the data from Kleindienst 1961 [17].
**Olorgesailie Member 7**	Core, Flake, Debris, Handaxe, Cleaver, Scraper, Spheroid, Chopper, Core scraper, Knife, Disc	205 artifacts (Kleindienst 1961 [17]).	Ba, Volcanic rock, Q	54	NA	31	Discoid	Percentages calculated with the data from Kleindienst 1961 [17].
**Olorgesailie Member 8**	Core, Flake, Debris, Handaxe, Cleaver, Scraper, Spheroid, Chopper, Knife	443 artifacts (Kleindienst 1961 [17]).	Ba, Volcanic rock, Q	35	NA	60	NA	Percentages calculated with the data from Kleindienst 1961 [17].
**Olorgesailie Member 9**	Core, Flake, Debris, Handaxe, Cleaver, Scraper, Spheroid, Chopper, Knife	215 artifacts (Kleindienst 1961 [17]).	Ba, Volcanic rock, Q	45	NA	45	NA	Percentages calculated with the data from Kleindienst 1961 [17].

**Olorgesailie Member 10**	Core, Flake, Debris, Handaxe, Cleaver, Scraper, Spheroid, Chopper, Core scraper, Knife, Disc	890 artifacts (Kleindienst 1961 [17]).	Ba, Volcanic rock, Q	15	NA	75	Discoid	Percentages calculated with the data from Kleindienst 1961 [17].
**Olorgesailie Member 11**	Core, Flake, Debris, Handaxe, Cleaver, Scraper, Spheroid, Chopper, Knife	434 artifacts (Kleindienst 1961 [17]).	Ba, Volcanic rock, Q	6	NA	86	NA	Percentages calculated with the data from Kleindienst 1961 [17].
**Isimila (K6)**	Core, Handaxe, Cleaver, Knife, Pick, Scraper, Core scraper, Chopper, Spheroid, Flake, Debris	892 artifacts (Howell et al. 1962 [18]).	Mylonite, G, Q, Qzite	49.89	NA	39.78	NA	Percentages calculated with the data from Howell et al. 1962 [18].
**Isimila (LJ6-7)**	Core, Handaxe, Cleaver, Knife, Core scraper, Disc, Chopper, Spheroid, Scraper, Debris, Flake	935 artifacts (Howell et al. 1962 [18]).	Mylonite, G, Q, Qzite	5.13	NA	84.81	Discoid	Percentages calculated with the data from Howell et al. 1962 [18].
**Isimila (K14)**	Core, Handaxe, Cleaver, Knife, Pick, Disc, Core scraper, Chopper, Spheroid, Flake, Debris	728 artifact (Howell et al. 1962 [18]).	**Cataclasite**, G, Q, Qzite	33.38	**Q**, Cataclasite, Qzite	61.4	Discoid	Percentages calculated with the data from Howell et al. 1962 [18].
**Isimila (H9-38)**	Core, Handaxe, Cleaver, Knife, Core scraper, Disc, Chopper, Spheroid, Scraper, Debris, Flake	173 artifacts (Howell et al. 1962 [18]).	Mylonite, G, Q, Qzite	45.66	NA	38.15	Discoid	Percentages calculated with the data from Howell et al. 1962 [18].
**Ounjougou**	Core, Polyhedron, Spheroid, Bola, Half ball, Flake, Chopper, Chopping tool	94 items (de Weyer 2017 [19]).	**Sa**, Qzite, Q	38.3	**Qzite, Q**, Sa	43.6	Bipolar on anvil	Percentages calculated with the data from de Weyer 2017 [19].
**Kabwe (Broken Hill)**	Bola	Also LDT. A small collection of artifacts probably associated with the skull (Klein 1973 [20]); Small tools in quartz (Clark 1981 [21]).	Q	NA	Q	NA	NA	Data from Clark 1981 [21].
**Broken Hill (Sangoan)**	Core, Handaxe, Chopper, Spheroid, Steep scraper, Flake, Disc	No debris collected. 32 artifacts (Clark 1959 [22]).	**Q**, Silcrete	56.25	Q	28.125	Discoid	*In situ*. Percentage Clark 1959 [22].

**Broken Hill (Rhodesian Acheulian)**	Core, Handaxe, Chopper, Spheroid, Bola, Cleaver, Side scraper, Endscraper, Notched scraper, Steep scraper, Flake, Disc, Debris	Only 1 debris collected. 164 artifacts (Clark 1959 [22]).	**Q**, L	23.17	Q, L, C	54.27	Discoid	Percentages calculated with the data from Clark 1959 [22].
**Broken Hill (Hope Fountain Bariant)**	Core, Chopper, Spheroid, Side scraper, Endscraper, Notched scraper, Steep scraper, Flake, Disc, Debris	106 artifacts (Clark 1959 [22]).	Q, Ironstone, C, Qzite	9.43	Q	81.13	Discoid	Percentages calculated with the data from Clark 1959 [22].
**Kalambo Falls (A4 rubble IIA)**	Core, Handaxe, Core-axe, Chopper, Pick, Core scraper, Bola, Protoburin, Scraper, Flake	967 artifacts (Sheppard & Kleindienst 1996 [23]).	**Qzite**, C	7.75	**C**, Qzite	78.59	NA	Percentages calculated with the data from Sheppard & Kleindienst [23].
**Kalambo Falls (A4 rubble IIB)**	Core, Cleaver, Disc, Core- axe, Chopper, Pick, Core scraper, Bola, Protoburin, Scraper, Flake	570 artifacts (Sheppard & Kleindienst 1996 [23]).	**Qzite**, C	7.54	**C**, Qzite	76.49	Discoid	Percentages calculated with the data from Sheppard & Kleindienst [23].
**Kalambo Falls (A5 Comp)**	Core, Handaxe, Knife, Cleaver, Disc, Core-axe, Chopper, Pick, Core scraper, Protoburin, Scraper, Disc, Flake, Bola	2371 artifacts (Sheppard & Kleindienst 1996 [23]).	**Qzite**, C	5.22	**C**, Qzite	86.17	Discoid	Percentages calculated with the data from Sheppard & Kleindienst [23].
**Cave of Hearths (Bed 1-3)**	Core, Biface, Cleaver, Chopper, Disc, Polyhedron, Scraper, Denticulate, Hammerstone, Manuport, Flake, Debris	2212 artifacts (McNabb et al. 2009 [24]).	**Qzite**, D, Q, C, Felsite	19.66	**Qzite**, D, Q, C, Felsite	53.75	Discoid	Percentages calculated with the data from McNabb et al. 2009 [24].
**Swartkrans (SWT-M1, SPRP excavation)**	Core, Chopper, Polyhedron, Flake, Notched scraper, Steep scraper, Hammerstone, Manuport, Debris	1849 artifacts (Kuman et al. 2018 [25]).	**Q,** Qzite	0.32	Q, C, Qzite	95	Bipolar, Opportunistic	Percentages calculated with the data from Kuman et al [25].
**Swartkrans (SWT-M1, LB)**	Chopper, Polyhedron, Side scraper, Endscraper, Bec, Flake, Core, Debris	402 artifacts (Clark 1993 [26]).	NA	0.99	**C, Q**, Qzite	64.43	Bipolar, Opportunistic	Percentages calculated with the data from Clark 1993 [26].
**Swartkrans (SWT-M2)**	Chopper, Polyhedron, Spheroid, Side scraper, Endscraper, Borer, Disc, Burin, Flake, Core, Debris	Most of the HDTs are P. 403 artifacts (Clark 1993 [26]).	Qzite, C, Q	4.71	Q, Qzite	25.81	Bipolar, Discoid, Opportunistic	Percentages calculated with the data from Clark 1993 [26].
**Swartkrans (SWT-M3)**	Double side scraper or cleaver, Chopper, Polyhedron, Spheroid, Bola, Disc, Core, Flake, Debris	72 artifacts (Clark 1993 [26]).	Qzite, Q	13.89	**Q**, Qzite	36.11	Bipolar, Discoid	Percentages calculated with the data from Clark 1993 [26].

**Vlakkraal Thermal springs**	Core, Point, Bifaced point, Side scraper, Endscraper, Serrated scraper, Burin, Polyhedron, Spheroid, Bola, Half ball	NA	NA	NA	NA	NA	Levallois	Data from Wells & Cooke 1942 [27].
**Windhoek**	Spheroid	NA	NA	NA	NA	NA	NA	NA
**Esere**	NA	NA	NA	NA	NA	NA	NA	NA
**Rhino Cave (Tsodilo Hills)**	Spheroid, Half ball	NA	NA	NA	NA	NA	NA	NA
**Corner Cave (Tsodilo Hills)**	Spheroid, Half ball	NA	NA	NA	NA	NA	NA	NA
**Kalkbank**	Core, Polyhedron, Bola, Upper grindstone, Side scraper, Endscraper, Flake, Debris	88 artifacts (Mason et al. 1958 [28]).	**Q**, Qzite	23.86	**Q**, D	53.41	NA	Also bone tools (Mason et al. 1958 [28]). Percentage calculated with the data from Mason et al. 1958 [28].
**Florisbad**	Core, Chopper, Chopping tool, Point hammer, Knife, Scraper, Polyhedron, Spheroid, Bola, Flake, Pounder, Anvil	157 artifacts (no debris recorded in the article) (Meiring 1956 [29]).	D	56.69	D	16.56	NA	Percentage calculated with the data from Meiring 1956 [29].
**Sterkfontein (Member 5)**	Spheroid	NA	NA	NA	NA	NA	NA	NA
**El Guettar**	Core, Flake, Debris, Point, Scraper, Notch, Endscraper, Chopper, Spheroid, Polyhedron	740 artifacts (Gruet 1950 [30]).	NA	NA	NA	NA	Discoid	Data from Gruet 1950 [30].
**Ain El Hallouf**	Polyhedron, Spheroid, Bola	NA	NA	NA	NA	NA	NA	NA
**Sidi Abderrahmane**	NA	NA	NA	NA	NA	NA	NA	NA
**Sidi Abderrahmane STIC**	Half ball, Polyhedron	NA	NA	NA	NA	NA	NA	NA
**Erg Tihodaine (Coll. Arambourg)**	Core, Uniface, Biface, Cleaver, Knife, Core scraper, Pick, Polyhedron, Spheroid	137 artifacts. No flake nor fragment collected. (Hocine 2016 [31]).	**Rhyolite**, **Q**, **Qzite**, Ph, Ba, Gneiss, Andesite, Microgranite	89.78	**Rhyolite**, **Qzite**, Gneiss, Andesite	7.3	Discoid	Percentages calculated with the data from Hocine 2016 [31].
**Tighennif I**	Core, Biface, Cleaver, Flake, Polyhedron	1227 artifacts. Also flaked pebbles (Djemmali 1985 [32]).	Sa, Qzite, L, F	NA	Sa, Qzite, L, F	NA	Kombewa	Percentages calculated with the data from Djemmali 1985 [32].
**Ain Hanech**	Core, Polyhedron, Spheroid, Hammerstone, Flake, Debris	NA	L, F	NA	**F**, L	NA	NA	Data from Sahnouni 1985 [33].

**Nzako Ambilo**	Core, Flake, Disc, Biface, Spheroid	256 artifacts. No hammerstone (Mestin 2018 [34]).	**Qzite**, Sa, Q	53.91	**Qzite**, Sa	37.89	Discoid, Opportunistic, Hierarchical, Levallois	Percentages calculated with the data from Mesfin 2018 [34].
**Nzako Kono**	Core, Flake, Debris, Disc, Biface, Hammerstone, Polyhedron, Spheroid	136 artifacts (Mestin 2018 [34]).	**Sa**, Qzite, Q	45.59	**Sa**, Qzite, Q	42.65	Discoid, Opportunistic, Hierarchical	Percentages calculated with the data from Mesfin 2018 [34].
**M'Piaka**	Core, Flake, Blade, Core, Disc, Core, Biface, Spheroid	218 artifacts (Mestin 2018 [34]).	**Sa**, C	61.47	**Sa**, C, Breccia	29.82	Levallois, Discoid, Laminar	Percentages calculated with the data from Mesfin 2018 [34].
**Hummal (Levels 17 & 18)**	Core, Chopper, Chopping tool, Polyhedron, Spheroid, Hammerstone, Pseudo scraper, Denticulate, Pseudo endscraper Debris, Flake	195 artifacts (n=87 in layer 17, n=108 in layer 18) (Le Tensorer et al. 2011 [35]).	L, F	6.97	F	83.08	Discoid	Percentages calculated with the data from Le Tensorer et al. 2011 [35].
**Shuwayhitiyah**	Chopper, Handaxe, Polyhedron, Spheroid, Proto biface, Biface, Discoid, Borer, Notch, Burin, Knife	NA	NA	NA	NA	NA	Discoid	Data from Petraglia 2003 [36].
**'Ubeidiya (III-20)**	Spheroid, Polyhedron, Chopper, Disc, Core, Scraper, Notch, Awl, Flake, Debris	NA	Ba, F, L	NA	F	NA	Discoid	Data from Shea 1999 [37].
**'Ubeidiya (III-22)**	Spheroid, Polyhedron, Chopper, Discoid, Biface, Pick, Core, Scraper, Notch, Denticulate, Awl, Flake, Debris	NA	Ba, F, L	NA	F	NA	Discoid	Data from Shea 1999 [37].
**Dursunlu**	Notch, Chopper, Polyhedron, Spheroid, Flake, Debris	135 modified quartz implements. No evidence of handaxe (Güleç et al. 2009 [38]). Maybe more, with possible unmodified artifacts.	Q	3.7	Q	84.44	Bipolar, Bipolar on anvil	Percentages calculated with the data from Gürleç et al. 2009 [38]. "we cannot exclude the possibility that the absence of evidence for bifacial technology is due to the limited size of the existing collection […] and to the small size and poor quality of the raw material" (Güleç et al. 2009 [38]).
**North of Bridge Acheulian (NBA)**	Core, Handaxe, Cleaver, Spheroid, Flake	Mainly handaxes and cleavers (Sharon et al. 2010 [39]).	**Ba**, F	NA	Ba, F	NA	NA	Data from Sharon et al. 2010 [39].

**Latamné**	Handaxe, Cleaver, Knife, Chopper, Core scraper, Spheroid, Flake	1831 artifacts (Clark 1966 [40]).	**F**, L, Ba	8.08	F	85.2	Discoid	Percentages calculated with the data from Clark 1966 [40].
**Joubb Jannine II**	Core, Chopper, Handaxe, Chopping tool, Pick, Spheroid, Polyhedron, Scraper, Burin, Percoir, Knife, Endscraper, Notch, Denticulate, Rabot, Flake	NA	F, L	NA	F, L	NA	Discoid, Proto Levallois	Latamne faciès. Selection of tools during the archaeological collect. Data: Yazbeck 2002 [41].
**Khalliyé Sud**	Core, Pick, Spheroid, Handaxe, Flake	96 artefacts including flakes (Yazbeck 2002 [41]).	NA	41.67	NA	19.79	NA	Percentages calculated with with the data from Yazbeck 2002 [41].
**Wadi Fatimah**	Core, Biface, Polyhedron, Chopper, Handaxe, Cleaver, Pick, Trihedral, Scraper, Discoid, Knife, Flake	2227 artifacts (Petraglia 2003 [36]).	Andesite	NA	Andesite	NA	Levallois, Discoid	The assemblage looks like Dawadmi. Data from Petraglia 2003 [36].
**Revadim Quarry (Area D)**	Spheroid	NA	**F**, L	NA	F	NA	NA	Many lithics found with the PSBs (Barkai & Gopher 2016 [42]).
**Evron Quarry**	Core, Borer, Denticulate, Notch, Burin, Scraper, Endscraper, Polyhedron, Chopper	NA	NA	NA	NA	NA	NA	Data from Tchernov et al. 1994 [43]. Bar-Yosef 1994 [44]: absence of bifaces because of "the spatial distribution of hominid activities."

**Evron East**	Handaxe, Spheroid	2 handaxes, small flake industry and several S (Shemer & Barzilai 2017 [45]).	F, L	NA	F	NA	NA	Shemer & Barzilai 2017 [45] data.
**Saffaqah**	Discoid, Handaxe, Cleaver, Spheroid, Polyhedron, Knife, Pick, Trihedral, Chopper, Biface, Scraper, Core-axe, Borer, Burin, Notch, Chisel, Core, Blade, Hammerstone, Flake	11360 artifacts (Petraglia 2003 [36]).	NA	NA	NA	NA	Discoid, Levallois	Data from Petraglia 2003 [36].
**Qesem Cave**	Blade, Scraper, Biface, Handaxe, Spheroid, Half ball, Denticulate, Side scraper, Endscraper, Double scraper, Burin, Knife, Flake	NA	**F**, L	NA	F	NA	Quina	Data from Gopher et al. 2005 [46].
**Bezez**	Polyhedron	NA	NA	NA	NA	NA	NA	NA
**Ma'ayan Barukh**	Core, Handaxe, Cleaver, Disc, ChoppingTool, Scraper, Endscraper, Chopper, Hammerstone, Burin, Notch, Flake, Knife, Pick, Debris, Denticulate	3775 artifacts (Stekelis & Gilead 1966 [47]).	**F,** Ba	70.01	**F**, Ba	21.09	Discoid, Quina, Levallois	Scrapers and endscrapers made on flakes (Stekelis & Gilead 1966 [47]).
**Kaletepe Deresi 3 (Level III)**	Core, Polyhedron, Scraper, Flake, Debris	354 artifacts (Slimak et al. 2007 [48]).	Andesite	1.13	Obsidian	85.31	Levallois, Discoid	24 tools aren't included into the percentages here (no precision if they are HDTs or LDTs). Data from Slimak et al. 2008 [48].
**Kaletepe Deresi 3 (Level III/IV)**	Core, Polyhedron, Chopper, Chopping tool, Flake, Debris	106 artifacts (Slimak et al. 2007 [48]).	NA	4.72	NA	73.58	NA	7 tools aren't included into the percentages here (no precision if they are HDTs or LDTs). Data from Slimak et al. 2008 [48].

**Kaletepe Deresi 3 (Level IV)**	Core, Polyhedron, Biface, Cleaver, Scraper, Denticulate, Notch, Handaxe, Chopper, Chopping tool, Flake, Debris	2315 artifacts (Slimak et al. 2007 [48]).	Andesite, Rhyolite	7.86	Andesite, Rhyolite	78.01	Discoid, Opportunistic	Discoidal cores, simple flake cores (Slimak et al. 2007 [48]). 85 tools aren't included into the percentages here (no precision if they are HDTs or LDTs). Data from Slimak et al. 2008 [48].
**Kaletepe Deresi 3 (Level V)**	Core, Polyhedron, Biface, Cleaver, Handaxe, Chopper, Chopping tool, Point, Scraper, Notch, Flake, Debris	532 artifacts (Slimak et al. 2007 [48]).	Ba, Andesite, Obsidian	13.72	NA	73.49	NA	19 tools aren't included into the percentages here (no precision if they are HDTs or LDTs). Data from Slimak et al. 2008 [48].
**Kaletepe Deresi 3 (Level Vam)**	Core, Polyhedron, Chopper, Chopping tool, Flake, Debris	91 artifacts (Slimak et al. 2007 [48]).	Obsidian, Andesite	16.48	NA	64.84	NA	8 tools aren't included into the percentages here (no precision if they are HDTs or LDTs). Data from Slimak et al. 2008 [48].
**Kaletepe Deresi 3 (Level V')**	Core, Polyhedron, Flake, Debris	58 artifacts (Slimak et al. 2007 [48]).	Obsidian, Andesite	3.45	NA	93.10	NA	Data from Slimak et al. 2008 [48].
**Kaletepe Deresi 3 (Level VI')**	Core, Polyhedron, Handaxe, Biface, Uniface, Cleaver, Scraper, Denticulate, Flake, Debris	353 artifacts (Slimak et al. 2007 [48]).	Ba, Andesite, Obsidian, Rhyolite	9.63	NA	81.02	NA	Data from Slimak et al. 2008 [48].
**Kaletepe Deresi 3 (Level VII)**	Core, Polyhedron, Handaxe, Flake, Debris	73 artifacts (Slimak et al. 2007 [48]).	Ba, Andesite, Obsidian, Rhyolite	21.92	NA	75.34	NA	1 tool isn't included into the percentages here (no precision if they are HDTs or LDTs). Data from Slimak et al. 2008 [48].
**Kaletepe Deresi 3 (Level VIII)**	Core, Polyhedron, Handaxe, Chopper, Chopping tool, Flake, Debris	152 artifacts (Slimak et al. 2007 [48]).	Ba, Andesite, Obsidian, Rhyolite	11.18	NA	86.84	NA	Data from Slimak et al. 2008 [48].
**Kaletepe Deresi 3 (Level IX)**	Polyhedron, Chopper, Chopping tool, Flake, Debris	104 artifacts (Slimak et al. 2007 [48]).	Ba, Andesite, Obsidian, Rhyolite	6.73	NA	93.27	NA	Data from Slimak et al. 2008 [48].
**Kaletepe Deresi 3 (Level X)**	Polyhedron, Flake, Debris	30 artifacts (Slimak et al. 2007 [48]).	Ba, Andesite, Obsidian, Rhyolite	6.67	NA	93.33	NA	Data from Slimak et al. 2008 [48].
**Kaletepe Deresi 3 (Level XI)**	Core, Polyhedron, Flake, Debris	79 artifacts (Slimak et al. 2007 [48]).	Ba, Andesite, Obsidian, Rhyolite	16.46	NA	81.01	NA	Data from Slimak et al. 2008 [48].

**Kaletepe Deresi 3 (Level XII)**	Polyhedron, Flake, Debris	46 artifacts (Slimak et al. 2007 [48]).	Ba, Andesite, Obsidian, Rhyolite	6.52	NA	93.48	NA	Data from Slimak et al. 2008 [48].
**Santa Ana Cave (Unit 1)**	Core, Polyhedron, Flake, Handaxe, Pick, Cleaver on flake, Denticulate, Side scraper, Notch, Denticulated point, Hammerstone	578 artifacts (García-Vadillo et al. 2022 [49])	**Qzite**, Q	NA	**Q**, Qzite	15.79	Unipolar, Bipolar, Bipolar on anvil	Data from García-Vadillo et al. 2022 [49].
**Barranco León**	Core, Polyhedron, Spheroid, Core scraper, Flake, Debris	Also percussive tools. 2154 artifacts (Titton et al. 2020 [50]).	L	2.9	**F**, L	91.8	Bipolar on anvil, Unipolar	Titton et al. 2020 [50]: "two very different groups of tools, made from distinct raw materials. On the one hand, a small-sized toolkit knapped from Jurassic flint, […], on the other hand, a large-sized limestone toolkit that is mainly linked to percussive activities". (Titton et al. 2020 [50]). Percentages calculated with the data from Titton et al. 2020 [50].
**Bois-de-Riquet (Unit 4)**	Core, Hammerstone, Polyhedron, Flake, Notch, Denticulate	Also macro-shaped tools. 235 artifacrs (Bourguignon et al. 2016 [51]).	Ba	16.17	**Ba**, Q, Aplite	35.74	Bipolar on anvil	Percentages calculated with the data from Bourguignon et al. 2016 [51].
**Ca' Belvedere di Monte Poggiolo**	Core, Flake, Polyhedron, Lateral scraper, Denticulate	1311 artifacts. Also pebble tools (Terradillos Bernal & Moncel 2004 [52]).	F	0.8	F	89.85	Unipolar, Bipolar	Percentages calculated with the data from Terradillos Bernal & Moncel 2004 [52].
**Dorn-Dürkheim 3**	Core, Scraper, Polyhedron, Flake	10 artifacts (Fiedler et al. 2019 [53]).	Qzite	10	Qzite, Q, Rhyolite	80	Bipolar on anvil	Data and percentages from Fiedler et al. 2019 [53].
**La Noira (Stratum c)**	Core, Cleaver, Polyhedron, Biface, Chopper, Scraper, Point, Flake, Debris	537 artifacts (Moncel et al. 2021 [54]). Cleavers on flakes.	**Milestone**, Chert, F, Qzite	16.39	**Milestone**, Chert, F, Rhyolite	72.81	Levallois like, Discoid	Cores are mostly centripetal but also orthogonal, trifacial, peripheral, multidirectional, Percentages calculated with the data of Moncel et al. 2021 [54].
**Caune de l'Arago (Unit H1,2,3)**	Core, Flake, Blade, Debris	1737 artifacts. Also pebble tools (Barsky 2001 [55]).	Q, Qzite, L, F, Sa	2.8	Q, Qzite, L, F, Sa	79.3	NA	Percentages calculated with the data of Barsky 2001 [55].

**Caune de l'Arago (Unit G)**	Cleaver, Handaxe, Core, Flake, Blade, Debris	20656 artifacts. Also pebble tools (Barsky 2001 [55]).	Q, Qzite, L, F, Sa	3.4	Q, Qzite, L, F, Sa	81.6	NA	Percentages calculated with data from Barsky 2001 [55].
**Caune de l'Arago (Unit E)**	Cleaver, Handaxe, Core, Flake, Blade, Debris	1703 artifacts. Also pebble tools (Barsky 2001 [55]).	Q, Qzite, L, F, Sa	3.2	Q, Qzite, L, F, Sa	79.2	NA	Percentages calculated with the data from Barsky 2001 [55].
**Caune de l'Arago (Unit D)**	Cleaver, Handaxe, Core, Flake, Blade, Debris	3019 artifacts. Also pebble tools (Barsky 2001 [55]).	Q, Qzite, L, F, Sa	1.6	Q, Qzite, L, F, Sa	88.4	NA	Percentages calculated with the data from Barsky 2001 [55].
**Treugol'Naya Cave (assemblage II)**	Hammerstone, Core, Polyhedron, Spheroid, Side scraper, Endscraper, Chopper, Chopping tool, Pick, Biface, Rabot, Core scraper, Proto biface, Flake, Debris	70 artifacts (Doronichev et al. 2007 [56]).	**L**, F	45	L, F	33	NA	Percentages calculated with the data from Doronichev et al. 2007 [56].
**Duclos (0)**	Hammerstone, Debris, Flake, Core, Denticulate, Chopper, Pick, Biface, Cleaver, Polyhedron	91 artifacts. Also pebble tools (Cologne 2012 [57]).	Qzite	17.58	Qzite	67.03	Discoid, Bipolar on anvil, Unipolar	Caution: maybe overrepresentation of HDT because of water runoff.<br><br>Data and percentage from Cologne 2012 [57].
**Duclos (Ensemble IV)**	Hammerstone, Debris, Flake, Core, Scraper, Denticulate, Chopper, Pick, Biface, Cleaver, Polyhedron	469 artifacts. Also pebble tools (Cologne 2012 [57]).	Qzite	11.7	Qzite	73.7	Discoid, Bipolar on anvil, Unipolar	Caution: maybe overrepresentation of HDT because of water runoff.<br><br>Data and percentage from Cologne 2012 [57].
**Duclos (Ensemble III)**	Hammerstone, Debris, Flake, Core, Scraper, Denticulate, Chopper, Pick, Biface, Cleaver, Polyhedron	835 artifacts. Also pebble tools (Cologne 2012 [57]).	Qzite	16.59	Qzite	67.3	Discoid, Bipolar on anvil, Unipolar	Caution: maybe overrepresentation of HDT because of water runoff.<br><br>Data and percentage from Cologne 2012 [57].

**Septsos**	Polyhedron, Hammerstone, Core, Biface, Cleaver, Flake, Debris	747 objects (Fourloubey 2012 [58]). And pebble tools.	**Qzite**, F, *Lydian*	31.8	**Qzite**, F, Shiste, Q, *Lydian*	53.1	Discoid, Unipolar	Data from Fourloubey 2012 [58].
**Cerveny Kopec**	Polyhedron, Flake	4 artifacts. Also pebble tools (Svoboda et al. 1998 [59]).	Q	50	Qzite	50	NA	Data from Svoboda et al. 1998 [59].
**Bañugues (Asturias del Esferoid)**	Core, Blade, Point, Spheroid, Scraper, Endscraper, Perforator, Knife, Notch, Denticulate, Biface, Cleaver, Trihedral pick, Asturian pick, Flake, Debris	774 artifacts. Also pebble tools (Rodríguez Asencio & Flor Rodríguez 1979 [60]).	Qzite	44.32	Qzite	52.71	Levallois	Percentages calculated with the data from Rodríguez Asensio & Flor Rodríguez 1979 [60].
**Tourville-la-Rivière (level D2)**	Core, LevalloisBlade, Polyhedron, Flake, Debris	216 artifacts (Cliquet 2010 [61]).	F	0.93	F	79.6	Unipolar, Levallois	Only fresh items are considered here since others might come from D1 level (Cliquet 2010 [61]). Percentages calculated with the data from Cliquet 2010 [61].
**Chez-Pinaud Jonzac (US 22)**	Core, Scraper, Polyhedron, Half ball, Flake, Debris	Mostly scrapers. 6941 artifacts, among which 4052 little flakes (Claud et al. 2012 [62]).	L	0.04	F	NA	NA	Data from Claud et al. 2012 [62].
**La Quina (level 8)**	Core, Scraper, Hammerstone, Spheroid, Polyhedron, Bola, Denticulate, Notch, Bec,	20314 artifacts (5874 industries, 14440 debris), Park 2007 [63].	**L**, Q	0.05	**F**, Q, Chalcedony	NA	Levallois, Discoid	Data from Park 2007 [63].
**Festons (Rebières valley)**	Hammerstone, Polyhedron, Half ball, Anvil, Squared block, Scraper, Endscraper, Burin, Endscraper, Flake, Debris	Also pebble tools (Pittard & de Saint-Périer 1955 [64]).	F, L, Q	NA	NA	NA	Discoid	Industry on bones too. Data from Pittard & de Saint-Périer 1955 [64].
**Sablière Rambour (Villers-Bocage)**	Flake, Point, Scraper, Blade, Burin, Polyhedron	(Agache 1958 [65]).	F, Sa	NA	NA	NA	Levallois	NA

**Isle-Adam (sablière de**	Spheroid	NA	Sa	NA	NA	NA	NA	NA
**Coll de la Guille (Terrasses du Roussillon)**	Chopper, Chopping tool, Polyhedron, Handaxe, Discoid, Epannele	NA	Q	NA	NA	NA	NA	Data from Collina-Girard 1986 [66].
**Mas Ferreol (Terrasses du Roussillon)**	Core, Chopper, Chopping tool, Flake, Polyhedron, Scraper, Denticulate, Notch	127 artefacts (Garcia Garriga 2014 [67]).	Q	NA	Q	NA	NA	Data from Collina-Girard 1986 [66], Garcia Garriga 2014 [67].
**Mas Ferrer (Terrasses du Roussillon)**	Chopper, Polyhedron	NA	Q	100	NA	0	NA	Data from Collina-Girard 1986 [66].
**Le Puech de la Boule (Terrasses du Roussillon)**	Chopper, Polyhedron	NA	Q	100	NA	0	NA	Data from Collina-Girard 1986 [66].
**Mas Bruno (Terrasses du Roussillon)**	Polyhedron	NA	Q	NA	NA	NA	NA	NA
**Cabestany général (Terrasses du Roussillon)**	Chopper, Chopping tool, Discoid, Epannele, Handaxe, Polyhedron	NA	NA	NA	NA	NA	NA	Data from Collina-Girard 1986 [66].
**La Llabanère (Terrasses du Roussillon)**	Chopper, Chopping tool, Polyhedron, Handaxe, Discoid, Epannele	NA	NA	NA	NA	NA	NA	Data from Collina-Girard 1986 [66].
**Singi Talav (Layer 3)**	Core, Side scraper, Denticulated scraper, Notch, Bec, Endscraper, Convergent point, Convergent chopper, Chopping tool, Double chopper / chopping tool, Discoid, Spheroid, Polyhedron, Flake, Hammerstone, Handaxe, Proto handaxe, Debris	Some choppers, discoid, P and S are "micro". 401 artifacts (Gaillard & Rajaguru 2017 [68]).	**Qzite**	NA	**Qzite**, Q, Schiste	NA	Discoid	Data from Gaillard & Rajaguru 2017 [68].
**Singi Talav (Layer 4)**	Core, Denticulate, Burin, Side scraper, Denticulated scraper, Notch, Bec, Endscraper, Convergent point, Chopping tool, Double chopper / chopping tool, Discoid, Spheroid, Polyhedron, Flake, Hammerstone, Handaxe, Proto handaxe, Debris	Some choppers, discoid, P and S are "micro". 891 artifacts (Gaillard & Rajaguru 2017 [68]).	**Qzite**	NA	**Qzite**, Q, Schiste	NA	Discoid	Data from Gaillard & Rajaguru 2017 [68].
**Torajunga**	Core, Flake, Blade, Leaf point, Tanged point, Handaxe, Cleaver, Pick, Polyhedron, Spheroid, Debris	670 artifacts (Behera & Thakur 2019 [69]).	**Qzite**, Silicified, Dolerite	15.76	**Qzite**, C, Silicified, Dolerite, Sa	69.86	Levallois, Discoid	Data from Behera & Thakur 2019 [69].

**Chirki Nevasa**	Core, Debris, Flake, Cleaver, Knife, Core scraper, Handaxe, Pick, Borer, Biface, Chopper, Polyhedron, Discoid, Bola	NA	Ba, silicate, Q	NA	NA	NA	Discoid	Data from Corvinus 1983 [70].
**Atit 2**	Spheroid	NA	Ba	NA	NA	NA	NA	NA
**Zhoukoudian 1 (Layer 1-3)**	Core, Flake, Hammerstone, Anvil, Side scraper, Point, Awl, Burin, Chopper, Chopping tool, Spheroid, Debris	3484 lithics (Li 2016 [71]).	NA	0.69	NA	97.42	Bipolar, Bipolar on anvil	Percentages calculated with the data from Li 2016 [71].
**Zhoukoudian 1 (Layer 4-5)**	Core, Flake, Hammerstone, Anvil, Side scraper, Point, Awl, Burin, Chopper, ChoppingTool, Spheroid, Debris	6651 lithics (Li 2016 [71]).	NA	0.5	NA	95.97	Bipolar	Percentages calculated with the data from Li 2016 [71].
**Zhoukoudian 1 (QII)**	Core, Flake, Hammerstone, Side scraper, Point, Burin, Chopper, Chopping tool, Spheroid, Debris	1244 lithics (Li 2016 [71]).	NA	1.13	NA	96.7	Bipolar	Percentages calculated with the data from Li 2016 [71].
**Zhoukoudian 1 (Layer 8-9)**	Core, Flake, Hammerstone, Side scraper, Point, Burin, Chopper, Chopping tool, Spheroid, Debris	1336 lithics (Li 2016 [71]).	NA	5.01	NA	89.22	Bipolar, Bipolar on anvil	Percentages calculated with the data from Li 2016 [71].
**Liangshan Longgangsi**	Core, Hammerstone, Polyhedron, Spheroid, Biface, Flake, Debris, Bola	121 lithics (Bodin 2011 [72]).	**Q**, Volcanic rock, Qzite, Sa	40.8	**Q**, Volcanic rock, Qzite	23.8	Discoid, Bipolar	Data from Bodin 2011 [72].
**Dingcun**	Debris, Handaxe, Cleaver, Pick, Core denticulate, Core scraper, Scraper, Chopper, Spheroid, Bola, Notch, Borer, Denticulate, Flake, Debris	1177 artifacts described among the 2005 discovered in 1953-1954 (Yang et al. 2014 [73]).	**Hornfels**, L, Qzite, Sa, C	5.61	**Hornfels**, C, Q	69.32	NA	Percentages calculated with the data from Yang et al. 2014 [73].
**Gongwangling**	Core, Flake, Scraper, Spheroid, Chopper, Handaxe, Pick	26 artifacts in all the sequence (Wang & Lu 2016 [74]).	**Qzite**, Q	NA	**Qzite**, Q	NA	NA	Data from Wang & Lu 2016 [74].
**Ganyu**	Core, Manuport, Hammerstone, Flake, Chopper, Spheroid, Handaxe, Pick Scraper, Point	550 artifacts, mostly cores (n=347) (Wang et al. 2014 [75]).	**Qzite**, Q, Greywacke, L, Igneous rock	8.18	**Q, Qzite**, Igneous rock, Greywacke	13.64	Bipolar	Percentages calculated with the data from Wang et al. 2014 [75].

**Maling 2A**	Core, Hammerstone, Flake, Debris, Chopper, Disc, Polyhedron, Core scraper, Handaxe, Cleaver	1026 lithics (Pei et al. 2015 [76]).	**Qzite**, Q, Other	6.43	**Qzite**, Q, Other	93.66	Discoid	Percentages calculated with the data from Pei et al. 2015 [76].
**Shuigou-Huixinggou**	Core, Handaxe, Cleaver, Pick, Chopper, Spheroid, Flake	94 artifacts (Li et al. 2017 [77]).	NA	NA	NA	NA	NA	Data from Li et al. 2017 [77].
**Zhoukoudian 15**	Core, Flake, Debris, Spheroid, Hammerstone, Scraper, Notch, Denticulate, Burin, Point, Awl, Chopper, Chopping tool, Anvil, Cleaver	6870 artifacts (Gao 2000 [78]).	Q, Igneous rock, Sa, Qzite	0.25	**Q**, Igneous rock, F, Sa	93	Discoid, Bipolar, Bipolar on anvil, Opportu-nistic	Percentages calculated with the data from Gao 2000 [78].
**Xujiayao**	Scraper, Point, Awl, Burin, Spheroid, Flake, Core, Notch, Borer, Denticulate, Bola	>20,000 artifacts (Liu et al. 2013 [79]).	Qzite, Dolomite	7.6	Q, C, Qzite, Ba	74	Bipolar, Discoid	Data from Liu et al. 2013 [79], Clark & Schick 1988 [80], Yang et al. 2019 [81].
**Lingjing (Layer 11, lower part of layer 10)**	Core, Scraper, Notch, Denticulate, Point, Borer, Burin, Chopper, Spheroid, Flake, Debris, Hammerstone	14862 artefacts (Li et al. 2019 [81]).	**Q**, Qzite	0.03	**Q**, Qzite	78.07	Discoid, Bipolar, Opportu-nistic	Percentages calculated with the data from Li et al. 2019 [82].
**Hsuchiyao**	Core, Flake, Scraper, Point, Burin, Borer, Polyhedron, Spheroid, Bola	> 25000 artifacts (Chi 1979 [82]).	NA	NA	NA	NA	NA	Data from Chi 1979 [83].
**Diaozhai**	Core, Flake, Chopper, Spheroid, Pick, Scraper, Point, Debris	114 artifacts (Wang et al. 2014 [75]).	NA	11.41	NA	34.22	NA	Data from Wang et al. 2014 [75].
**Jijiawan**	Core, Flake, Spheroid, Handaxe, Pick, Debris	13 artifacts (Wang et 2014 [75]).	NA	38.46	NA	30.77	NA	Data from Wang et al. 2014 [75].
**Houjiapu**	Core, Chopper, Spheroid, Scraper, Debris	21 artifacts (Wang et 2014 [75]).	NA	33.33	NA	14.28	NA	Data from Wang et al. 2014 [75].
**Zhoupo (Locality 95LP07)**	Core, Flake, Chopper, Pick, Biface, Handaxe, Spheroid, Debris, Polyhedron, Scraper, Point	516 artifacts (Bodin 2011 [72]).	NA	11.82	NA	27.32	NA	Data from Bodin 2011 [72].
**Mansuri (Locality 1)**	Biface, Core, Chopper, Flake	1240 artifacts in the 3rd layer. Also flake tools (Bodin 2011 [72]).	**Q**, Qzite	NA	**Q**	NA	NA	Data from Bodin 2011 [72].
**Jeongok-Ri (surface)**	Core, Hammerstone, Flake, Notch, Polyhedron, Chopping tool, Pick, Handaxe	NA	Q, Qzite	NA	Q, Qzite	NA	Unipolar, Bipolar on Anvil, Bipolar	Data from de Lumley et al 2011 [84].
**Jeongok-Ri (Layer 1)**	Core, Polyhedron	NA	Q	NA	NA	NA	Unipolar	Data from de Lumley et al 2011 [84].

**Jeongok-Ri (Layer 2)**	Core, Flake, Notch, Scraper, Endscraper, Chopper, Polyhedron	NA	Q, Qzite	NA	Q, Qzite	NA	Discoid, Bipolar on anvil, Unipolar	Data from de Lumley et al. 2011 [84].
**Jeongok-Ri (Layer 3)**	Flake, Notch, Bec, Scraper, Polyhedron	NA	Q	NA	**Q**, Qzite	NA	Bipolar on anvil	Data from de Lumley et al. 2011 [84].
**Jangnamgyo (surface)**	Core, Rabot, Polyhedron, Handaxe	NA	Q, Qzite	NA	Q, Qzite	NA	Bipolar, Bipolar on anvil	Data from de Lumley et al. 2011 [84].
**Jangnamgyo (Level 3)**	Core, Flake, Chopping tool, Rabot, Pick, Biface, Handaxe, Cleaver, Polyhedron	NA	Q, Qzite	NA	Q, Qzite	NA	Bipolar, Unipolar, Bipolar on anvil	Data from de Lumley et al. 2011 [84].
**Ngebung**	Chopper, ChoppingTool, Cleaver, Bola, Polyhedron, Flake, Core scraper, Spheroid	According to Moncel et al. 2018 [84], early appearance of bone tools (chopper from Stegodon tusk).	Andesite, Q	NA	NA	NA	NA	Data from Moncel et al. 2018 [85].
**Banjarejo**	Polyhedron, Bola	NA	NA	NA	NA	NA	NA	Data from Meilinda 2017 [86].
**Matar**	Bola, Polyhedron, Spheroid, Flake, Hammerstone, Denticulate, Notch, Endscraper, Percoir, Bifacial point, Core	112 artifacts (Fauzi et al. 2016 [86]).	Ba, Andesite	NA	Chalecedony, C	NA	Unipolar, Bipolar	Data from Fauzi et al. 2016 [87].
**Solo**	Chopper, Chopping tool, Polyhedron, Spheroid, Bola, Denticulate, Scraper, Flake	NA	NA	NA	NA	NA	NA	Also development of bone tools. Data from Fauzi et al. 2016 [87].
**Baksoko River**	Chopper, Chopping tool, Handaxe, Cleaver, Bola	NA	NA	NA	NA	NA	NA	Data from Fauzi et al. 2016 [87].

References

1. Cueva-Temprana A, Lombao D, Soto M, Itambu M, Bushozi P, Boivin N, Petraglia M, Mercader J. Oldowan technology amid shifting environments ~2.03-1.83 million years ago. Front Ecol Evol. 2022; 10: 788101.
2. Sahnouni M. Etude comparative des galets taillés polyédriques, subsphériques et sphériques des gisements d'Ain Hanech (Algérie orientale) et d'Olduvai (Tanzanie). Anthropologie. 1993; 97(1) : 51-68.
3. Mora R, de la Torre I. Percussion tools in Olduvai Beds I and II (Tanzania): implications for early human activities. J Anthropol Archaeol. 2005; 24: 179-92.
4. Pante MC, de la Torre I. A hidden treasure of the Lower Pleistocene at Olduvai Gorge, Tanzania: the Leakey HWK EE assemblage. J Hum Evol. 2018 Jul; 120: 114-39.
5. Sánchez-Yustos P, Diez-Martín F, Díaz I, Fraile C, Uribelarrea D, Mabulla A, et al. What comes after the Developed Oldowan B debate? Techno-economic data from SHK main site (Middle Bed II, Olduvai Gorge, Tanzania). Quat Int. 2019; 526: 67-76.
6. Diez-Martín F, Sánchez P, Domínguez-Rodrigo M, Mabulla A, Barba R. Were Olduvai hominins making butchering tools or battering tools? Analysis of a recently excavated lithic assemblage from BK (Bed II, Olduvai Gorge, Tanzania). J Anthropol Archaeol. 2009 Sep; 28(3): 274-89.
7. Santonja M, Panera J, Rubio-Jara S, Pérez-González A, Uribelarrea D, Domínguez-Rodrigo M et al. Technological strategies and the economy of raw materials in the TK (Thiongo Korongo) lower occupation, Bed II, Olduvai Gorge, Tanzania. Quat Int. 2014; 322-323: 181-208.
8. Berthelet A, Chavaillon J. Prehistoric archaeology. The site of Karre I: Karre I. In: Chavaillon J, Piperno M, editors. Studies on the Early Paleolithic site of Melka Kunture, Ethiopia. Florence: Origines; 2004. p. 211-51.
9. Gallotti R. An older origin for the Acheulean at Melka Kunture (Upper Awash, Ethiopia): techno-economic behaviours at Garba IVD. J Hum Evol. 2013; 65: 594-620.
10. Chavaillon J. Prehistoric archaeology. The site of Gombore I: discovery, geological introduction and study of percussion material and tools on pebble. In: Chavaillon J, Piperno M, editors. Studies on the Early Paleolithic site of Melka Kunture, Ethiopia. Florence: Istituto Italiano di Preistoria e Protostoria; 2004. p. 253-369.
11. Mussi M, Altamura F, Di Bianco L, Bonnefille R, Gaudzinski-Windheuser S, Geraads D et al. After the emergence of the Acheulean at Melka Kunture (Upper Awash, Ethiopia): from Gombore IB (1.6 Ma) to Gombore Iγ (1.4 Ma), Gombore 1σ (1.3 Ma) and Gombore II OAM Test Pit C (1.2 Ma). Quat Int. Forthcoming.
12. Chavaillon J, Berthelet A. The archaeological sites of Melka Kunture. In: Chavaillon J, Piperno M, editors. Studies on the Early Paleolithic site of Melka Kunture, Ethiopia. Florence: Istituto Italiano di Preistoria e Protostoria; 2004. p. 25-80.
13. Berthelet A**.** L'outillage lithique du site de dépeçage à *Elephas recki ileretensis* de Barogali (République de Djibouti). C R Acad Sci IIa. 2001 Mar; 332(6): 411-6.
14. Clark JD, Kurashina H. New Plio-Pleistocene archaeological occurrences from the plain of Gadeb, Upper Webi Shebele basin, Ethiopia, and a statistical comparison of the Gadeb sites with other Early Stone Age assemblage. Anthropologie 1980; 18(2-3): 161-87.
15. De la Torre I. The Early Stone Age lithic assemblages of Gadeb (Ethiopia) and the Developed Oldowan/early Acheulean in East Africa. J Hum Evol. 2011; 60: 768-812.
16. Roche H, Brugal JP, Lefevre D, Ploux S, Texier PJ. Isenya: état des recherches sur un nouveau site acheuléen d'Afrique orientale. Afr Archaeol Rev. 1988; 6(1): 27-55.
17. Kleindienst MR. Variability within the Late Acheulian assemblage in Eastern Africa. South African Archaeological Society 1961 Jun;

16(62): 35-52.

18. Howell FC, Cole GH, Kleindienst MR, Haldemann EG. Isimila: an Acheulian occupation site in the Iringa Highlands, Southern Highlands Province, Tanganyika. Musée Royal de l'Afrique centrale 1962; 43-81.

19. De Weyer L. An Early Stone Age in Western Africa? Spheroids and polyhedrons at Ounjougou, Mali. Journal of Lithic Studies 2017; 4(1).

20. Klein RG. Geological antiquity of Rhodesian Man. Nature 1973 Aug; 244: 311-2

21. Clark JD. Prehistory in southern Africa. In: Ki-Zerbo J, editor. General history of Africa, vol. 1: methodology and African Prehistory. London: Heinemann; 1981. p. 487-529.

22. Clark JD. Further excavations at Broken Hill, Northern Rhodesia. The Journal of the Royal Anthropological Institute of Great Britain and Ireland 1959 Jul-Dec; 89(2): 201-32.

23. Sheppard PJ, Kleindienst MR. Technological change in the Earlier and Middle Stone Age of Kalambo Falls (Zambia). Afr Archaeol Rev. 1996; 13(3): 171-96.

24. McNabb J, Sinclair A., Wadley L, Maguire J, Latham A, Herries A et al. The Cave of Hearths: Makapan Middle Pleistocene research project: field research by Antony Sinclair and Patrick Quinney, 1996-2001. Oxford: Archaeopress; 2009. 193 p.

25. Kuman K, Sutton MB, Pickering TR, Heaton JL. The Oldowan industry from Swartkrans cave, South Africa, and its relevance for the African Oldowan. J Hum Evol. 2018; 123: 52-69.

26. Clark JD. Stone artefact assemblages from Members 1-3, Swartkrans Cave. In: Brain C, editor. Swartkrans: a cave's chronicle of early man. Transvaal Museum Monograph No. 8. Pretoria: Transvaal Museum; 1993. 167-94.

27. Wells LH, Cooke HBS, Malan BD, Wells LH, Cooke HBS. The associated fauna and culture of the Vlakkraal Thermal Springs, O.F.S. Transactions of the Royal Society of South Africa 1942; 29(3): 203-33.

28. Mason RJ. Bone tools at the Kalkbank Middle Stone Age site and the Makapansgat Australopithecine locality, central Transvaal. Part 1: the Kalbank site. The South African Archaeological Bulletin 1958; 13; 85-93.

29. Meiring AJD. The macrolithic culture of Florisbad. Navorsinge van die Nasionale Museum: researches of the National Museum 1956; 1(9): 205-39.

30. Gruet M. Note préliminaire sur le gisement moustérien d'El Guettar. Bulletin de la Société préhistorique de France 1950; 47(5): 232-41.

31. Hocine S. Le site acheuléen d'Erg Tihodaïne : caractéristiques technologiques de l'industrie lithique du Pléistocène moyen (Sahara central, Algérie). Anthropologie. 2016 Jun; 120(3): 263-84.

32. Djemmali NE. L'industrie lithique acheuléenne du gisement de Tighennif (Ternifine), Algérie [doctoral thesis]. Paris, France: Muséum National d'Histoire Naturelle, Université Pierre et Marie Curie; 1985.

33. Sahnouni M. L'industrie sur galets du gisement Villafranchien Supérieur de Aïn Hanech (Sétif, Algérie Orientale) [doctoral thesis]. Paris, France: Muséum National d'Histoire Naturelle; 1985.

34. Mesfin I. Les assemblages lithiques Lupembiens conservés au Muséum National d'Histoire naturelle : apports et perspectives pour la connaissance du *Middle Stone Age* d'Afrique centrale [master thesis]. Paris, France: Muséum National d'Histoire Naturelle; 2018.

35. Le Tensorer JM, Von Falkenstein V, Le Tensorer H, Schmid P, Muhesen S. Etude préliminaire des industries archaïques de faciès Oldowayen du site de Hummal (El Kowm, Syrie centrale). Anthropologie. 2011; 115: 247-66.

36. Petraglia MD. The Lower Paleolithic of the Arabian Peninsula: occupations, adaptations, and dispersals. J World Prehist. 2003 Jun; 17(2): 141-79.

37. Shea JJ. Artifact abrasion, fluvial processes, and "living floors" from the Early Paleolithic site of 'Ubeidiya (Jordan Valley, Israel). Geoarchaeology: An International Journal 1999; 14(2): 191-207.

38. Güleç E, White T, Kuhn S, Özer I, Sagir M, Yilmaz H et al. The Lower Pleistocene lithic assemblage from Dursunlu (Konya), central Anatolia, Turkey. Antiquity 2009 Mar; 83(319): 11-22.

39. Sharon G, Feibel C, Alperson-Afil N, Harlavan Y, Feraud G, Ashkenazi S et al. New evidence for the Northern Dead Sea rift Acheulian. PaleoAnthropology Society 2010: 79-99.

40. Clark JD. Acheulian occupation sites in the Middle East and Africa: a study in cultural variability. Am Anthropol. 1966 Apr; 68(2): 202-29.

41. Yazbeck C. Les systèmes techniques de production au Paléolithique inférieur en Beqaa Libanaise : le cas de Joubb Jannine II [doctoral thesis]. Lyon, France: Université Lumière Lyon 2; 2002.

42. Barkai R, Gopher A. On anachronism: the curious presence of spheroids and polyhedrons at Acheulo-Yabrudian Qesem Cave, Israel. Quat Int. 2016 Apr; 398: 118-28.

43. Tchernov E, Horwitz LK, Ronen A, Lister A. The faunal remains from Evron Quarry in relation to other Lower Paleolithic hominid sites in the Southern Levant. Quat Res. 1994; 42: 328-39.

44. Bar-Yosef O. The lower Paleolithic of the Near East. J World Prehist. 1994 Sep; 8(3): 211-65.

45. Shemer M, Barzilai O. ‘Evron (East): preliminary report. Israel Antiquities Authority; 2017. 6p.

46. Gopher A, Barkai R, Shimelmitz R, Khalaily M, Lemorini C, Heshkovitz I et al. Qesem Cave: an Amudian site in Central Israel. Journal of the Israel Prehistoric Society 2005; 35: 69-92.

47. Stekelis M, Gilead D. Ma’ayan Barukh: a Lower Palaeolithic site in Upper Galilee. Mitekufat Haeven: Journal of the Israel Prehistoric Society 1966; 1-23.

48. Slimak L, Kuhn SL, Roche H, Mouralis D, Buitenhuis H, Balkan-Atli N et al. Kaletepe Deresi 3 (Turkey): archaeological evidence for early human settlement in Central Anatolia. J Hum Evol. 2008; 54: 99-111.

49. García-Vadillo FJ, Canals-Salomó A, Rodríguez-Alvarez XP, Carbonell-Roura E. The large flake Acheulian with spheroids from Santa Ana Cave (Cáceres, Spain). J Archaeol Sci Rep. 2022; 41: 103265.

50. Titton S, Barsky D, Bargalló A, Serrano-Ramos A, Vergès JM, Toro-Moyano I, et al. Subspheroids in the lithic assemblage of Barranco León (Spain): recognizing the late Oldowan in Europe. PLoS One. 2020; 15(1): e0228290.

51. Bourguignon L, Barsky D, Ivorra J, de Weyer L, Cuartero F, Capdevila R et al. The stone tools from stratigraphical unit 4 of the Bois-de-Riquet site (Lézignan-la-Cèbe, Hérault, France): a new milestone in the diversity of the European Acheulian. Quat Int. 2016; 411: 160-81.

52. Terradillos Bernal M, Moncel MH. Contribution à l’étude de la technologie du Paléolithique « archaïque » du sud de l’Europe selon le Système Logique Analytique (SLA). Application aux sites du Vallonnet (Roquebrune-Cap-Martin, France), de Gran Dolina TD6 (Burgos, Espagne), de Ca’Belvedere de Monte Poggiolo (Forli, Italie) et de Barranco Léon et Fuente Nueva 3 (Orce, Espagne). Anthropologie. 2004; 108: 307-29.

53. Fiedler L, Humburg C, Klingelhöfer H, Stoll S, Stoll M. Several Lower Palaeolithic sites along the Rhine Rift Valley, dated from 1.3 to 0.6 million years. Humanities 2019; 8(129).

54. Moncel MH, García-Medrano P, Despriée J, Arnaud J, Voinchet P, Bahain JJ. Tracking behavioral persistence and innovations during the Middle Pleistocene in Western Europe. Shift in occupations between 700 and 450 ka at la Noira site (Centre, France). J Hum Evol. 2021; 156: 103009.

55. Barsky D. Le débitage des industries lithiques de la Caune de l'Arago (Pyrénées-Orientales, France) : leur place dans l'évolution des industries du Paléolithique inférieur en Europe méditerranéenne [doctoral thesis]. Perpignan, France: Université de Perpignan; 2001.

56. Doronichev VB, Golovanova LV, Baryshnikov GF, Blackwell BAB, Garutt NV, Levkovskaya GM et al. Treugol’Naya Cave: the Early

Palaeolithic in Caucasus and Eastern Europe. St Petersburg; 2007.

57. Colonge D. Aquitaine, A65, Pyrénées-Atlantiques, Auriac, Duclos : Pléistocène moyen et Antiquité en Béarn. Inrap Grand Sud-Ouest; 2012. 476 p.

58. Fourloubey C. Aquitaine, A65, Landes, Cazères-sur-l'Adour: Septsos. Inrap Grand Sud-Ouest; 2012. 278 p.

59. Svoboda J, Valoch K, Cílek V, Oches E, McCoy W. Cervený Kopec (Red Hill): evidence for Lower Paleolithic occupations. Památky archeologické 1998; 89: 197-204.

60. Rodríguez Asensio JA, Flor Rodríguez G. Estudio del yacimiento prehistórico de Bañugues y su medio de depósito (Gozón, Asturias). Zephyrvs [Internet] 1979 [cited 2021 Oct 6]; 30. Available from: https://revistas.usal.es/index.php/0514-7336/article/view/1398

61. Cliquet D. Tourville-la-Rivière, Seine-Maritime: carrières et ballastières de Normandie : la Fosse-Marmitaine. Inrap Grand-Ouest; 2010. 105 p.

62. Claud É, Soressi M, Jaubert J, Hublin JJ. Étude tracéologique de l'outillage moustérien de type Quina du bonebed de Chez-Pinaud à Jonzac (Charente-Maritime). Nouveaux éléments en faveur d'un site de boucherie et de traitement des peaux. Gallia Préhistoire 2012; 54(1): 3-32.

63. Park SJ. Systèmes de production lithique et circulation des matières premières au Paléolithique moyen récent et final. Une approche techno-économique à partir de l'étude des industries lithiques de La Quina (Charente) [doctoral thesis]. Nanterre, France: Université Paris X; 2007.

64. Pittard E, de Saint-Périer RS. Les Festons, gisement paléolithique à Brantôme (Dordogne). Arch Suisses Anthropol Gen. 1955; 20(1-2): 1-141.

65. Agache R. Polyèdres subsphériques du levalloisien de Villers-Bocage et du Nord de la France. Bulletin de la Société préhistorique française 1958; 55(3-4): 216-9.

66. Collina-Girard J. Grille descriptive et évolution typologique des industries archaïques : le modèle catalan. Bulletin de la Société préhistorique française. 1986; 83(11-12) : 383-403.

67. Garcia Garriga. Las industrias arcaicas del Rosellón (Sur de Francia) : correlación geocronológica con las terrazas de los ríos Têt, Tech y Agly. Zephyrus 2014 Jan-Jun; 73: 17-43.

68. Gaillard C, Rajaguru, SN. Revisiting the Acheulian site of Singi Talav at Didwana (Rajasthan) 35 years later. In: Deo SG, Baptista A, Joglekar J, editors. Rethinking the past: a tribute to Professor V.N. Misra. Pune: Indian Society for Prehistoric and Quaternary Studies; 2017. p. 25-39.

69. Behera PK, Thakur N. Tanged points from the Middle Palaeolithic context at Torajunga, Bargarh Upland, Odisha, India. Man and Environment 2019; 44(1): 1-11.

70. Corvinus G. A survey of the Pravara river system in Western Maharashtra, India, vol. 2: the excavation of the Acheulian site of Chirki-on-Pravara, India. Tübingen: Archaeologica Vanatoria; 1983. 466 p.

71. Li F. An experimental study of bipolar reduction at Zhoukoudian locality 1, north China. Quat Int. 2016; 400: 23-9.

72. Bodin É. Analyse techno-fonctionnelle des industries à pièces bifaciales aux pléistocènes inférieur et moyen en Chine [doctoral thesis]. Nanterre, France: Université Paris X; 2011.

73. Yang SX, Huang WW, Hou YM, Yuan BY. Is the Dingcun lithic assembly a "chopper-chopping tool industry", or "Late Acheulian"? Quat Int. 2014; 321: 3-11.

74. Wang SJ, Lu HY**.** Taphonomic and paleoenvironmental issues of the Pleistocene loessic Paleolithic sites in the Qinling Mountains, central China. Sci China Earth Sci. 2016; 59(8): 1519-28.

75. Wang S, Lu H, Zhang H, Sun X, Yi S, Chen Y et al. Newly discovered Palaeolithic artefacts from loess deposits and their ages in

Lantian, central China. Chin Sci Bull. 2014; 59(7): 651-61.

76. Pei S, Niu D, Guan Y, Nian X, Yi M, Ma N et al. Middle Pleistocene hominin occupation in the Danjiangkou Reservoir region, central China: studies of formation processes and stone technology of Maling 2A site. J Archaeol Sci. 2015; 53: 391-407.

77. Li X, Ao H, Dekkers MJ, Roberts AP, Zhang P, Lin S et al. Early Pleistocene occurrence of Acheulian technology in North China. Quat Sci Rev. 2017; 156: 12-22.

78. Gao X. Explanations of typological variability in Paleolithic remains from Zhoukoudian locality 15, China [doctoral thesis]. Tucson, United States of America: University of Arizona; 2000.

79. Liu Y, Hu Y, Wei Q. Early to Late Pleistocene human settlements and the evolution of lithic technology in the Nihewan basin, North China: a macroscopic perspective. Quat Int. 2013; 295: 204-14.

80. Clark JD, Schick KD. Context and content: impressions of Palaeolithic sites and assemblages in the people's Republic of China. J Hum Evol. 1988; 17(4): 439-48.

81. Yang SX, Deng CL, Zhu RX, Petraglia MD. The Paleolithic in the Nihewan Basin, China: evolutionary history of an early to late Pleistocene record in Eastern Asia. Evol Anthrop. 2019; 29: 125-42.

82. Li H, Li ZY, Gao X, Kuman K, Summer A. Technological behaviour of the early Late Pleistocene archaic humans at Lingjing (Xuchang, China). Archaeol Anthropol Sci. 2019; 11(7): 3477-90.

83. Chi W. Searching for descendants of "Pecking man". Anthropol Anz. 1979; 37(2): 61-7.

84. De Lumley H, Cauche D, Celiberti V, Khatib S, Lartigot-Campin AS, Lebatard AE et al. Les industries du Paléolithique ancien de Corée du Sud dans leur contexte stratigraphique et paléoécologique: leur place parmi les cultures du Paléolithique ancien en Eurasie et en Afrique. Paris: CNRS éditions; 2011. 631 p.

85. Moncel MH, Arzarello M, Boëda É, Bonilauri S, Chevrier B, Gaillard C, et al. Assemblages with bifacial tools in Eurasia (second part). What is going on in the East? Data from India, Eastern Asia and Southeast Asia. C R Palevol. 2018 Jan; 17(1-2): 61-76.

86. Meilinda P. Temuan awal artefak di situs Banjarejo. Jurnal Sangiran 2017; 6: 26-39.

87. Fauzi MR, Ansyori MM, Prastiningtyas D, Intan MFS, Wibowo UP, Wulandari et al. Matar: a forgotten but promising Pleistocene locality in East Java. Quat Int. 2016; 416: 183-92.
