# Supplementary material for: Palaeolithic polyhedrons, spheroids and bolas over time and space
Source: PLoS One. 2022 Jul 28;17(7):e0272135. doi: 10.1371/journal.pone.0272135 (PMC9333226; doi:10.1371/journal.pone.0272135)
Supplement: S1 Text — (PDF) [file pone.0272135.s007.pdf]

S7. Data about PSBs: reference list, per region.

AFRICA:

Arroyo A, de la Torre I. Pounding tools in HWK EE and EF-HR (Olduvai Gorge, Tanzania): percussive activities in the Oldowan-Acheulean transition. J Hum Evol. 2018; 120: 402-21.

Assefa G, Clark JD, Williams MAJ. Late Cenozoic history and archaeology of the Upper Webi Shebele basin, East-Central Ethiopia. SINET: Ethiopian J Sci. 1982; 5(1): 27-46.

Berthelet A. L'outillage lithique du site de dépeçage à *Elephas recki ileretensis* de Barogali (république de Djibouti). C R Acad Sci IIa. 2001 Mar; 332(6): 411-6.

Berthelet A, Chavaillon J. Prehistoric archaeology. The site of Karre I: Karre I. In: Chavaillon J, Piperno M, editors. Studies on the Early Paleolithic site of Melka Kunture, Ethiopia. Florence: Origines; 2004. p. 211-51.

Chavaillon J. Prehistoric archaeology. The site of Gombore I: discovery, geological introduction and study of percussion material and tools on pebble. In: Chavaillon J, Piperno M, editors. Studies on the Early Paleolithic site of Melka Kunture, Ethiopia. Florence: Istituto Italiano di Preistoria e Protostoria; 2004. p. 253-369.

Chavaillon J, Berthelet A. The archaeological sites of Melka Kunture. In: Chavaillon J, Piperno M, editors. Studies on the Early Paleolithic site of Melka Kunture, Ethiopia. Florence: Istituto Italiano di Preistoria e Protostoria; 2004. p. 25-80.

Clark JD. Prehistory in southern Africa. In: Ki-Zerbo J, editor. General history of Africa, vol. 1: methodology and African Prehistory. London: Heinemann; 1981. p. 487-529.

Clark JD. Stone artefact assemblages from Members 1-3, Swartkrans Cave. In: Brain C, editor. Swartkrans: a cave's chronicle of early man. Transvaal Museum Monograph No. 8. Pretoria: Transvaal Museum; 1993. 167-94.

Clark JD, Kurashina H. New Plio-Pleistocene archaeological occurrences from the plain of Gadeb, Upper Webi Shebele basin, Ethiopia, and a statistical comparison of the Gadeb sites with other Early Stone Age assemblage. Anthropologie 1980; 18(2-3): 161-87.

Clément S. Les techniques de percussion : un reflet des changements techniques durant l'Acheuléen ? [doctoral thesis]. Nanterre, France: Université Paris X; 2019.

Cueva-Temprana A, Lombao D, Soto M, Itambu M, Bushozi P, Boivin N, Petraglia M, Mercader J. Oldowan technology amid shifting environments ~2.03-1.83 million years ago. Front Ecol Evol. 2022; 10: 788101.

D'Andrea A, Gallotti R, Piperno M. Taphonomic interpretation of the Developed Oldowan site of Garba IV (Melka Kunture, Ethiopia) through a GIS application. Antiquity 2002; 76: 991-1001.

De la Torre I. The Early Stone Age lithic assemblages of Gadeb (Ethiopia) and the Developed Oldowan/early Acheulean in East Africa. J Hum Evol. 2011; 60: 768-812.

De Weyer L. Etude technologique de l'industrie lithique du Ménié-Ménié, Ounjougou, Mali [master thesis]. Nanterre, France: Université Paris X; 2008.

De Weyer L. An Early Stone Age in Western Africa? Spheroids and polyhedrons at Ounjougou, Mali. Journal of Lithic Studies 2017; 4(1).

Diez-Martín F, Sánchez P, Domínguez-Rodrigo M, Mabulla A, Barba R. Were Olduvai hominins making butchering tools or battering tools? Analysis of a recently excavated lithic assemblage from BK (Bed II, Olduvai Gorge, Tanzania). J Anthropol Archaeol. 2009 Sep; 28(3): 274-89.

Djemmali NE. L'industrie lithique acheuléenne du gisement de Tighennif (Ternifine), Algérie [doctoral thesis]. Paris, France: Muséum National d'Histoire Naturelle, Université Pierre et Marie Curie; 1985.

Isaac GL, Isaac GL, Isaac B. Olorgesailie: archaeological studies of a Middle Pleistocene lake basin in Kenya. Chicago, United States of America: University of Chicago Press; 1977. 272 p.

Gallotti R. An older origin for the Acheulean at Melka Kunture (Upper Awash, Ethiopia): techno-economic behaviours at Garba IVD. J Hum Evol. 2013; 65: 594-620.

Gruet M. Note préliminaire sur le gisement moustérien d'El Guettar. Bulletin de la Société préhistorique de France 1950; 47(5): 232-41.

Hocine S. Le site acheuléen d'Erg Tihodaïne : caractéristiques technologiques de l'industrie lithique du Pléistocène moyen (Sahara central, Algérie). Anthropologie. 2016 Jun; 120(3): 263-84.

Howell FC. Isimila: a Paleolithic site in Africa. Scientific American 1961 Oct; 205(4): 118-31.

Kleindienst MR. Variability within the Late Acheulian assemblage in Eastern Africa. South African Archaeological Bulletin 1961 Jun; 16(62): 35-52.

Kleindienst MR. Components of the East African Acheulean assemblage: an analytic approach. In: Mortelmans G, editor. Actes du IVème Congrès Panafricain de Préhistoire et de l'Etude du Quaternaire 40. Tervuren: Musée Royal de l'Afrique Centrale; 1962. p. 81-105.

Kuman K, Sutton MB, Pickering TR, Heaton JL. The Oldowan industry from Swartkrans cave, South Africa, and its relevance for the African Oldowan. J Hum Evol. 2018; 123: 52-69.

Leakey LSB. The bolas in Africa. Man 1948; 48.

Leakey MD. Olduvai Gorge: vol. 3, excavations in Beds I and II, 1960-1963. Cambridge: Cambridge University Press; 1971. 328 p.

Mason RJ. Bone tools at the Kalkbank Middle Stone Age site and the Makapansgat Australopithecine locality, central Transvaal. Part 1: the Kalbank site. The South African Archaeological Bulletin 1958; 13(51): 85-93.

McNabb J, Sinclair A., Wadley L, Maguire J, Latham A, Herries A et al. The Cave of Hearths: Makapan Middle Pleistocene research project: field research by Antony Sinclair and Patrick Quinney, 1996-2001. Oxford: Archaeopress; 2009. 193 p.

Meiring AJD. The macrolithic culture of Florisbad. Navorsinge van die Nasionale Museum: researches of the National Museum 1956; 1(9): 205-39.

Mesfin I. Les assemblages lithiques lupembiens conservés au Muséum National d'Histoire Naturelle : apports et perspectives pour la connaissance du *Middle Stone Age* d'Afrique centrale [master thesis]. Paris, France: Muséum National d'Histoire Naturelle; 2018.

Mora R, de La Torre I. Percussion tools in Olduvai Beds I and II (Tanzania): implication for early human activities. J Anthropol Archaeol. 2005; 24: 179-92.

Mussi M, Altamura F, Di Bianco L, Bonnefille R, Gaudzinski-Windheuser S, Geraads D et al. After the emergence of the Acheulean at Melka Kunture (Upper Awash, Ethiopia): from Gombore IB (1.6 Ma) to Gombore Iγ (1.4 Ma), Gombore 1σ (1.3 Ma) and Gombore II OAM Test Pit C (1.2 Ma). Quat Int. Forthcoming.

Pante MC, de la Torre I. A hidden treasure of the Lower Pleistocene at Olduvai Gorge, Tanzania: the Leakey HWK EE assemblage. J Hum Evol. 2018 Jul; 120: 114-39.

Roche H, Brugal JP, Lefevre D, Ploux S, Texier PJ. Isenya: état des recherches sur un nouveau site acheuléen d'Afrique orientale. Afr Archaeol Rev. 1988; 6(1): 27-55.

Roche H, Texier PJ. Evaluation of technical competence of *Homo erectus* in East Africa during the Middle Pleistocene. In: Bower JRF, Sartono S, editors. Human evolution in its ecological context. Leiden: Royal Netherlands Academy of Arts and Sciences; 1996. p. 153-67.

Sahnouni M. L'industrie sur galets du gisement Villafranchien Supérieur de Aïn Hanech (Sétif, Algérie Orientale) [doctoral thesis]. Paris, France: Muséum National d'Histoire Naturelle; 1985.

Sahnouni M. Etude comparative des galets taillés polyédriques, subsphériques et sphériques des gisements d'Ain Hanech (Algérie orientale) et d'Olduvai (Tanzanie). Anthropologie. 1993; 97(1) : 51-68.

Santonja M, Panera J, Rubio-Jara S, Pérez-González A, Uribelarrea D, Domínguez-Rodrigo M et al. Technological strategies and the economy of raw materials in the TK (Thiongo Korongo) lower occupation, Bed II, Olduvai Gorge, Tanzania. Quat Int. 2014; 322-323: 181-208.

Sánchez-Yustos P, Diez-Martín F, Díaz I, Fraile C, Uribelarrea D, Mabulla A, et al. What comes after the Developed Oldowan B debate? Techno-economic data from SHK main site (Middle Bed II, Olduvai Gorge, Tanzania). Quat Int. 2019 Aug; 526: 67-76.

Walker N. Through the crystal ball: making sense of spheroids in the Middle Stone Age. The South African Archaeological Bulletin 2008 Jun; 63(187): 12-17.

Wells LH, Cooke HBS, Malan BD, Wells LH, Cooke HBS. The associated fauna and culture of the Vlakkraal Thermal Springs, O.F.S. Transactions of the Royal Society of South Africa 1942; 29(3): 203-33.

Willoughby PR. Spheroids and battered stones in the African Early Stone Age. World Archaeol. 1985; 17(1): 44-60.

Willoughby PR. Contribution à l'étude des sphéroïdes et des bolas de quelques sites paléolithiques d'Afrique. Anthropologie. 1990; 94(2): 241-58.

<u>LEVANT:</u>

Assaf E, Caricola I, Gopher A, Rosell J, Blasco R, Bar O, et al. Shaped stone balls were used for bone marrow extraction at Lower Paleolithic Qesem Cave, Israel. PLoS One. 2020; 15(4): e0230972.

Barkai R, Gopher A. On anachronism: the curious presence of spheroids and polyhedrons at Acheulo-Yabrudian Qesem Cave, Israel. Quat Int. 2016 Apr; 398: 118-28.

Bar-Yosef O. Locals and foreigners in the Levant during the Pleistocene. Anthropologie. 2021; 125: 102837.

Clark JD. Acheulian occupation sites in the Middle East and Africa: a study in cultural variability. Am Anthropol. 1966 Apr; 68(2): 202-29.

De la Torre I, Mora R. A technological analysis of non-flaked stone tools in Olduvai Beds I & II. Stressing the relevance of percussion activities in the African Lower Pleistocene. PALEO. Revue d'archéologie préhistorique (special issue) 2010; 13-34.

Güleç E, White T, Kuhn S, Özer I, Sagir M, Yilmaz H et al. The Lower Pleistocene lithic assemblage from Dursunlu (Konya), central Anatolia, Turkey. Antiquity 2009 Mar; 83(319): 11-22.

Le Tensorer JM, Von Falkenstein V, Le Tensorer H, Schmid P, Muhesen S. Etude préliminaire des industries des industries archaïques de faciès Oldowayen du site de Hummal (El Kowm, Syrie centrale). Anthropologie. 2011; 115: 247-66.

Petraglia MD. The Lower Paleolithic of the Arabian Peninsula: occupations, adaptations, and dispersals. J World Prehist. 2003 Jun; 17(2): 141-79.

Sharon G, Feibel C, Alperson-Afil N, Harlavan Y, Feraud G, Ashkenazi S et al. New evidence for the Northern Dead Sea rift Acheulian. PaleoAnthropology Society 2010: 79-99.

Shea JJ. Artifact abrasion, fluvial processes, and "living floors" from the Early Paleolithic site of 'Ubeidiya (Jordan Valley, Israel). Geoarchaeology: An International Journal 1999; 14(2): 191-207.

Shemer M, Barzilai O. 'Evron (East): Preliminary Report. Israel Antiquities Authority; 2017. 6p.

Slimak L, Kuhn SL, Balkan-Atli N, Binder D, Grenet M, Dinçer B. Kaletepe Deresi 3: de l'Acheuléen au Moustérien en Anatolie Centrale. Anatolia Antiqua, tome 15. 2007. p. 257-73.

Slimak L, Kuhn SL, Roche H, Mouralis D, Buitenhuis H, Balkan-Atli N et al. Kaletepe Deresi 3 (Turkey): archaeological evidence for early human settlement in Central Anatolia. J Hum Evol. 2008; 54: 99-111.

Stekelis M, Gilead D. Ma'ayan Barukh: a Lower Paleolithic site in Upper Galilee. Mitekufat Haeven: Journal of the Israel Prehistoric Society 1966; 1-23.

Tchernov E, Horwitz LK, Ronen A, Lister A. The faunal remains from Evron Quarry in relation to other Lower Paleolithic hominid sites in the Southern Levant. Quat Res. 1994; 42: 328-39.

Yazbeck C. Les systèmes techniques de production au Paléolithique inférieur en Beqaa Libanaise : le cas de Joubb Jannine II [doctoral thesis]. Lyon, France: Université Lumière Lyon 2; 2002.

Willoughby PR. Spheroids and battered stones in the African Early Stone Age. World Archaeol. 1985; 17(1): 44-60.

EUROPE:

Agache R. Polyèdres subsphériques du levalloisien de Villers-Bocage et du Nord de la France. Bulletin de la Société préhistorique française 1958; 55(3): 216-9.

Barsky D. Le débitage des industries lithiques de la Caune de l'Arago (Pyrénées-Orientales, France) : leur place dans l'évolution des industries du Paléolithique inférieur en Europe méditerranéenne [doctoral thesis]. Perpignan, France: Université de Perpignan; 2001.

Bourguignon L, Barsky D, Ivorra J, de Weyer L, Cuartero F, Capdevila R et al. The stone tools from stratigraphical unit 4 of the Bois-de-Riquet site (Lézignan-la-Cèbe, Hérault, France): a new milestone in the diversity of the European Acheulian. Quat Int. 2016; 411: 160-81.

Cliquet D. Tourville-la-Rivière, Seine-Maritime : carrières et ballastières de Normandie : la Fosse-Marmitaine. Inrap Grand-Ouest; 2010. 105 p.

Collina-Girard J. Grille descriptive et évolution typologique des industries archaïques : le modèle catalan. Bulletin de la Société préhistorique française. 1986; 83(11-12) : 383-403.

Colonge D. Aquitaine, A65, Pyrénées-Atlantiques, Auriac, Duclos : Pléistocène moyen et Antiquité en Béarn. Inrap Grand Sud-Ouest; 2012. 476 p.

Doronichev VB. The Lower Paleolithic in Eastern Europe and the Caucasus: a reappraisal of the data and new approaches. PaleoAnthropology 2008: 107-57.

Fiedler L, Franzen JL. Artefakte vom altpleistozänen Fundplatz “Dorn-Dürkheim 3" am nördlichen Oberrhein. Germania: nzeiger der Römisch-Germanischen Kommission des Deutschen Archäologischen Instituts. 2002; 80(2): 421-40.

Fiedler L, Humburg C, Klingelhöfer H, Stoll S, Stoll M. Several Lower Palaeolithic sites along the Rhine Rift Valley, dated from 1.3 to 0.6 million years. Humanities 2019; 8(129).

Fourloubey C. Aquitaine, A65, Landes, Cazères-sur-l’Adour: Septsos. Inrap Grand Sud-Ouest; 2012. 278 p.

García-Vadillo FJ, Canals-Salomó A, Rodríguez-Alvarez XP, Carbonell-Roura E. The large flake Acheulian with spheroids from Santa Ana Cave (Cáceres, Spain). J Archaeol Sci Rep. 2022; 41: 103265.

Lambert HJ. Sphéroïdes moustériens d’Artois et d’Île-de-France. Bulletin de la Société préhistorique de France 1944; 41(10-12): 177.

Moncel MH, García-Medrano P, Despriée J, Arnaud J, Voinchet P, Bahain JJ. Tracking behavioral persistence and innovations during the Middle Pleistocene in Western Europe. Shift in occupations between 700 and 450 ka at la Noira site (Centre, France). J Hum Evol. 2021; 156: 103009.

Park SJ. Systèmes de production lithique et circulation des matières premières au Paléolithique moyen récent et final. Une approche techno-économique à partir de l'étude des industries lithiques de La Quina (Charente) [doctoral thesis]. Nanterre, France: Université Paris X; 2007.

Pittard E, de Saint-Périer RS. Les Festons, gisement paléolithique à Brantôme (Dordogne). Arch Suisses Anthropol Gen. 1955; 20(1-2): 1-141.

Pittard E, Donici A. Les pierres de jet d'une station intermédiaire entre le Moustérien et l'Aurignacien (Dordogne). L'Homme Préhistorique 1927; 14(9-10): 209-19.

Rodríguez Asencio JA. Manifestaciones en Asturias del esferoide. Un útil del Paleolithic inferior. Zephyrvs 1976; 26-27: 85-95.

Rodríguez Asensio JA, Flor Rodríguez G. Estudio del yacimiento prehistórico de Bañugues y su medio de depósito (Gozón, Asturias). Zephyrvs [Internet] 1979 [cited 2021 Oct 6]; 30. Available from: https://revistas.usal.es/index.php/0514-7336/article/view/1398

Roussel M, Bourguignon L, Soressi M. Identification par l'expérimentation de la percussion au percuteur de calcaire au Paléolithique moyen : le cas du façonnage des racloirs bifaciaux Quina de Chez Pinaud (Jonzac, Charente-Maritime). Bulletin de la Société préhistorique française 2009 Apr; 106(2): 219-38.

Svoboda J, Valoch K, Cílek V, Oches E, McCoy W. Cervený Kopec (Red Hill): evidence for Lower Paleolithic occupations. Památky archeologické 1998; 89: 197-204.

Titton S, Barsky D, Bargalló A, Serrano-Ramos A, Vergès JM, Toro-Moyano I, et al. Subspheroids in the lithic assemblage of Barranco León (Spain): recognizing the late Oldowan in Europe. Petraglia MD, editor. PLoS One. 2020; 15(1): e0228290.

ASIA:

Behera PK, Thakur N. Tanged points from the Middle Palaeolithic context at Torajunga, Bargarh Upland, Odisha, India. Man and Environment 2019; 44(1): 1-11.

Bodin É. Analyse techno-fonctionnelle des industries à pièces bifaciales aux pléistocènes inférieur et moyen en Chine [doctoral thesis]. Nanterre, France: Université Paris X; 2011.

Chi W. Searching for descendants of "Pecking man". Anthropol Anz. 1979; 37(2): 61-7.

Corvinus G. A survey of the Pravara river system in western Maharashtra, India, vol. 2: the excavation of the Acheulian site of Chirki-on-Pravara, India. Tübingen: Archaeologica Vanatoria; 1983. 466 p.

De Lumley H, Cauche D, Celiberti V, Khatib S, Lartigot-Campin AS, Lebatard AE et al. Les industries du Paléolithique ancien de Corée du Sud dans leur contexte stratigraphique et paléoécologique: leur place parmi les cultures du Paléolithique ancien en Eurasie et en Afrique. Paris: CNRS éditions; 2011. 631 p.

Fauzi MR, Ansyori MM, Prastiningtyas D, Intan MFS, Wibowo UP, Wulandari et al. Matar: a forgotten but promising Pleistocene locality in East Java. Quat Int. 2016; 416: 183-92.

Gaillard C, Rajaguru, SN. Revisiting the Acheulian site of Singi Talav at Didwana (Rajasthan) 35 years later. In: Deo SG, Baptista A, Joglekar J, editors. Rethinking the past: a tribute to Professor V.N. Misra. Pune: Indian Society for Prehistoric and Quaternary Studies; 2017. p. 25-39.

Gao X. Explanations of typological variability in Paleolithic remains from Zhoukoudian locality 15, China [doctoral thesis]. Tucson, United States of America: University of Arizona; 2000.

Joglekar J, Deo SG. Artefactual evidence of early hominin adaptability in the Deccan Trap region of the Upper Krishna basin. In: Deo SG, Baptista A, Joglekar J, editors. Rethinking the past: a tribute to Professor VN Misra. Pune: Indian Society for Prehistoric and Quaternary studies; 2017. p. 16-24.

Li F. An experimental study of bipolar reduction at Zhoukoudian locality 1, North China. Quat Int. 2016; 400: 23-29.

Li X, Ao H, Dekkers MJ, Roberts AP, Zhang P, Lin S et al. Early Pleistocene occurrence of Acheulian technology in North China. Quat Sci Rev. 2017; 156: 12-22.

Li H, Li ZY, Gao X, Kuman K, Summer A. Technological behaviour of the early Late Pleistocene archaic humans at Lingjing (Xuchang, China). Archaeol Anthropol Sci. 2019; 11(7): 3477-90.

Liu Y, Hu Y, Wei Q. Early to Late Pleistocene human settlements and the evolution of lithic technology in the Nihewan basin, North China: a macroscopic perspective. Quat Int. 2013; 295: 204-14.

Moncel MH, Arzarello M, Boëda É, Bonilauri T, Chevrier B, Gaillard C, et al. Assemblages with bifacial tools in Eurasia (second part). What is going on in the East? Data from India, Eastern Asia and Southeast Asia. C R Palevol. 2018 Jan; 17(1-2): 61-76.

Pei S, Niu D, Guan Y, Nian X, Yi M, Ma N et al. Middle Pleistocene hominin occupation in the Danjiangkou Reservoir region, central China: studies of formation processes and stone technology of Maling 2A site. J Archaeol Sci. 2015; 53: 391-407.

Wang S, Lu H, Zhang H, Sun X, Yi S, Chen Y et al. Newly discovered Palaeolithic artefacts from loess deposits and their ages in Lantian, central China. Chin Sci Bull. 2014; 59(7): 651-61.

Wang S, Lu H. Taphonomic and paleoenvironmental issues of the Pleistocene loessic Pleolithic sites in the Qinling Mountains, central China. Sci China Earth Sci. 2016; 59(8): 1519-28.

Yang SX, Huang WW, Hou YM, Yuan BY. Is the Dingcun lithic assembly a “chopper-chopping tool industry”, or “Late Acheulian”? Quat Int. 2014; 321: 3-11.

Yang SX, Deng CL, Zhu RX, Petraglia MD. The Paleolithic in the Nihewan Basin, China: evolutionary history of an early to late Pleistocene record in Eastern Asia. Evol Anthrop. 2019; 29: 125-42.
