# Supplementary material for: Palaeolithic polyhedrons, spheroids and bolas over time and space
Source: PLoS One. 2022 Jul 28;17(7):e0272135. doi: 10.1371/journal.pone.0272135 (PMC9333226; doi:10.1371/journal.pone.0272135)
Supplement: S2 Text — (PDF) [file pone.0272135.s008.pdf]

S8. List of adjustments made for the construction of some pie charts in the figure 8.

Note: assemblages for which the quantity of PSBs according to raw materials could not be approximated are not mentioned here. These assemblages correspond to totally or partially white pie charts in the figure 8, and to assemblages that do not appear on the maps in this figure.

Olorgesailie

Facts: most of PSBs are in basalt, few others in other types of lava and some large PSBs are in quartz (Isaac et al. 1977 [1], Willoughby 1990 [2])

Adjustments for the figure 8: 90% of PSBs in igneous rocks, 10% in quartz.

Melka Kunturé (Karre levels K1-2)

Facts: PSBs are in basalt and trachyte (Berthelet & Chavaillon 2004 [3]).

Adjustments for the figure 8: 100% of PSBs in igneous rocks.

Melka Kunturé (Garba IV)

Facts: PSBs in volcanic rocks (most likely basalt, maybe trachyte and tuff) (d'Andrea et al. 2002 [4]).

Adjustments for the figure 8: 100% of PSBs in igneous rocks.

Melka Kunturé (Gombore IB)

Facts: PSBs mainly in basalt, but also some in obsidian and lava; trachyte or tuff (Chavaillon 2004 [5]).

Adjustments for the figure 8: 100% of PSBs in igneous rocks.

Melka Kunturé (Gombore Iγ)

Facts: PSBs in volcanic rocks (basalt, maybe obsidian or others) (Chavaillon & Berthelet 2004 [6]).

Adjustments for the figure 8: 100% of PSBs in igneous rocks.

Melka Kunturé (Gombore II, localities 1-5)

Facts: PSBs are in basalt, maybe some in obsidian. At locality 2, some could also be in vuggular lava (data about the assemblages: Chavaillon & Berthelet 2004 [6]).

Adjustments for the figure 8: 100% of PSBs in igneous rocks.

Melka Kunturé (Simbiro III, levels A, B, D)

Facts: at Simbiro III A, pebble tools are made in basalt or tuff. At Simbiro III B and D, PSBs are made in volcanic rocks (Chavaillon & Berthelet 2004 [6]).

Adjustments for the figure 8: 100% of PSBs in igneous rocks.

Isenya (level V)

Facts: according to the preliminary report Roche et al. 1988 [7], there are 62 PSBs (n=21 bolas “percuteurs, bolas”, n=29 spheroids, n=12 polyhedrons) mainly in phonolite nephelinic. Less commonly, they are in phonolite with biotite, quartz and quartzite. Bolas are generally in quartz.

Adjustments for the figure 8: 65% of PSBs in phonolite, 25% in quartz, 10% in quartzite.

Joubb Jannine II

Facts: spheroids (n=2) are in flint, polyhedrons (n=255) in limestone and flint (Yazbeck 2002 [8]).

Adjustments for the figure 8: 70% of PSBs in limestone, 30% in flint. Indeed, we chose to attribute a highest proportion of PSBs in limestone than in flint, since PSBs in limestone are widely more common, especially in this region at this period. PSBs in flint are very much rarer.

Caune de l’Arago

Facts: PSBs are nearly exclusively made from quartz, but there are also few in quartzite. « Les polyèdres ou sphéroïdes ont été réalisés presque exclusivement en quartz. La seule autre roche utilisée en quantité relativement importante est le quartzite. » (Barsky 2001 [9]).

Adjustments for the figure 8: 90% of PSBs in quartz, 10% in quartzite.

Dingcun

Facts: most of PSBs (n=13 PSBs in the assemblage, Yang et al. 2014 [10]) are in limestone, the rest in quartz, green sandstone and quartzite (Bodin 2011 [11]).

Adjustments for the figure 8: among the 13 PSBs 7/13 are in limestone, 2 in quartz, 2 in quartzite, 2 in sandstone.

References

1. Isaac GL, Isaac GL, Isaac B. Olorgesailie: archaeological studies of a Middle Pleistocene lake basin in Kenya. Chicago: University of Chicago Press; 1977. 272 p.
2. Willoughby PR. Contribution à l'étude des sphéroïdes et des bolas de quelques sites paléolithiques d'Afrique. Anthropologie. 1990; 94(2): 241-58.
3. Berthelet A, Chavaillon J. Prehistoric archaeology. The site of Karre I: Karre I. In: Chavaillon J, Piperno M, editors. Studies on the Early Paleolithic site of Melka Kunture, Ethiopia. Florence: Origines; 2004. p. 211-51.
4. D’Andrea A, Gallotti R, Piperno M. Taphonomic interpretation of the Developed Oldowan site of Garba IV (Melka Kunture, Ethiopia) through a GIS application. Antiquity 2002; 76: 991-1001.
5. Chavaillon J. Prehistoric archaeology. The site of Gombore I: discovery, geological introduction and study of percussion material and tools on pebble. In: Chavaillon J, Piperno M, editors. Studies on the Early Paleolithic site of Melka Kunture, Ethiopia. Florence: Istituto Italiano di Preistoria e Protostoria; 2004. p. 253-369.
6. Chavaillon J, Berthelet A. The archaeological sites of Melka Kunture. In: Chavaillon J, Piperno M, editors. Studies on

the Early Paleolithic site of Melka Kunture, Ethiopia. Florence: Istituto Italiano di Preistoria e Protostoria; 2004. p. 25-80.

7. Roche H, Brugal JP, Lefevre D, Ploux S, Texier PJ. Isenya: état des recherches sur un nouveau site acheuléen d'Afrique orientale. Afr Archaeol Rev. 1988; 6(1): 27-55.

8. Yazbeck C. Les systèmes techniques de production au Paléolithique inférieur en Beqaa Libanaise : le cas de Joubb Jannine II [doctoral thesis]. Lyon, France: Université Lumière Lyon 2; 2002.

9. Barsky D. Le débitage des industries lithiques de la Caune de l'Arago (Pyrénées-Orientales, France) : leur place dans l'évolution des industries du Paléolithique inférieur en Europe méditerranéenne [doctoral thesis]. Perpignan, France: Université de Perpignan; 2001.

10. Yang SX, Huang WW, Hou YM, Yuan BY. Is the Dingcun lithic assembly a "chopper-chopping tool industry", or "Late Acheulian"? Quat Int. 2014; 321: 3-11.

11. Bodin É. Analyse techno-fonctionnelle des industries à pièces bifaciales aux pléistocènes inférieur et moyen en Chine [doctoral thesis]. Nanterre, France: Université Paris X; 2011.
